# Personal Relative Deprivation and Locus of Control

**DOI:** 10.1111/jopy.12980

**Published:** 2024-10-22

**Authors:** Mitchell J. Callan, Robbie M. Sutton, Phatthanakit Chobthamkit, Victoria Wai Lan Yeung, Florence Y. N. Leung, Ryosuke Asano, Peter Beattie, Allan B. I. Bernardo, Chinun Boonroungrut, Jen‐Ho Chang, Anindita Chaudhuri, Chin‐Lung Chien, Hoon‐Seok Choi, Lixian Cui, Hongfei Du, Alexander Scott English, Kei Fuji, Hidefumi Hitokoto, Junko Iida, Keiko Ishii, Ding‐Yu Jiang, Yashpal Jogdand, Hyejoo J. Lee, Nobuhiro Mifune, Aya Murayama, Jinkyung Na, Kim One, Joonha Park, Kosuke Sato, Punit Shah, Suryodaya Sharma, Eunkook M. Suh, Arun Tipandjan, Michael Shengtao Wu, William J. Skylark

**Affiliations:** ^1^ Department of Psychology University of Bath Bath UK; ^2^ School of Psychology University of Kent Canterbury UK; ^3^ Department of Psychology Thammasat University Pathumthani Thailand; ^4^ Department of Psychology Lingnan University Hong Kong; ^5^ Department of Psychology Kurume University Kurume Japan; ^6^ MGPE Programme, School of Governance and Policy Science The Chinese University of Hong Kong Hong Kong; ^7^ Department of Psychology De La Salle University Manila Philippines; ^8^ Department of Educational Psychology and Guidance Silpakorn University Nakhon Pathom Thailand; ^9^ Institute of Ethnology Academia Sinica Taipei Taiwan; ^10^ Department of Psychology University of Calcutta Kolkata India; ^11^ Department of Psychology Soochow University Taipei Taiwan; ^12^ Department of Psychology Sungkyunkwan University Seoul South Korea; ^13^ Division of Arts and Sciences NYU Shanghai Shanghai China; ^14^ Department of Psychology Beijing Normal University at Zhuhai Zhuhai China; ^15^ Department of Psychology Wenzhou‐Kean University Wenzhou China; ^16^ Division of Psychology, Institute of Human Sciences University of Tsukuba Tsukuba Japan; ^17^ Department of Psychological Sciences Kwansei Gakuin University Nishinomiya Hyogo Japan; ^18^ Department of Cognitive and Psychological Sciences Nagoya University Nagoya Japan; ^19^ Department of Psychology National Chung‐Cheng University Chiayi Taiwan; ^20^ Department of Humanities and Social Sciences Indian Institute of Technology Delhi New Delhi India; ^21^ Department of Psychology Handong Global University Pohang South Korea; ^22^ School of Economics & Management Kochi University of Technology Kochi Japan; ^23^ College of Comprehensive Psychology Ritsumeikan University Ibaraki Japan; ^24^ Department of Psychology Sogang University Seoul South Korea; ^25^ Zaehoon Language Institute Bangkok Thailand; ^26^ Graduate School of Education Kyoto University Kyoto Japan; ^27^ Department of Humanities and Social Sciences Krea University Sricity India; ^28^ Department of Psychology Yonsei University Seoul South Korea; ^29^ International Centre for Psychological Counseling and Social Research Puducherry India; ^30^ School of Journalism and Communication Xiamen University Xiamen China; ^31^ Department of Psychology University of Cambridge Cambridge UK

**Keywords:** locus of control, personal relative deprivation, sense of control, social status

## Abstract

**Objective:**

We investigated the relationship between personal relative deprivation (PRD)—resentment from the belief that one is worse off than people who are similar to oneself—and locus of control.

**Background:**

Research has yet to comprehensively investigate whether PRD is associated with a tendency to favor external (vs. internal) explanations for self‐ and other‐relevant outcomes.

**Method:**

Eight studies (*N*_total_ = 6729) employed cross‐sectional, experimental, and (micro)longitudinal designs and used established trait and state measures of PRD and loci of control.

**Results:**

Participants higher in PRD adopted more external (vs. internal) explanations for others' outcomes while controlling for socio‐demographics (e.g., socioeconomic status; Studies 1–4). This relationship was mediated by a lowered sense of personal control (Study 1) and evident in a cross‐national sample of participants in Asia (Study 2). PRD is more robustly associated with external than internal explanations for self and other‐relevant outcomes (Studies 5–8), and within‐person changes in PRD are positively associated with within‐person changes in external explanations (month‐to‐month and day‐to‐day; Studies 7–8).

**Conclusions:**

PRD is positively associated with external locus of control independent of socioeconomic status, within and between people, and across cultures. This research highlights the implications of PRD for people's construal of the causal forces that govern their lives.

## 1 Introduction

People differ in their tendency to favor internal versus external explanations for self‐ and other‐relevant outcomes. The extent to which people favor internal or external explanations is often referred to as *locus of control* (Rotter 1966). Individuals with an *external* locus of control believe that external factors—such as luck, fate, other people, or situational circumstances—govern the events and outcomes in people's lives. In contrast, individuals with an *internal* locus of control believe that events and outcomes are contingent on one's own abilities, efforts, or choices. According to Rotter's social learning theory (1954, 1966), an individual's locus of control is acquired, reinforced, and strengthened through their experiences of the contingencies or independence between what they do and what happens to them, with a more external locus of control developing among individuals who perceive their own outcomes as resulting from external forces beyond their personal control.

Thousands of studies have examined the correlates of locus of control beliefs, such as mental well‐being (e.g., Presson and Benassi 1996), physical health (Cheng, Cheung, and Lo 2016), academic achievement (Findley and Cooper 1983), and motivation in the workplace (e.g., Wang, Bowling, and Eschleman 2010). This body of research has revealed that, in general, people with a stronger internal locus of control tend to be happier and healthier than their more externally‐oriented counterparts (for reviews, see, e.g., Galvin et al. 2018; Lefcourt 1982; Nowicki and Duke 2016). Consequently, it is important to understand the factors that are associated with people adopting a more external locus of control. The present paper seeks a new perspective on this question by examining whether and how locus of control is associated with *personal relative deprivation (PRD)*—that is, dissatisfaction stemming from the belief that one is worse off than people who are similar to oneself.

### 1.1 Antecedents of Individual Differences in Locus of Control

Although the consequences of locus of control beliefs continue to garner considerable research attention, less research has examined the potential antecedents of individual differences in locus of control. Most relevant to the current investigation, research has revealed that socioeconomic status (SES) is an important predictor of the development of a person's belief in internal versus external locus of control. Adults lower in SES (e.g., Kraus, Piff, and Keltner 2009; Powell and Vega 1972) and children and adolescents raised in families experiencing socioeconomic challenges (e.g., Battle and Rotter 1963; Culpin et al. 2015) tend to have a more external locus of control. Mirowsky and Ross (1990; see also Ross and Mirowsky 1989; Kraus et al. 2012) asserted that people of different SES backgrounds experience different material and social conditions that vary in conduciveness to the development of a sense of personal control. People higher in SES are socialized in environments characterized by abundant financial resources, more personal freedoms, more social opportunities, fewer daily stressors, and greater job autonomy. These experiences enable people higher in SES to link their positive outcomes more readily to their own efforts, choices, and abilities, hence fostering a belief that outcomes are contingent on one's own choices and actions.

Conversely, people lower in SES may adopt an external locus of control from repeated experiences of weak contingencies between their actions and rewards or punishments, due to inadequate financial resources, lack of social opportunities, heightened social and environmental stressors (e.g., crime, unemployment, and pollution), and occupying jobs with limited autonomy. As Rotter (1966, 3) noted, repeatedly feeling like a “small cog in a big machine and at the mercy of forces too strong or too vague to control” is likely to foster an external locus of control. Consistent with this analysis, Kraus, Piff, and Keltner (2009) found that people lower in SES tended to adopt more external explanations for others' good and bad outcomes (e.g., getting into medical school, having low income), and this relationship was mediated by a reduced sense of *personal* control. These findings suggest that the belief that others' successes or failures are caused by external factors represents a reduction in the sense of personal control, engendered by experiences associated with having lower SES.

### 1.2 Personal Relative Deprivation

Without disputing the importance of SES to the development of one's locus of control, in the current research we explored another potential social rank‐based correlate of locus of control beliefs: PRD. PRD refers to the sense of unfairness and feeling of resentment that arise when an individual believes that they are deprived of a desired outcome relative to their peers (Crosby 1976; Smith et al. 2012; Smith, Pettigrew, and Huo 2020). Specifically, according to an influential model proposed by Smith et al. (2012), the experience of PRD stems from a process whereby a person makes an unfavorable social comparison with a given target (e.g., a co‐worker) on a given outcome (e.g., salary), believes themselves to be unfairly disadvantaged, and consequently feels dissatisfied and resentful. Correspondingly, Kim, Callan et al. (2018) found that when people made upward social comparisons of affluence with self‐chosen targets possessing the same background characteristics (e.g., educational qualifications), they experienced elevated dissatisfaction and resentment, and this effect was mediated by perceived unfairness.

PRD is related to, but distinct from, SES (e.g., income, education, and occupational prestige)—a person can be objectively or subjectively high in SES but still feel dissatisfied with how their material circumstances compare with those of their peers (cf. Stouffer et al. 1949). For example, a highly educated and highly paid marketing manager may resent the corner office of an equally qualified and productive colleague. Conversely, a lowly paid retail clerk may feel perfectly sanguine about their material circumstances when they think about what they have compared with their unemployed classmates from school. Correspondingly, Callan, Kim, and Matthews (2015a) found that unfavorable social comparisons of affluence predicted participants' feelings of resentment regardless of their objective SES or subjective SES (i.e., self‐perceived rank within the national population). Thus, people's sense of fairness and satisfaction with their “lot in life” are shaped at least as much by local, specific, interpersonal comparisons of affluence as they are by objective or subjective SES (Crosby 1976; Smith et al. 2012; Manstead, Easterbrook, and Kuppens 2020; Zell and Alicke 2010).

Importantly, PRD in turn predicts a range of economic, social, and well‐being outcomes over and above the influences of objective and/or subjective SES indicators (for reviews, see Smith et al. 2012; Smith, Pettigrew, and Huo 2020). These outcomes include, among others, reduced prosociality (Gheorghiu, Callan, and Skylark 2021), lower physical and psychological well‐being (Callan, Kim, and Matthews 2015a), increased aggression (Greitemeyer and Sagioglou 2019), and increased materialism (Kim et al. 2017). For example, Callan et al. (2017) found that, controlling for objective and subjective SES indicators, participants higher in PRD were less willing to act for the benefit of others. Likewise, Callan, Kim, and Matthews (2015a) found that PRD was a more dominant predictor of psychological and physical well‐being than subjective SES, annual household income, and educational attainment. Furthermore, the associations between PRD and its putative outcomes do not vary meaningfully by different levels of SES indicators—that is, unfavorable social comparisons of affluence with one's peers tend to affect people higher in SES as much as they do for people lower in SES (e.g., Callan, Kim, and Matthews 2015a; Kim et al. 2017).

### 1.3 PRD and Locus of Control

The foregoing analysis suggests that PRD is distinct from objective and subjective SES and often predicts outcomes over and above these variables. In her seminal review of the relative deprivation literature, Crosby (1976) hypothesized that locus of control serves as a key mediator in the link between PRD and its putative outcomes (e.g., aggression and stress symptoms), but surprisingly little research since Crosby's review has investigated whether PRD is associated with locus of control beliefs. Crosby's analysis and later research indicate that local, specific social comparisons with salient similar others tend to have a greater influence on self‐perception and self‐evaluation than objective criteria or comparisons with aggregates and larger samples (e.g., Gerber, Wheeler, and Suls 2018; Zell and Alicke 2010). Informed by this theory and evidence, we posit that an individual's locus of control may not just be a product of their objective SES but also a product of the social comparison processes that are involved in the experience of PRD.

One reason to surmise a relationship between PRD and greater externality is that, much like having lower SES, being deprived relative to one's peers is aversive (e.g., Beshai et al. 2017; Callan et al. 2015; Tougas et al. 2005; Walker and Mann 1987). Repeatedly experiencing adverse events has been found to promote the adoption of an external locus of control (for reviews, see Carton and Nowicki 1996; Nowicki 1978; Nowicki et al. 2018). Thus, an external locus of control would be expected to develop in those individuals with a greater tendency to experience PRD, such as among individuals with a tendency to make frequent social comparisons (Callan et al. 2015; Kim et al. 2017, 2018) and/or who find themselves in social environments that constantly impose unfavorable social comparisons of affluence (e.g., the workplace, one's local neighborhood).

In addition, the nature of the underlying comparison and judgment processes involved in the experience of PRD may foster an external locus of control. By definition, PRD requires social comparison. Since comparing oneself with similar others provides the most diagnostic and relevant information for self‐evaluation (Festinger 1954; Wood 1989), people tend to compare themselves with others who are similar in terms of the background abilities, characteristics, and attributes (e.g., skill, motivation, and educational qualifications) that influence a given outcome (e.g., one's salary). Correspondingly, Kim, Callan et al. (2018) found that when asked to recall spontaneously a target person who they usually compare themselves with in terms of their material and financial circumstances, a large majority of participants (79%) identified someone whom they perceived to be more similar than dissimilar in terms of their background characteristics (e.g., personality, age, and personal interests).

This tendency to focus on similar others in the comparison process inherently limits the range of *internal* explanations available to the comparer when trying to make sense of perceived disadvantages when they arise (cf. Wedell and Parducci 2000). Put differently, differences between the self and target in, for example, abilities, skills, qualifications, or motivation—that is, *internal* factors—that might otherwise help the comparer explain their relative disadvantage (e.g., “she got promoted over me because she works harder and is better qualified”) are limited by the tendency for more similar than dissimilar targets to be included in one's comparison set in the first place. With the availability of internal attributions relatively constrained by the comparison process, the comparer is left with entertaining causes for their relative disadvantage that are *external* to the self, such as luck, fate, or powerful others (e.g., “she got promoted over me because her uncle is pals with the manager”). This reasoning is consistent with Rotter's (1954, 1966) social learning theory, which posits that personal experiences reinforce one's beliefs about the level of control they do or do not have over their outcomes, leading to expectancies that outcomes are governed by personal choices and attributes or external factors. Here, when individuals see others achieving more favorable outcomes despite similar background characteristics, they might attribute these differences to external factors, thus fostering an external locus of control.

As an example of this process, imagine two co‐workers—say, our two marketing managers above—who have similar background qualifications and job performance but different job perks (corner office with a view or not). The individual who feels relatively deprived—despite not being socioeconomically deprived—might attribute this disparity in job perks to external factors like favoritism, because internal explanations (e.g., effort, skill, and performance) might seem inadequate due to their perceived background similarities. This might reinforce the deprived individual's belief that external forces govern career outcomes, fostering an external locus of control over time.

It is important to highlight that this process is different from how SES is believed to contribute to locus of control beliefs. SES is associated with internal or external locus of control orientations because people from different social class backgrounds experience different material, structural, and social conditions that enhance or limit their capacity to affect the contingencies between what they do and what happens to them (e.g., Kraus et al. 2012). The novel theoretical perspective we offer here is that the potential link between PRD and locus of control is not a property of one's objective or subjective social‐structural position per SE but rather a property of the cognitive appraisals involved in comparing oneself to similar others experiencing more favorable outcomes.

### 1.4 Overview of Current Research

Across eight studies, we tested the general hypothesis that, like having low socioeconomic status, higher PRD is associated with a stronger external locus of control (and, equivalently, with a less internal locus of control). Rotter's (1966) theory suggests that people generalize their personal experiences of control (or lack thereof) to broader contexts, leading them to believe that others in general have similar levels of control (Johnson et al. 2015; cf. the consensus effect, which involves the tendency for people to overestimate how much others share their beliefs, values, and behaviors; see Marks and Miller 1987; Mullen et al. 1985; Ross, Greene, and House 1977). Indeed, people's beliefs about whether they can personally control their environment tend to align with their beliefs about others' control capabilities (e.g., Kraus, Piff, and Keltner 2009; Gore, Griffin, and McNierney 2016). For example, as noted above, Kraus et al. found that the tendency for people lower in subjective socioeconomic status (SSS) to adopt more external explanations for various social outcomes (e.g., publishing a book and having low income) was mediated by corresponding personal control beliefs (e.g., “Other people determine most of what I can and cannot do”). We therefore expected that higher PRD would be associated with a stronger external locus of control for both self and other‐relevant outcomes. We adopted a variety of measures that gauge people's tendency to favor external (vs. internal) explanations for self‐ and other‐relevant outcomes, including explanations for others' good and bad outcomes in general (Studies 1 to 5), explanations for the outcomes of self‐selected peers (Study 6), and explanations for self‐relevant outcomes and circumstances (Studies 1, 7, and 8).

Study 1 investigated whether people higher in PRD tend to adopt more external (vs. internal) explanations for broad positive and negative social outcomes, and whether this relationship is accounted for by a lowered sense of personal control (cf. John, Boileau, and Bless 2024; Kraus, Piff, and Keltner 2009). Study 2 aimed to cross‐culturally replicate the link between PRD and external (vs. internal) explanations in a large sample of participants from 18 regions across Asia. Studies 3 and 4 tested the generalizability of our findings to different domain‐general and domain‐specific operationalizations of loci of control. Study 5 aimed to clarify the relative importance of external (vs. internal) explanations in the association between PRD and loci of control by treating internal and external loci of control beliefs as correlated but distinct constructs rather than falling along a single, bipolar internal‐to‐external continuum (cf. Gatz and Good 1978; Gore, Griffin, and McNierney 2016). Importantly, across Studies 1 to 5 we measured control variables that theoretically might confound the relationship between PRD and locus of control, including indicators of subjective and objective SES, political orientation, age, and gender. We thus examined whether individual differences in PRD correlate with internal versus external explanations, and whether PRD predicts loci of control after controlling for other variables that have been found to associate with PRD (e.g., PRD is negatively associated with subjective SES; Callan et al. 2015, 2017) and one's locus of control (e.g., people who are politically liberal tend to have a stronger external locus of control; e.g., Kraus, Piff, and Keltner 2009).

In Studies 6 to 8, we shift our focus to testing the causal relationships among PRD and internal versus external explanations. In Study 6, we experimentally induced unfavorable versus favorable social comparisons of affluence with similar others and gauged participants' external and internal explanations for their comparison targets' financial standing. Studies 7 and 8 investigated longitudinally (month‐to‐month and day‐to‐day, respectively) the reciprocal within‐person associations among PRD and external versus internal personal control beliefs. Taken together, the key contribution of the present studies lies in furthering our understanding of how perceived relative disadvantage is associated with, and potentially affects, the tendency to favor external explanations for self‐ and other‐relevant outcomes across a broad range of circumstances.

### 1.5 Transparency and Openness

All data, materials, and analysis syntax are available at https://osf.io/hfd5e/?view_only=cae7c5adf7624a539799e5d8958bf84d. The sampling and analysis plans for Studies 3 to 8 were preregistered; links to the preregistration documents are provided within the individual study descriptions. Analyses were performed using R (version 4.3.1; R Core Team 2023). The individual R packages used for analyses are referenced throughout the paper where relevant. This work was approved by the University of Bath's Psychology Research Ethics Committee (approval 19‐219).

## 2 Study 1

In Study 1, we examined whether (a) PRD is associated with external explanations for others' good and bad outcomes (e.g., getting into medical school and having low income) controlling for indicators of objective and subjective SES, political orientation, and socio‐demographics (i.e., age and gender), and (b) the relationship between PRD and external explanations is accounted for by a lowered sense of personal control (cf. Kraus, Piff, and Keltner 2009).

### 2.1 Method

#### 2.1.1 Participants

Participants were recruited via Amazon's Mechanical Turk (MTurk). We requested 500 participants from the United States. We discarded without analysis responses from participants who did not answer all questions, who indicated an age less than 18 or greater than 100, or whose IP address occurred earlier in the data set (in the case of overlapping timestamps, both were excluded). We excluded 27 participants who failed the attention check, 1 participant who indicated a household size of 0, and 1 participant who provided their age as the number of adults within the household. We had *N* = 464 after applying these criteria (*M*_age_ = 35.76, *SD*_age_ = 11.75; 45% women). This sample size gave 80% and 90% power to detect associations between PRD and external explanations of *r*s = 0.129 and 0.149, respectively (two‐tailed, *α* = 0.05).

#### 2.1.2 Procedure and Measures

Participants completed the following two measures in random order, with one measure per page:

##### 2.1.2.1 Personal Relative Deprivation Scale

Participants completed Callan, Shead, and Olson's (2011) 5‐item Personal Relative Deprivation Scale (PRDS; e.g., “I feel deprived when I think about what I have compared to what other people like me have”; “I feel privileged compared to other people like me”; two items were reverse scored). The measure was designed to gauge people's general beliefs and feelings associated with comparing their outcomes to the outcomes of their self‐selected peers. The PRDS has demonstrated good internal and temporal consistency (e.g., Callan, Kim, and Matthews 2015a) and convergent and discriminant validity (e.g., correlates with a tendency to make social comparisons of abilities but not of opinions; Callan, Kim, and Matthews 2015b). Participants responded to the items using a 6‐point scale (1 = *strongly disagree*, 6 = *strongly agree*). The average response was used as the index of PRD, with higher values indicating higher PRD (*α* = 0.87, *ω*_h_* = 0.64; here and at other points, we use an asterisk to indicate that the estimate of ω_h_ is problematic because the software produced warnings during the calculation—perhaps because the small number of items in the scale makes the factor‐analytic calculations difficult. We therefore report the values for information but suggest that any values flagged with an asterisk be treated with caution).

##### 2.1.2.2 Subjective Socioeconomic Status

Participants completed MacArthur's Scale of SSS (Adler et al. 2000). They were presented with an image of a 10‐rung ladder representing “where people stand in the United States,” with the top rung representing people who have the most money, highest education, and best jobs in the United States. Each participant clicked on the rung to indicate where they thought they stood relative to other people in the United States. Responses were coded 1–10, with higher scores indicating higher SSS.

Next, participants completed the following three measures in order, each on their own page:

##### 2.1.2.3 Sense of Personal Control

Participants completed Lachman and Weaver's (1998) 12‐item Sense of Control Scale. They rated how much they agreed with each item on a 7‐point scale (1 = *strongly disagree*; 7 = *strongly agree*). The scale measures the degree to which people believe they can control their outcomes (e.g., “Whether or not I am able to get what I want is in my own hands” and “What happens in my life is often beyond my control”). After reverse scoring relevant items, the average response was used as the index of a sense of personal control, with higher values indicating higher perceived personal control (*α* = 0.94, *ω*_*h*_ = 0.85).

##### 2.1.2.4 Explanations for Others' Positive and Negative Outcomes

Following Kraus, Piff, and Keltner (2009), we asked participants to provide ratings of internal versus external explanations for a series of positive and negative outcomes across a range of domains. The items were as following: *getting into medical school*, *having low income*, *receiving proper healthcare*, *contracting the HIV virus*, *failing a class at school*, *being obese*, *being laid‐off at work*, and *publishing a book*. Participants were asked to consider whether people experiencing these outcomes are “responsible for their own outcomes, or are the events caused by external forces outside of individual control?” and rated each of the eight outcomes on a 7‐point scale (1 = *individual primarily responsible*, 7 = *outside forces primarily responsible*). The average response was used as the index of one's orientation toward external explanations for social outcomes, with higher values indicating more externality (*α* = 0.76, *ω*_*h*_ = 0.53).

##### 2.1.2.5 Political Orientation

Participants rated their political orientation using a single item measure (“In terms of your political orientation, where would you place YOURSELF on this scale?”; 1 = *Very liberal*, 6 = *Very conservative*).

##### 2.1.2.6 Socio‐Demographics

As indicators of objective socioeconomic status, participants reported their annual household income, “What is your annual household income (before taxes)?”, with 16 response options (less than $10,000, $10,001 to $20,000, and up to greater than $150,000), number of adults and children living in the household, education level (did not finish high school, high school graduation, college graduation, postgraduate degree), gender, and age in years. For each study, the annual household income measure was re‐coded to a scale using category mid‐points, with the value for the unbounded top category being Parker and Fenwick's (1983) median‐based Pareto‐curve estimator (see Matthews, Gheorghiu, and Callan 2016). Annual household income was then divided by household size (*N*_adults_ + 0.5*N_children_; cf. Skylark and Baron‐Cohen 2017) for analysis.

### 2.2 Results

Descriptive statistics and the correlations among the focal measures and covariates are shown in Table S1 in the Supporting Information (hereafter tables and figures designated with the prefix S refer to those in the Supporting Information). As robustness checks, across Studies 1 to 5 we also computed Kendall's correlation matrix (for Studies 3 to 5, this robustness check was preregistered; the CIs were computed using the DescTools package version 0.99.42, Signorell et al. 2021). The pattern of results was very similar to the Pearson's correlations (see Tables S1 and S2).

To determine whether PRD predicts internal versus external explanations for others' outcomes controlling for the other variables, we regressed external explanations on PRD, SSS, household size‐adjusted income, education, political orientation, age, and gender (coded: women = 0.5, men = −0.5). For ease of interpretation, across studies all variables except for gender were standardized prior to analysis. However, the raw, unstandardized regression estimates are shown in Table S3. Table 1 shows that higher PRD and being more politically liberal were uniquely associated with externality; the 95% confidence interval for each of the remaining predictors included zero.

**TABLE 1:** Standardized regression coefficients from analyses across Studies 1 to 5.

Predictors	Study 1 (USA)	Study 2 (Asia)	Study 3 (USA)	Study 4 (UK)		Study 5 (USA)		
	Internal versus external explanations	Internal versus external explanations	Rotter's LoC	Wealthy external‐internal	Poverty external‐internal	External explanations	Internal explanations	External‐internal explanations
PRD	**0.21** (< 0.001)	**0.23** (< 0.001)	**0.25** (< 0.001)	**0.12** (0.007)	−0.08 (0.084)	**0.20** (< 0.001)	−0.06 (0.154)	**0.17** (< 0.001)
	[0.11, 0.31]	[0.16, 0.29]	[0.15, 0.35]	[0.03, 0.21]	[−0.17, 0.01]	[0.12, 0.29]	[−0.15, 0.02]	[0.09, 0.25]
SSS	−0.08 (0.170)	0.03 (0.136)	−0.04 (0.491)	−0.09 (0.076)	**−0.11** (0.033)	0.06 (0.154)	**0.27** (< 0.001)	**−0.12** (0.006)
	[−0.19, 0.03]	[−0.01, 0.07]	[−0.15, 0.07]	[−0.18, 0.01]	[−0.20, −0.01]	[−0.03, 0.15]	[0.18, 0.36]	[−0.21, −0.03]
Income	−0.09 (0.075)	—	0.01 (0.899)	0.04 (0.389)	0.03 (0.521)	**−0.18** (< 0.001)	−0.06 (0.195)	**−0.08** (0.048)
	[−0.18, 0.01]		[−0.09, 0.10]	[−0.05, 0.12]	[−0.06, 0.12]	[−0.27, −0.09]	[−0.14, 0.03]	[−0.17, −0.00]
Education	0.06 (0.164)	—	−0.03 (0.529)	**0.10** (0.023)	0.07 (0.108)	0.08 (0.061)	−0.00 (0.963)	0.05 (0.186)
	[−0.03, 0.15]		[−0.12, 0.06]	[0.01, 0.19]	[−0.02, 0.16]	[−0.00, 0.16]	[−0.08, 0.08]	[−0.03, 0.13]
Political	−**0.33** (< 0.001)	−0.01 (0.807)	**−0.18** (< 0.001)	**−0.50** (< 0.001)	**−0.54** (< 0.001)	**−0.29** (< 0.001)	**0.28** (< 0.001)	**−0.36** (< 0.001)
	[−0.41, −0.25]	[−0.09, 0.07]	[−0.27, −0.10]	[−0.59, −0.42]	[−0.62, −0.45]	[−0.37, −0.21]	[0.20, 0.36]	[−0.43, −0.28]
Age	0.00 (0.953)	0.00 (0.946)	**−0.24** (< 0.001)	0.01 (0.795)	−0.01 (0.757)	−0.01 (0.854)	**0.08** (0.043)	−0.05 (0.159)
	[−0.08, 0.09]	[−0.06, 0.07]	[−0.33, −0.15]	[−0.07, 0.09]	[−0.09, 0.07]	[−0.09, 0.07]	[0.00, 0.16]	[−0.13, 0.02]
Gender	−0.01 (0.884)	0.09 (0.153)	**0.29** (< 0.001)	−0.03 (0.730)	−0.01 (0.920)	0.15 (0.070)	**−0.21** (0.009)	**0.22** (0.004)
	[−0.09, 0.08]	[−0.03, 0.21]	[0.11, 0.46]	[−0.20, 0.14]	[−0.18, 0.16]	[−0.01, 0.31]	[−0.37, −0.05]	[0.07, 0.38]
*N*	464	3851	429	448	448	540	540	540
*R* ^*2*^ */R* ^*2*^_adj_	0.220/0.208	—	0.229/0.216	0.334/0.324	0.322/0.311	0.183/0.172	0.203/0.193	0.261/0.251

*Note:* Values in parentheses and brackets indicate *p* values and 95% confidence intervals, respectively. Bolded values show those estimates where the corresponding 95% CI does not include zero.

Abbreviations: LoC, locus of control; PRD, personal relative deprivation; SSS, subjective socioeconomic status.

We ran additional regression analyses to probe whether the results of our initial analysis were sensitive to different analytic decisions (cf. Skylark et al. 2020; these additional analyses were preregistered for Studies 3–5). Specifically, we re‐ran the regression using the robust regression function lmrob from the robustbase package version 0.93‐6 (with setting = “KS2014”; Maechler et al. 2021) and then repeated both the OLS and robust regression analyses using (a) log‐transformed income (in place of raw income; the logarithm was to base 10), and (b) coding education as a factor with weighted effect coding using the wec package 0.4‐1 (Grotenhuis et al. 2017). The pattern of results for these alternative regression analyses was largely similar to those shown in Table 1 (the full results are reported in the Table S4, which was made using sjPlot version 2.8.9; Lüdecke 2021). Noteworthy differences are that modeling log‐transformed income and/or using robust regression yielded 95% CIs that just excluded zero for income and education.

#### 2.2.1 Mediation Analysis

Following Kraus, Piff, and Keltner's (2009) research on subjective and objective social class and external explanations, we tested whether a reduced sense of personal control might account for the positive association between PRD and external explanations. Regressing sense of control on PRD, SSS, size‐adjusted household income, education, political orientation, age, and gender revealed a negative association between PRD and sense of personal control (a‐path), *B* = −0.64, *SE* = 0.04, 95% CI [−0.73, −0.55] (estimates for the remaining predictors are shown in Table S5). Regressing external explanations on a sense of personal control, PRD, SSS, household size‐adjusted income, education, political orientation, age, and gender revealed a negative association between a personal sense of control and external explanations (b‐path), *B* = −0.38, *SE* = 0.04, 95% CI [−0.45, −0.31]. We tested the indirect association between PRD and external explanations through a sense of personal control while controlling for the other predictors using the quasi‐Bayesian Monte Carlo method (5000 simulations) with the “mediation” package (version 4.5.0; Tingley et al. 2014). A sense of personal control mediated the relationship between PRD and external explanations (indirect association = 0.24, 95% Monte Carlo CI: 0.19, 0.30; *p* < 0.001). These results are consistent with the idea that the link between PRD and the belief that *others'* good and bad outcomes are caused more by external than internal factors is due to a reduced sense of *personal* control. That said, the direction of effect cannot be determined by our correlational design, nor can we rule out the influence of unmeasured confounding (see, e.g., Bullock, Green, and Ha 2010; Rohrer et al. 2022). Given these limitations, we focused on testing the relationship between PRD and either external versus internal explanations for self‐ or other‐relevant outcomes across our remaining studies.

## 3 Study 2

In Study 2, we tested the generalizability of the association between PRD and external explanations in a more culturally diverse sample of participants in 18 regions across Asia. Beyond the basic importance of seeking to establish more generalizable knowledge claims (e.g., Rad, Martingano, and Ginges 2018), investigating whether the relationship between PRD and locus of control we observed in Study 1 generalizes to other cultural contexts contributes to theory and research on potential cultural differences—or similarities—in the consequences of PRD.

Smith et al. (2018) found that the relationship between PRD and person‐level outcomes (e.g., life satisfaction) was stronger in more individualistic countries. They speculated that PRD predicts outcomes more strongly among people of more individualistic (vs. collectivistic) cultures because (a) their self‐worth tends to be more strongly tied to individual agentic (vs. communal) goal pursuits and (b) they react to comparative disadvantages more strongly. This suggests the possibility that the relationship between PRD and locus of control may be relatively muted in samples of participants from more collectivistic cultures.

That said, recent research has revealed similar associations between PRD and its putative antecedents and consequences among participants across Eastern and Western cultures. For example, Kim, Kim, et al. (2018) found that individual differences in the tendency to make social comparisons of abilities (Gibbons and Buunk 1999)—an important precursor to PRD—were just as strongly associated with individual differences in PRD in two Korean samples as in prior samples from the United States and United Kingdom (Callan, Kim, and Matthews 2015b; Kim, Callan, et al. 2018). Likewise, PRD has been found to be associated with various outcomes (e.g., worse self‐rated health) to a similar extent across samples from Eastern and Western cultures (e.g., Kim, Kim, et al. 2018). Kim, Kim, et al. (2018) noted that although people accustomed to more collectivistic cultures tend to place less importance on personal agency and self‐reliance, they might nonetheless experience PRD and the consequences thereof because they tend to make social comparisons more often (e.g., Guimond et al. 2007; White and Lehman 2005), with frequent social comparisons being important in more collectivistic, interdependent cultures to maintain group harmony, conform to group norms, and establish one's social standing within shared social networks (Sasaki, Ko, and Kim 2014). This work therefore suggests the possibility of observing a relationship between PRD and external explanations even among participants accustomed to more collectivistic cultures. Study 2 probed these possibilities.

### 3.1 Method

#### 3.1.1 Participants

The sample consisted of students in a variety of universities across 18 regions in Asia (mainland China, Hong Kong S. A. R., India, Japan, Macau S. A. R., South Korea, and Taiwan). From an initial *N* = 4453, we excluded responses from participants who had not been living in their corresponding regions since birth (*N* = 557) and those who did not answer all questions (*N* = 45). We had *N* = 3851 after applying these exclusion criteria (*M*_age_ = 21.11, *SD*_age_ = 3.83; 56% women). Table S6 shows the sample characteristics. Following local conventions, some participants were given course credit for their participation. This sample size gave 80% and 90% power to detect associations between PRD and external explanations of *r*s = 0.045 and 0.052, respectively (two‐tailed, *α* = 0.05).

#### 3.1.2 Procedure and Measures

Depending on local circumstances, participants completed the study either in class through pen‐and‐paper (3 regions) or online through a participant pool system (15 regions). Except for the surveys used in India and Hong Kong, all measures were translated from English to the target languages (see Table S6) which were then independently back‐translated.

Participants completed our focal measures within a larger survey on “worldviews and well‐being.” Specifically, participants completed the measure of SSS; they were informed the ladder represented where people stand in terms of money, education, and jobs “in your society.” Responses were coded 1–10, with higher scores indicating higher SSS. Participants then completed Kim, Kim, et al.'s (2018) 3‐item version of Callan, Shead, and Olson's (2011) PRDS (*α* = 0.82, *ω*_h_* = 0.02). Participants responded to the items using a 6‐point scale (1 = *strongly disagree*, 6 = *strongly agree*). Next, participants completed a modified version of the Kraus, Piff, and Keltner (2009) contextual explanations scale used in Study 1. The items were as follows: *getting into university, having low income, receiving proper healthcare, contracting an illness, being wealthy, being overweight, being unemployed, and getting divorced*. Participants rated each of the eight outcomes on a 7‐point scale (1 = *individual primarily responsible*, 7 = *outside forces primarily responsible*). The average response was used as the index of one's orientation toward external explanations for social outcomes, with higher values indicating more external explanations (*α* = 0.73, *ω*_*h*_ = 0.57). Finally, participants completed the same measure of political orientation used in Study (1 = *Very liberal*, 6 = *Very conservative*). We did not include measures of household income or educational attainment in Study 2.

### 3.2 Results

Table S7 and Figures S1 and S2 show the correlations among the focal measures and covariates across the entire sample and the correlations between each predictor and external explanations by region, respectively. Conceptually replicating Study 1, PRD was positively associated with external explanations, and this association was consistently positive across regions (Figure S1).

We fit a multilevel model using the lme4 package (version 1.1‐27, Bates et al. 2015) to test whether PRD predicts external explanations over and above the other predictors whilst accounting for nesting within data collection regions. The model included fixed effects for PRD, SSS, political orientation, age, and gender (coded: women = 0.5, men = −0.5, 0 = nonbinary/other), and random intercepts for site of data collection and random slopes by site for the associations with PRD, SSS, political orientation, age, and gender. Note that coding gender as a factor with weighted effect coding using the wec package 0.4‐1 (Grotenhuis et al. 2017) yielded virtually identical estimates for the partial associations between PRD and loci of control across studies.

Random effects were allowed to correlate (i.e., the model was “maximal”; Barr et al. 2013). We used Satterthwaite approximations to calculate *p* values and confidence intervals. As shown in Table 1, PRD was the only significant predictor of external explanations. As a robustness check, we refit the model without correlated random effects because the software flagged the fitted model as being singular. The fixed effect estimates from this simpler model were very similar to those from the “maximal” model (see Table S8). Additionally, given that participants were from data collection regions within larger territories, we fit a “maximal” three‐level model with data collection regions nested within the seven higher‐level territory groupings. The results revealed that the pattern of fixed effects is the same as that when we modeled only random effects by region (see Table S8).

## 4 Study 3

In Study 2, we found a positive association between PRD and external explanations in samples of university students across several regions in Asia, offering evidence of cross‐cultural generalizability. In Studies 3 and 4, we sought to generalize the findings of Studies 1 and 2 to different domain‐general and domain‐specific operationalizations of locus of control. Specifically, in Study 3, we used Rotter's (1966) internal‐external locus of control scale which measures internal versus external explanations across a range of domains (e.g., interpersonal success and academic achievement). In Study 4, we asked participants to rate internal and external explanations for why the wealthy are rich and the poor are living in poverty. Finding similar associations between PRD and greater externalizing across multiple operationalizations would bolster confidence that the associations we observed in Studies 1 and 2 are not limited to the specific measure we used.

### 4.1 Method

#### 4.1.1 Participants

Participants were recruited via Prolific.co. As per our preregistered sampling plan (aspredicted.org/RWF_EHV), we requested 450 participants from the United States. We had *N* = 429 after applying the preregistered exclusion criteria (*M*_age_ = 35.54, *SD*_age_ = 13.53; 54% women). This sample size gave 80% and 90% power to detect associations between PRD and external explanations of *r*s = 0.134 and 0.155, respectively (two‐tailed, *α* = 0.05).

#### 4.1.2 Procedure and Measures

The procedure and measures for Study 3 were similar to those for Study 1. Specifically, participants completed the PRDS (*α* = 0.84; *ω*_h_* = 0.59) and measure of SSS in a random order. Next, they completed Rotter's (1966) 23‐item Internal‐External Locus of Control Scale. For each item, participants chose one of two statements they agreed with the most. Each pair of statements included an internal (e.g., “People's misfortunes result from the mistakes they make”) versus external interpretation (e.g., “Many of the unhappy things in people's lives are partly due to bad luck”). The scale was designed to measure internal versus external locus of control across a range of domains, including academic achievement, interpersonal success, political influence, and general outlook. The sum of the number of external explanations chosen was used as the index of external locus of control, with higher values indicating a stronger external locus of control (*α* = 0.79, *ω*_*h*_ = 0.47). Finally, participants reported their household income, educational attainment, household size, political orientation, age, and gender.

### 4.2 Results

Table S9 shows descriptive statistics and the correlations among the measures (Table S10 shows the Kendall correlations). We regressed external locus of control on PRD, SSS, household size‐adjusted income, education, political orientation, age, and gender (coded: women = 0.5, men = −0.5, 0 = nonbinary/other). As shown in Table 1, higher PRD, being more politically liberal, being younger, and being woman were associated with an external locus of control. Our preregistered additional regression analyses (as per Study 1) yielded the same conclusions (see Table S11).

## 5 Study 4

### 5.1 Method

#### 5.1.1 Participants

Participants were recruited via Prolific.co. As per our preregistered sampling plan (aspredicted.org/LIJ_PCH), we requested 450 participants from the United Kingdom. We had *N* = 448 after applying our preregistered exclusion criteria (*M*_age_ = 36.57, *SD*_age_ = 14.05; 68% women). This sample size gave 80% and 90% power to detect associations between PRD and external explanations for being wealthy or being poor of *r*s = 0.131 and 0.152, respectively (two‐tailed, *α* = 0.05).

#### 5.1.2 Procedure and Measures

Participants completed the PRDS (*α* = 0.84; *ω*_*h*_ = 0.65) and ladder measure of SSS in a random order (the SSS measure asked participants to consider where they stood relative to other people in the United Kingdom). Next, they completed Davidai's (2018) measures of internal and external explanations for why the rich are wealthy and why the poor are in poverty. They were asked to indicate how important three internal factors (*personal drive, willingness to take risks; hard work and initiative*; and *ability and talent*; *α* = 0.89; *ω*_h_* = 0.01) and four external factors (*money inherited from family*, *political influence*, *good luck*, and *the British economic system allows them to take unfair advantage of the poor*; *α* = 0.74; *ω*_h_* = 0.03) were in explaining why the rich are wealthy. Participants also rated how important the corresponding internal factors (*lack of personal drive, willingness to take risks*; *lack of hard work and initiative*; and *lack of ability and talent; α* = 0.86; *ω*_h_* = 0.03) and external factors (*lack of money inherited from family*, *lack of political influence*, *bad luck*, and *the British economic system is stacked against the poor; α* = 0.72; *ω*_*h*_ = 0.70) were in explaining why the poor are in poverty. The measures of explanations for being wealthy and being poor were presented in a random order between participants, and participants rated the items using a 1 (*Not at all important*) to 7 (*Extremely important*) scale.

Following Davidai (2018) and our preregistered analysis plan, internal versus external explanations for being wealthy and being in poverty were scored by subtracting the mean importance for internal factors from the mean importance for external factors separately for being wealthy and being in poverty. Scores could thus range from −6 to 6, with higher scores indicating a greater importance placed on external factors.

Participants then reported their educational attainment with six options suited to the UK educational context: *No formal qualifications or equivalent* to *Doctoral degree or equivalent* (e.g., *PhD*). Annual household income was measured using 23 response options: £10,000 or less, £10,001–£15,000, £15,001–£20,000, and so on in £5000 steps until £80,001–£90,000 and so on in £10,000 steps until the top category, “More than £150,000.” Finally, participants indicated their household size, political orientation, age, and gender as per Study 3.

### 5.2 Results

Table S12 shows descriptive statistics and the correlations among the measures (Table S13 shows the Kendall's correlation matrix). We separately regressed external explanations for being wealthy and being in poverty on PRD, SSS, household size‐adjusted income, education, political orientation, age, and gender (coded: women = 0.5, men = −0.5, 0 = nonbinary/other). As shown in Table 1, higher PRD, being more politically liberal, and having more education were associated with external explanations for being wealthy. SSS and political orientation were the only predictors of external explanations for being in poverty. Our preregistered additional regression analyses (see Study 1 results) yielded the same conclusions (see Tables S14 and S15), except that the 95% CI for the negative relationship between PRD and external explanations for being in poverty just excluded zero for the robust regressions.

#### 5.2.1 Exploratory Analyses

Departing from our preregistered analysis plan, we explored whether the relationship between PRD and explanations might vary less by domain (i.e., being wealthy vs. being in poverty) than by type of explanation (i.e., internal vs. external). Here, rather than calculating the difference between external and internal explanations as above, we separately analyzed the average ratings of the internal and external explanations. Confirmatory factor analyses (CFA) using the lavaan package (version 0.6‐16, Rosseel 2012) revealed that a two‐factor solution for external explanations (for both being in poverty and being wealthy) and internal explanations as separate but correlated constructs showed significantly better fit than the one‐factor solution, *χ* ^2^_diff_ = 656.79, *df* = 1, *p* < 0.001.

We formed an overall index of internal explanations by averaging across the wealthy and poverty conditions (which were positively correlated, *r* = 0.55, *p* < 0.001, 95% CI [0.48, 0.61]) and likewise for external explanations (for which the correlation was *r* = 0.70, *p* < 0.001, 95% CI [0.65, 0.75]). PRD was positively associated with the overall measure of external explanations, *r* = 0.20, *p* < 0.001, 95% CI [0.11, 0.28], but not with the overall measure of internal explanations, *r* = −0.05, *p* = 0.32, 95% CI [−0.14, 0.05], pointing to the possibility that PRD might be more relevant for people's external than internal locus of control beliefs.

In Study 1, we tested whether people's perceptions of personal control mediate the relationship between PRD and explanations of others' outcomes using the *total* score from Lachman and Weaver's (1998) Sense of Control Scale (following Kraus, Piff, and Keltner 2009). Interestingly, Lachman and Weaver developed their scale to measure different but correlated facets of personal control: personal mastery (an individual's sense of how well they can control their circumstances, e.g., “What happens to me in the future mostly depends on me”; akin to Rotter's *internal* locus of control) and perceived constraints (the extent to which an individual believes there are external factors affecting their circumstances, e.g., “Other people determine most of what I can and cannot do”; akin to Rotter's *external* locus of control). Despite this, researchers tend to combine the personal mastery and perceived constraints items into a single measure (e.g., by reverse scoring the constraint items and averaging across all items, as we did in Study 1), effectively creating a bipolar measure of locus of control. However, a CFA of responses to the Sense of Control Scale from Study 1 revealed that a two‐factor solution with perceived constraints and personal mastery as separate but correlated factors showed significantly better fit than the one‐factor solution, *χ* ^2^_diff_ = 221.71, *df* = 1, *p* < 0.001. What's more, like the exploratory analyses above, regression analyses found that PRD was a statistically significant predictor of perceived constraints adjusting for the socio‐demographic variables and personal mastery, *β* = 0.35, 95% CI [0.28, 0.42], whereas PRD did not predict personal mastery while controlling for perceived constraints, *β* = −0.05, 95% CI [−0.13, 0.04] (see Table S16 for full model estimates).

These reanalyzes of Studies 1 and 4 should be viewed with caution given their exploratory nature, but they suggest that PRD might be less relevant to people's internal explanations than external explanations for their own and others' outcomes. We investigated this possibility across our remaining studies.

## 6 Study 5

In Study 5, we further probed the possibility that PRD is associated with an external locus of control more than an internal locus of control. Although researchers have typically treated the two loci of control as lying on a single continuum (e.g., by using Rotter's scale, which forces participants to choose between internal and external explanations), some evidence suggests that, when measured separately, internal and external loci of control are only modestly negatively correlated (e.g., Gatz and Good 1978; Gore, Griffin, and McNierney 2016; Hoffmann and Schenk 1988). For example, Gore et al. created separate measures of internal and external loci of control by asking participants to rate the individual statements contained within Rotter's (1966) internal‐external locus of control scale rather than using the original either‐or, forced‐choice response format. In three studies, they found that internal and external loci of control were only weakly to modestly correlated. These findings suggest that people can simultaneously hold seemingly contradictory beliefs about the internal and external causes of their own and others' good and bad outcomes, such as believing that people with low income are personally responsible for their financial situation while also recognizing the broader external influences at play.

This work points to the possibility that in a multidimensional assessment of locus of control (i.e., not operationalized along a bipolar, internal‐to‐external continuum as in our previous studies), PRD might be associated with an external but not internal locus of control. Such an association might due to people higher in PRD tending to weight external perceptions of control more than internal perceptions of control, given the tendency to self‐select targets for comparison who are already mutually sharing similar internal factors (see Kim, Callan et al. 2018).

### 6.1 Method

#### 6.1.1 Participants

Participants were recruited via Amazon's Mechanical Turk. As per our preregistered sampling plan (aspredicted.org/TMI_UDZ), we requested 600 participants from the United States and with an approval rating greater than or equal to 98% on the platform. We followed the same exclusion criteria as those used in Study 1. We had *N* = 540 after applying the preregistered exclusion criteria (*M*_age_ = 38.21, *SD*_age_ = 12.29; 40% women; two participants who provided their household income and age in years for the number of adults in their household were also excluded). This sample size gave 80% and 90% power to detect associations between PRD and internal and external explanations of *r*s = 0.120 and 0.138, respectively (two‐tailed, *α* = 0.05).

#### 6.1.2 Procedure and Measures

Participants completed the PRDS (*α* = 0.79; *ω*_*h*_ = 0.39) and US‐specific ladder measure of SSS in a random order. Next, they completed a modified version of the Kraus, Piff, and Keltner (2009) contextual explanations measure we used in Studies 1 and 2. Specifically, instead of using a bipolar, internal versus external explanations scale, participants in Study 5 rated each outcome (*getting into university, having low income, receiving proper healthcare, contracting an illness, being wealthy, being overweight, being unemployed, and getting divorced*) separately for internal and external explanations. For the internal explanations measure, participants were asked to rate “To what extent do you believe that, in general, each of these outcomes are determined by an individual's own actions or attributes?” using a 1 (*Not at all determined by an individual's own actions or attributes*) to 7 (*Strongly determined by an individual's own actions or attributes*) scale. The average response was used as an index of internal explanations, with higher values indicating stronger internal explanations (*α* = 0.80; *ω*_h_* = 0.60). For the external explanations measure, participants were asked to rate “To what extent do you believe that, in general, each of these outcomes are determined by external factors beyond an individual's own control?” using a 1 (*Not at all determined by external factors beyond an individual's control*) to 7 (*Strongly determined by external factors beyond an individual's control*) scale. The average response was used as the index of external explanations, with higher values indicating stronger external explanations (*α* = 0.82; *ω*_h_* = 0.70). Although not preregistered, confirmatory factor analyses (CFA) suggested that the two‐factor solution for explanations (i.e., with external and internal explanations as distinct but correlated factors) showed better fit than the one‐factor solution, *χ* ^2^_diff_ = 783.36, *df* = 1, *p* < 0.001.

For more direct comparisons with our previous studies and following our preregistration, we also computed an index of internal versus external explanations by subtracting the mean ratings for internal explanations from the mean ratings for external explanations (scores could range from −6 to 6, with higher scores indicating more external than internal explanations). The order in which participants completed the two measures of explanations was randomized between subjects. Finally, participants reported their household income, educational attainment, household size, political orientation, age, and gender as per Study 1.

### 6.2 Results

#### 6.2.1 Order Effect Analyses

As per our preregistered analysis plan, we first tested whether the order in which participants completed the measures of explanations affected their responses to these measures. A 2 (order: internal explanations measure first versus external explanations measure first) X 2 (measure: internal vs. external) ANOVA revealed a main effect of type of measure, *F*(1,538) = 38.08, *p* < 0.001, *ω* ^2^ = 0.04, such that participants explained the outcomes more strongly in terms of internal factors (*M* = 4.44, *SD* = 0.96) than external factors (*M* = 4.02, *SD* = 1.04), *dz* = 0.27, 95% CI [0.18, 0.35]. There was no main effect of order, *F*(1,538) = 0.43, *p* = 0.52, *ω* ^2^ = 0.00, nor an order X type of measure interaction, *F*(1,538) = 0.02, *p* = 0.89, *ω* ^2^ = 0.00. Given the lack of meaningful order effects, we did not model order in any subsequent analyses.

Table S17 shows descriptive statistics and correlations among the measures (Table S18 shows the Kendall's correlations). We separately regressed external explanations, internal explanations, and the difference between these measures on personal relative deprivation, SSS, household size‐adjusted income, education, political orientation, age, and gender (coded: women = 0.5, men = −0.5, 0 = nonbinary/other). As shown in Table 1, higher PRD was associated with external explanations but not internal explanations. Like our previous studies, higher PRD was associated with the index of external (vs. internal) explanations. Our preregistered additional regression analyses yielded the same pattern of results (see Tables S19–S21). Study 5 thus clarifies the results of Study 4 by demonstrating that PRD is associated with external more than internal explanations.

## 7 Study 6

Taken together, the findings from our first five studies suggest that PRD is positively associated with an external locus of control after controlling for other relevant variables that theoretically might confound this relationship. These findings, however, are correlational and do not provide direct insight into the social comparison contexts (i.e., unfavorable social comparisons of affluence with one's peers) we hypothesized to be important for an external locus of control to emerge from experiences of PRD. Teng et al. (2023) recently found that participants who were told that they had less (vs. the same) disposable income as people who shared a similar background reported a lower sense of personal control (as measured by Lachman and Weaver's 1998, scale; cf. Study 1). This work, however, concerned personal control beliefs and did not dissociate the effects of PRD on external versus internal explanations. In Study 6, we sought evidence for the idea that PRD causally affects external more than internal explanations for specific others' outcomes by experimentally inducing the types of social comparisons of affluence that people tend to make (i.e., with similar others; Gerber, Wheeler, and Suls 2018) and that are known to give rise to feelings of unfairness and resentment (Kim, Callan et al. 2018). Consistent with our theoretical perspective and the correlational evidence from our first five studies, in Study 6 we expected that making upward (vs. lateral) social comparisons of affluence with similar others would increase participants' endorsement of external explanations for a self‐selected target's financial standing to a greater extent than their endorsement of internal explanations.

### 7.1 Method

#### 7.1.1 Participants

Participants were recruited via Prolific.co; we requested 150 men and 150 women participants from the United Kingdom (aspredicted.org/TMI_UDZ). We had *N* = 271 after applying our preregistered exclusion criteria (*M*_age_ = 31.66, *SD*_age_ = 12.21; 50% women). This sample size gave 80% and 90% power to detect Comparison Direction X Type of Explanation interaction effects of *dz* = 0.171 and 0.198, respectively (two‐tailed, *α* = 0.05).

#### 7.1.2 Procedure and Measures

Study 6 used a fully within‐subjects design. Following Kim, Callan et al. (2018), we asked participants to identify two individuals they know who are similar to them in terms of their background qualifications and attributes (“e.g., the same abilities, educational or vocational qualifications, years of experience, skill set, motivation”) but who differ in their relative affluence: one who is better off financially than them and one who is just as well off financially as them. Participants were given a text box to provide the initials of their comparison target (e.g., RS), which were “piped” through to the questions that followed. Using this manipulation, Kim et al. (2018; see also Gheorghiu, Callan, and Skylark 2021) found that participants reported higher resentment and a greater sense of unfairness when thinking about a financially better off versus lateral target for comparison.

After identifying each comparison target, participants rated the extent to which they agreed that the target's relative financial situation could be explained by internal factors (five items: *better skills*, *more ability*, *more motivation*, *more intelligence*, and *a stronger work ethic*; *α*_better_ = 0.82, *ω*_*h*.better_ = 0.57, *α*_lateral_ = 0.85, *ω*_*h*.lateral_* = 0.69) and external factors (five items: *had better luck*, *often been at the right place at the right time*, *had better opportunities because of their family background or upbringing*, *had financial assistance from family or friends*, and *been provided with better support or connections*; *α*_better_ = 0.85, *ω*_*h*.better_* = 0.65, *α*_lateral_ = 0.84, *ω*_*h*.lateral_* = 0.66). The items were developed by drawing on taxonomies of lay explanations of financial success (e.g., Forgas, Morris, and Furnham 1982) and were similar to those we used in Study 4. Participants responded to the items using a 7‐point scale (1 = *strongly disagree*, 7 = *strongly agree*). The average response was used as the index of external and internal explanations for a financially better off and lateral comparison target, with higher values indicating stronger explanations. Confirmatory factor analyses (CFA) suggested that the two‐factor solution for explanations for better off targets (i.e., with external and internal explanations for the better off target's outcomes as distinct but correlated factors) showed superior fit over the one‐factor solution, *χ* ^2^_diff_ = 517.39, *df* = 1, *p* < 0.001. Likewise, the two‐factor solution for explanations for lateral targets showed better fit than the one‐factor solution, *χ* ^2^_diff_ = 489.80, *df* = 1, *p* < 0.001.

Study 6 was preceded by a smaller, preliminary study designed to ascertain the suitability and reliability of the measures of internal and external explanations. The results of this preliminary study yielded the same conclusions as the current Study 6 and are reported in the Supporting Information (see Text S1 and Figure S4).

### 7.2 Results

**FIGURE 1:**
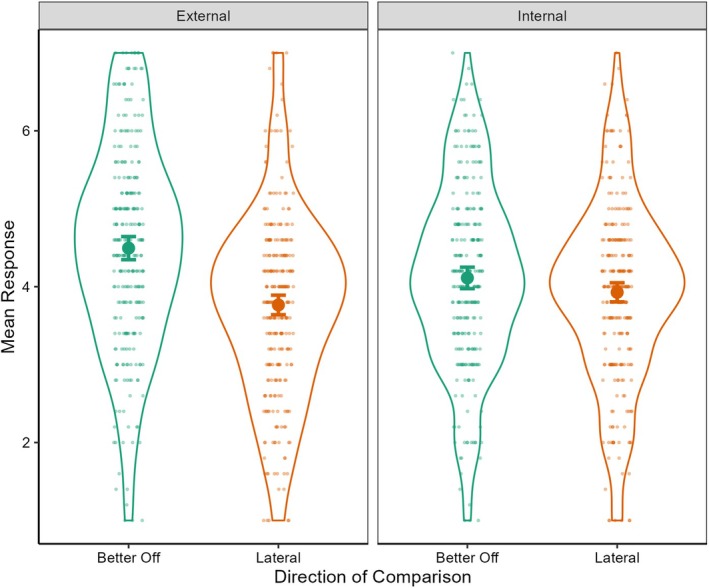
Effect of comparison direction as a function of type of explanation (Study 6). Raw data, descriptive and inferential statistics plot of participants' external and internal explanations as a function of making upward (identified target better off financially) and lateral (identified target just as well off financially) comparisons. The circles show mean explanations within conditions, and the error bars are within‐subject 95% CIs (Morey 2008).

A 2 (Comparison direction: better off vs. lateral target) × 2 (Type of explanation: internal vs. external) repeated‐measures ANOVA revealed a significant main effect of comparison direction, *F*(1, 270) = 69.51, *p* < 0.001, *dz* = 0.51, 95% CI [0.38, 0.63], but not type of explanation, *F*(1, 270) = 1.69, *p* = 0.20, *dz* = 0.08, 95% CI [−0.04, 0.20]. More importantly, there was a significant interaction effect, *F*(1, 270) = 20.24, *p* < 0.001, *dz* = 0.27, 95% CI [0.15, 0.39] (see Figure 1). Follow‐up paired‐samples *t*‐tests revealed that, as predicted, the effect of making a better off versus lateral social comparison of affluence on explanations was larger for external explanations, *t*(270) = 8.48, *p* < 0.001, *dz* = 0.52, 97.5% CI [0.37, 0.66], than for internal explanations *t*(270) = 2.39, *p* = 0.03, *dz* = 0.15, 97.5% CI [0.01, 0.28] (*p* values and CIs were Bonferroni corrected for two comparisons). These results provide support for the hypothesis that when making an upward (vs. lateral) social comparison of affluence with similar others, people favor external (vs. internal) explanations for their relative financial disadvantage.

## 8 Study 7

Our first five studies provided evidence for aggregated, between‐person associations among PRD and external (vs. internal) loci of control. However, associations observed at the between‐person level may not be evident—or can even change direction—at the within‐person level (see, e.g., Hamaker 2012). In Studies 7 and 8, we explored the reciprocal longitudinal associations (month‐to‐month and day‐to‐day, respectively) among PRD and external (perceived constraints) and internal (personal mastery) personal control beliefs. We adopted modeling strategies that disaggregated within‐person (i.e., state) and between‐person (i.e., trait) associations, allowing us to test whether PRD has consequences for personal control beliefs (and/or vice versa) that are realized at the trait level, “in the moment,” and prospectively over time.

### 8.1 Method

#### 8.1.1 Participants and Procedure

As per our preregistered sampling plan (aspredicted.org/7BW_5ZG), we requested 550 UK residents from Prolific.co at Time 1 (T1). We had *N* = 530 at T1 after applying our exclusion criteria (*M*_age_ = 42.91, *SD*_age_ = 12.79; 49% women). Participants received payments for each monthly survey they completed; a bonus payment was given to participants who completed all four surveys.

The same participants were contacted to complete identical questionnaires every 30 days for a total of four measurement occasions. After T1, the final sample sizes were *N*_T2_ = 464, *N*_T3_ = 460, and *N*_T4_ = 440. Participants completed our focal measures within a larger longitudinal study on neurodiversity and quality of life. Participants completed the measures in a randomized order at each timepoint. Socio‐demographic information was sought at the end of the T1 survey (age, sex as assigned at birth, education with 9 response options ranging from no education/qualifications to PhD, annual household income with 18 response options from less than £5000 to £85,001 and above, number of adults and children in the household, and political orientation as per Study 1).

#### 8.1.2 Measures

Participants completed Callan, Shead, and Olson's (2011) PRDS, the SSS ladder measure for a UK context, and the Perceived Constraints and Personal Mastery subscales of Lachman and Weaver's (1998) Sense of Control Scale at each timepoint.

### 8.2 Results

#### 8.2.1 Preliminary Analyses

We tested longitudinal measurement invariance in a CFA model including PRD, perceived constraints, and personal mastery as separate but correlated constructs using lavaan in R. All participants were included in analyses, and we used full information maximum likelihood estimation to handle missing data. We first tested a model where factor loadings were estimated freely over time (i.e., the configural model); we allowed item‐specific residual covariances over time across all models. The model fit was acceptable, *χ* ^2^(2038) = 4037.61, *p* < 0.001, CFI = 0.92, TLI = 0.92, RMSEA = 0.04, SRMR = 0.07. Next, we constrained factor loadings to be equal over time and compared this model to the configural model using a chi‐square difference test. The model with constrained factor loadings did not fit significantly worse than the configural model, Δ*χ* ^2^(42) = 27.55, *p* = 0.96, establishing metric invariance and permitting testing relationships among variables over time.

Descriptive statistics and correlations among variables at each timepoint are shown in Table S22. The PRD, Perceived Constraints, and Personal Mastery scales had Intraclass Correlation Coefficients (ICC) of 0.82, 0.84, and 0.74, respectively, suggesting limited intra‐individual variation over time (18%, 16%, and 26%, respectively).

#### 8.2.2 Random Intercept Cross‐Lagged Panel Model (RI‐CLPM)

Following our preregistered analysis plan, we fit a multiple indicator RI‐CLPM (Mulder and Hamaker 2021) to examine the prospective associations among PRD, perceived constraints, and personal mastery simultaneously. This means that estimated paths from PRD to control beliefs (and vice versa) reflect associations that persist after controlling for all other variables. The RI‐CLPM decomposes the observed variance into a stable, time‐invariant component (random intercepts) and a time varying within‐person component. The random intercepts represent a person's mean level of a given construct across time, and covariances among random intercepts indicate the extent to which trait‐like, between‐person differences between constructs are correlated across all timepoints. The within‐person part of the model includes factors that represent the time‐specific deviations of scores from an individual's average score (or trait level). Autoregressive parameters indicate the degree to which within‐person deviations from an individual's mean value at one timepoint are associated with further deviations at a later timepoint. The cross‐lagged parameters indicate the extent to which within‐person deviations from an individual's mean for a given variable at one timepoint (e.g., PRD at T1) are associated with deviations from an individual's mean for another variable at the next time point (e.g., Perceived Constraints at T2), controlling for previous within‐person deviations in each variable. Finally, the model also estimates the within‐person covariances among the variables at T1 and the occasion‐specific residual (or innovation) covariances for T2 onwards (i.e., the within‐person, occasion‐specific associations among the variables that cannot be explained by the autoregressive or cross‐lagged effects). We constrained the month‐to‐month autoregressive and cross‐lagged estimates to equality given the equivalent time lags (cf. John, Boileau, and Bless 2024). A graphical representation of the RI‐CLPM we fit to the data for Studies 7 and 8 is shown in Figure S3.

Shown in Table 2, none of the autoregressive paths were statistically significant. The only significant cross‐lagged path was from perceived constraints to personal mastery, suggesting that increases in perceived constraints predict subsequent decreases in personal mastery (but not vice versa). The pattern of within‐wave covariances and residual covariances are consistent with the results of our previous studies in terms of the dissociations between PRD and external versus internal control beliefs but at the *within‐person* level: participants lower‐than‐usual for PRD at T1 reported higher‐than‐usual perceived constraints (external locus of control) but not lower‐than‐usual personal mastery (internal locus of control). The same pattern was observed among the residual covariances across T2 to T4. At the between‐person, trait level, all the random intercepts were significantly correlated, such that higher relative deprivation was associated with higher perceived constraints and, to a lesser extent, lower personal mastery.

**TABLE 2:** RI‐CLPM unstandardized estimates for Studies 7 (month‐to‐month) and 8 (day‐to‐day).

	Study 7 (month‐to‐month)		Study 8 (day‐to‐day)	
	Estimate (SE)	95% CI	Estimate (SE)	95% CI
*Autoregressive paths*				
PRD → PRD	−0.12 (0.08)	[−0.27, 0.04]	**0.15** (0.02)	[0.10, 0.20]
PC → PC	0.07 (0.10)	[−0.12, 0.26]	**0.09** (0.03)	[0.03, 0.14]
PM → PM	0.00 (0.10)	[−0.20, 0.20]	**0.07** (0.03)	[0.02, 0.13]
*Cross‐lagged paths*				
PRD → PC	−0.07 (0.07)	[−0.22, 0.07]	0.06 (0.04)	[−0.02, 0.13]
PRD → PM	0.10 (0.09)	[−0.07, 0.28]	−0.03 (0.04)	[−0.10, 0.04]
PC → PRD	0.11 (0.07)	[−0.03, 0.26]	0.003 (0.02)	[−0.03, 0.04]
PM → PRD	0.11 (0.07)	[−0.02, 0.25]	−0.02 (0.02)	[−0.06, 0.01]
PC → PM	**−0.24** (0.10)	[−0.43, −0.05]	−0.05 (0.03)	[−0.10, 0.00]
PM → PC	−0.12 (0.08)	[−0.27, 0.03]	−0.05 (0.03)	[−0.10, 0.01]
*Covariances at T1*				
PRD ↔ PC	**0.05** (0.02)	[0.02, 0.09]	**0.18** (0.05)	[0.09, 0.27]
PRD ↔ PM	0.00 (0.02)	[−0.04, 0.03]	**−0.13** (0.04)	[−0.21, −0.04]
PC ↔ PM	**−0.09** (0.02)	[−0.14, −0.05]	**−0.18** (0.05)	[−0.28, −0.07]
*Residual covariances*				
PRD ↔ PC (T2)	**0.05** (0.02)	[0.01, 0.08]	**0.08–0.20**	(13/13)
PRD ↔ PC (T3)	**0.03** (0.02)	[0.00, 0.07]	—	—
PRD ↔ PC (T4)	**0.09** (0.02)	[0.06, 0.12]	—	—
PRD ↔ PM (T2)	−0.03 (0.02)	[−0.08, 0.01]	−0.03 to **−0.14**	(9/13)
PRD ↔ PM (T3)	0.03 (0.02)	[−0.01, 0.07]	—	—
PRD ↔ PM (T4)	**−0.05** (0.02)	[−0.09, −0.01]	—	—
PC ↔ PM (T2)	**−0.09** (0.02)	[−0.13, −0.04]	**−0.18 to −0.35**	(13/13)
PC ↔ PM (T3)	−0.04 (0.02)	[−0.08, 0.01]	—	—
PC ↔ PM (T4)	**−0.06** (0.02)	[−0.10, −0.02]	—	—
*Between‐person covariances*				
PRD ↔ PC (RI)	**0.80** (0.07)	[0.66, 0.95]	**0.35** (0.05)	[0.26, 0.45]
PRD ↔ PM (RI)	**−0.55** (0.06)	[−0.67, −0.43]	**−0.23** (0.04)	[−0.30, −0.15]
PC ↔ PM (RI)	**−0.87** (0.07)	[−1.02, −0.72]	**−0.26** (0.04)	[−0.33, −0.19]
*Model fit*				
CFI	0.93		0.93	
RMSEA	0.04		0.05	
SRMR	0.07		0.08	

*Note:* Paths/covariances shown in bold indicate estimates where the corresponding 95% CI does not contain zero. The range of estimates and whether their corresponding CIs exclude zero (/13) for the residual covariances across the 13 days are shown for Study 8 (a full table of these estimates and their corresponding CIs are shown in Table S24).

Abbreviations: PC, perceived constraints (external locus of control); PM, personal mastery (internal locus of control); PRD, personal relative deprivation; RI, random intercept; T, time point.

#### 8.2.3 Ancillary Analyses

For more direct comparison with our previous studies, we also separately regressed perceived constraints and personal mastery on PRD, SSS, household size‐adjusted income, education, political orientation, age, and sex (coded: women = 0.5, men = −0.5) using the T1 responses. Two additional regression models included the other locus of control variable as a covariate. Estimates are shown in Table S16. Of note, PRD was a statistically significant predictor of perceived constraints both with, *β* = 0.26, *SE* = 0.04, 95% CI [0.19, 0.34], and without, *β* = 0.42, *SE* = 0.04, 95% CI [0.34, 0.51], personal mastery as a covariate, whereas PRD did not predict personal mastery while controlling for perceived constraints, *β* = −0.05, *SE* = 0.04, 95% CI [−0.13, 0.03].

## 9 Study 8

One issue with Study 7 is that there was limited within‐person variability in PRD and loci of control across the 4 months (i.e., a relatively large portion of the variance was captured by the random intercepts), which increases the uncertainty of estimates at the within‐person level of the RI‐CLPM (see Mulder 2023). Interestingly, using the same (translated) measure, Guo and Xia (2023) found considerably more within‐person variability in PRD (44%) in a 3‐wave, 6‐month longitudinal study on cyberbullying than we observed in Study 7. One key difference between Guo and Xia's study and our Study 7 is that their sample was considerably younger on average (and were university students; *M*_age_ = 19 vs. 43, respectively). Arguably, one might expect younger adults to experience more variability in PRD over time because they have more diverse, dynamic, and changing social comparison contexts than older adults (e.g., larger and more complex social networks; Wrzus et al. 2013). Correspondingly, Callan, Kim, and Matthews (2015b) found that chronological age was negatively associated with social comparison tendency and PRD. In Study 8, then, we recruited a sample of younger adults (18–30 year olds) and probed the day‐to‐day within‐person associations among PRD, perceived constraints, and personal mastery across a 14‐day “daily diary” study.

### 9.1 Participants

We initially requested 200 participants living in the UK from Prolific.co for an intake survey (aspredicted.org/WP1_HDV) but slightly overrecruited (*N* = 205) owing to issues with replacing participants through the platform based on our exclusionary criteria. We had *N* = 196 participants who completed at least two daily diary surveys (*M*_age_ = 25.45, *SD*_age_ = 3.23; 46% women, 3% nonbinary/other). On average, participants completed 11.97 (*SD* = 3.33) of the 14 daily surveys (total daily surveys completed = 2744 or 85.53%).

### 9.2 Procedure

Participants completed our focal measures within a larger diary study involving measures of mindfulness, well‐being, and perceived values similarity. Socio‐demographic details were sought during an intake survey which occurred on a Sunday. The Monday following the intake survey, participants were invited to complete identical questionnaires every day for 14 consecutive days. The daily surveys were opened at 5:00 pm each day, and participants were given until 3:00 am to complete the survey. Any responses that were registered after 3:00 am were not included in analyses. Participants received payments for each daily survey they completed; a bonus payment was given to participants who completed at least 12 daily surveys.

### 9.3 Measures

Participants completed daily measures of PRD, perceived constraints, and personal mastery in a randomized order each day:

#### 9.3.1 PRD

We measured participants' daily PRD using a measure adapted from Callan et al. (2015). Within a larger instructional set to consider “how you felt about and responded to different social encounters that day,” participants indicated how dissatisfied, resentful, angry, and satisfied (reverse‐scored) they felt about their material and financial circumstances overall. Participants responded to the items using a 5‐point scale (1 = *not at all*, 5 = *very*). The average response was used as the index of daily PRD, with higher values indicating higher daily PRD. Callan et al. (2015) found that the main determinant of variance in participants' responses to these items was their perceived relative standing compared to similar others (vs. their household income, educational attainment, and SSS).

#### 9.3.2 Sense of Control Scale

We measured perceived constraints (“Today, I felt helpless in dealing with the problems of life,” “What happened to me today was often beyond my control”) and personal mastery (“Whether or not I was able to get what I wanted today was in my own hands,” “What happened to me today mostly depended on me”) using two items for each construct. These items were adapted from Lachman and Weaver's (1998) Sense of Control Scale. Participants responded to the items using a 5‐point scale (1 = *strongly disagree*, 5 = *strongly agree*). The average responses were computed for each measure by day, with higher values indicating higher perceived constraints and higher personal mastery.

### 9.4 Results

Descriptive statistics and reliability estimates for each measure by day are shown in Table S23. The PRD, Perceived Constraints, and Personal Mastery measures had ICCs of 0.71, 0.39, and 0.32, respectively, suggesting more within‐person variation for each measure (29%, 61%, and 68%, respectively) than we observed in Study 7.

Following our preregistration, we fit a RI‐CLPM to test the prospective associations among PRD, perceived constraints, and personal mastery simultaneously (using the average responses for each daily measure). Unlike Study 7, we decided a priori not to model latent factors (nor test for longitudinal measurement invariance) given the model complexity (42 latent factors, 3 per day) and the limited number of indicators for perceived constraints and personal mastery (2 each). However, consistent with our previous studies and past research (e.g., Infurna and Mayer 2015), CFAs revealed that a two‐factor solution with perceived constraints and personal mastery as separate but correlated factors showed significantly better fit than the one‐factor solution, *χ* ^2^_diff_ = 87.29, *df* = 12, *p* < 0.001, across the start, middle, and end of the study (Days 1, 8, and 14, respectively). Due to equally spaced intervals between days and for ease of interpretation and presentation, the autoregressive and cross‐lagged estimates were constrained to equality across time.

As shown in Table 2, all autoregressive paths were statistically significant, implying that days in which a participant scored higher than usual for PRD, perceived constraints, and personal mastery were followed by days in which they again scored above their average for these measures. No cross‐lagged paths were statistically significant. The pattern of within‐day, occasion‐specific covariances and residual covariances are consistent with that for Study 7: At the *within‐person* level, participants lower‐than‐usual for PRD reported higher‐than‐usual perceived constraints and, to a lesser degree, lower‐than‐usual personal mastery (Note that Table 2 shows ranges of estimates for the residual covariances across the 13 days; a full table of these estimates and their corresponding CIs are shown in Table S24). At the between‐person level, all the random intercepts were significantly correlated, such that higher relative deprivation was associated with higher perceived constraints and, to a lesser extent, lower personal mastery.

## 10 General Discussion

Across eight studies, we found support for the idea that higher PRD is associated with a more external locus of control. In Study 1, after accounting for the contributions of demographic and other status variables, participants higher in PRD more strongly endorsed external (vs. internal) explanations for a broad range of outcomes, and in Study 2, this pattern was replicated in a diverse sample of participants across several regions in Asia. Except for explanations for why people live in poverty, Studies 3 and 4 generalized these findings to different domain‐general and domain‐specific measures of locus of control. Study 5 probed the relative importance of PRD to locus of control by operationalizing external and internal explanations as separate constructs rather than falling along a single internal‐to‐external continuum. We found that PRD was more robustly associated with greater endorsement of external explanations than with weaker endorsement of internal explanations. In Study 6, we found causal evidence for our theoretical proposition that unfavorable (vs. neutral) social comparisons of affluence produce greater endorsement of external (vs. internal) explanations for a comparative target's relative standing.

Studies 7 and 8 revealed that although PRD did not predict next month or next day personal control beliefs (or vice versa), occasion‐specific, intra‐individual shifts in PRD were positively associated with intra‐individual shifts in perceived constraints. These findings clarify how the associations between PRD and loci of control are evident in the ongoing lives of individuals (i.e., at the within‐person level) and cannot be explained by time‐invariant confounders (e.g., gender, SES; see Rohrer and Murayama 2023) or preceding experiences of PRD or loci of control. However, the causal directions for the associations between PRD and loci of control within occasions cannot be determined from these studies given the multitude of internal and external events between measurement occasions that could have affected both. For example, a particularly potent unfavorable social comparison on a given day might heightened contemporaneous perceived constraints (but not necessarily next day or next month perceived constraints), an encounter with an overly controlling line manager might heighten same day resentment, or some common cause, such as stormy weather affecting one's mood on a given day, perturbs both PRD and perceived constraints away from their typical levels.

That said, our Study 6 results and those of Teng et al. (2023) suggest that in‐the‐moment unfavorable (vs. favorable) social comparisons of affluence do affect in‐the‐moment external explanations and a sense of personal control, respectively. This suggests that the unique within‐person covariation between PRD and external explanations we observed in Studies 7 and 8 might be explained by the momentary, situated nature of the judgments being made rather than varying day‐to‐day or month‐to‐month (cf. Smith and Semin 2007). This does not preclude longitudinal relationships over longer periods, but we provide evidence that PRD and external explanations are related because of momentary, labile socio‐cognitive processes. While acknowledging that the relationship between PRD and external locus of control may be bidirectional and/or partly attributable to third variables, our Study 6 findings point to PRD affecting external (but not internal) explanations in‐the‐moment. The challenge for future research will be to elucidate how the causal relationships between PRD and loci of control unfold dynamically within and across shorter (e.g., using ecological momentary assessment) and longer time intervals and contribute toward enduring, trait levels of these constructs.

Our multi‐method, multi‐measure approach provided important, converging evidence for the hypothesis that PRD is associated with adopting an external locus of control. However, because we focused on either self‐ or other‐relevant locus of control beliefs within each individual study (with the exception of Study 1), it is not clear whether, for example, the cross‐cultural generality of the Study 2 findings may be limited to the effects of PRD on explanations of *others'* outcomes, or whether the longitudinal, within‐person associations observed between PRD and *personal* control beliefs in Studies 7 and 8 occur for explanations of others' outcomes.

It will also be important for future research to probe the relationships between PRD and other attributional biases, such as the self‐serving bias, which involves attributing successes to one's own actions and abilities and failures to external factors (Mezulis et al. 2004). We found that people higher in PRD tended to attribute others' successes (e.g., being wealthy) and failures (e.g., being poor) to external factors but there are likely situations where individuals might be motivated to attribute their *own* relative financial advantages and disadvantages in more self‐serving ways. For example, in Study 6 we asked participants to provide ratings of the causes of their comparison target's relative affluence but not their own. Assessing self‐ *and* other‐relevant attributions in these contexts could yield interesting interplays between general perceptions of control and attributions that serve to protect one's self‐esteem.

### 10.1 Theoretical and Practical Contributions

The current research provides several important theoretical contributions to the literatures on PRD, locus of control, and the psychology of social class.

#### 10.1.1 Contributions to PRD Literature

Research into the consequences of PRD has mostly focused on emotional states (e.g., stress, depression) and/or individual behavior (e.g., deviance, gambling; see Smith, Pettigrew, and Huo 2020), with few studies attempting to elucidate the social‐cognitive processes through which Crosby (1976) hypothesized PRD is associated with these outcomes. We provide broadly consistent evidence that PRD is related to a general social‐cognitive tendency to favor external explanations for one's own and others' life events and outcomes. Given that an external locus of control has been identified as a proximal determinant of several of the same internal states and individual behaviors that are associated with PRD (e.g., problem gambling, von der Heiden and Egloff 2021; worse mental and physical well‐being, Lefcourt and Davidson‐Katz 1991; aggression, Zainuddin and Taluja 1990), the current studies point to the possibility that locus of control serves as a social‐cognitive mechanism linking these outcomes to PRD.

For example, PRD may negatively affect well‐being via an external locus of control orientation because adopting an external locus of control reduces one's propensity to engage in active, problem‐focused coping while increasing one's propensity to engage in passive, emotion‐focused coping. These coping styles, in turn, differentially elicit psychological distress (e.g., Anderson 1977). The relation between PRD and an external locus of control orientation may also help explain Callan et al.'s (2017) observation that participants higher in PRD tend to be less willing to act for the benefit of others (which requires a sense of personal agency and the belief that one's help‐giving will be effective, characteristic of an internal locus of control; e.g., Guagnano 1995) while at the same time wanting and expecting others to help them during times of need (which reflects assigning responsibility for one's outcomes to external factors). By evincing a link between PRD and a tendency to favor external explanations, the current studies provide a foundation for future theorizing and research exploring *why* PRD predicts a range of seemingly disparate attitudinal, behavioral, and well‐being outcomes, ranging from pro‐environmental intentions (Skylark and Callan 2021) to redistributive preferences (Brown‐Iannuzzi et al. 2015).

Beyond the potential for further theoretical development, considering how PRD may affect people's interpersonal relations and psychological functioning through the psychological mechanism of locus of control has practical applications for reducing its deleterious effects. These applications could include interventions aimed at re‐orienting locus of control by promoting personal agency (e.g., Tyler, Heffernan, and Fortune 2020), or promoting the adoption of adaptive coping strategies in the face of adverse social comparisons, such as active coping, acceptance, and positive reframing, all of which are associated with having a less externalized orientation and, consequently, positive outcomes for the individual (e.g., Aldao et al. 2010).

#### 10.1.2 Contributions to Locus of Control Literature

Locus of control is a widely known construct that has influenced a myriad of disciplines. Indeed, a Google Scholar search for “locus of control” reveals 22,300 results in 2023 alone. Despite the broad influence of the construct, relatively little work has explored the potential associations between PRD and locus of control beliefs. The present results, therefore, make an important contribution to this literature by providing correlational, experimental, and longitudinal evidence for a hitherto unexamined correlate of locus of control. The present results also make an important contribution to the literature by providing further evidence of the value of a bidimensional approach to the construct. They show that important nuances in the social‐psychological meaning of locus of control are revealed when internal and external loci of control are conceptualized as correlated but distinct constructs rather than as opposing ends of a single continuum (cf. Infurna and Mayer 2015; Gore, Griffin, and McNierney 2016; Gatz and Good 1978).

The present results also complement and qualify traditional theories that have emphasized social‐structural, largely immutable antecedents to locus of control beliefs, and research suggesting that lower SES is associated with an external locus of control (e.g., Carton and Nowicki 1996; Kraus, Piff, and Keltner 2009). In contrast, the present studies produced only mixed evidence for the hypothesis that SES is related to locus of control, and indeed, this relationship was rarely evident when we controlled for PRD and other demographic variables. It is worth noting that these weaker associations might have less to do with the predictive utility of SES for locus of control beliefs than the difficulty of appropriately conceiving and adequately measuring SES with the methodologies of psychology research (see Antonoplis 2023).

That said, the results of Studies 1 and 3 suggested that the relationship Kraus, Piff, and Keltner (2009) observed between subjective (but not objective) SES and internal versus external explanations is due to a confound: Participants higher in SES also tend to be lower in PRD, and PRD was the main “driver” of explanations in these studies (cf. Callan et al. 2015, 2017). But the results of Study 5 suggest that the predictive utility of SSS for these explanatory tendencies over and above covariation with PRD was obscured by using bipolar, internal‐to‐external measures of locus of control in these earlier studies. When internal *and* external explanations for the same set of outcomes were measured separately in Study 5, SSS accounted for unique variance in internal but not external explanations, whereas PRD accounted for unique variance in external but not internal explanations.

This finding illustrates how different components of social rank can have distinct (and complex) relationships with important social‐cognitive tendencies (cf. Callan et al. 2017). Central to Kraus et al.'s (2012) social‐cognitive theory of social class is the notion that, because of the social, material, and environmental hardships they face, people lower in SES adopt contextualist social‐cognitive tendencies characterized by the belief that one's outcomes are determined by external forces. Higher SES individuals, on the other hand, are hypothesized to adopt more solipsistic social‐cognitive tendencies characterized by the belief that outcomes are contingent on one's own personal characteristics (e.g., ability, motivation, and choices). Our data in Study 5 provide support for Kraus et al.'s theoretical proposition that SSS is associated with an *individualistic* orientation, such that, controlling for the other predictors, participants higher in SSS more strongly believed that various positive and negative outcomes are determined by an individual's own actions or attributes. But the same data suggest that people lower in SSS are no more contextualistic than people higher in SSS after accounting for the contributions of the other predictors. Thus, the unique contributions of SSS to explanatory tendencies are absent when internal‐external explanations are operationalized along a single continuum, but they pivot more around the internal dimension of locus of control when internal and external explanations are measured as separate constructs. The current results therefore point to the need for further research and theoretical development, especially since the proposition that people lower in SES are more sensitive to external forces and more inclined to explain events in external, situational terms has been used as a theoretical basis for research exploring, for example, lower class individuals' empathic accuracy (Kraus, Côté, and Keltner 2010), responses to chaos and randomness in the social environment (Piff et al. 2012), and compassion (Stellar et al. 2012).

Nonetheless, the present results provide important evidence for the conceptual framework we propose here, which places more emphasis on (relatively mutable) cognitive appraisals within the process of social comparison than on (relatively immutable) socio‐structural influences per se. Our results revealed that, by and large, PRD—whether measured as a stable individual difference, manipulated via social comparison, or tracked longitudinally—is a potent correlate of adopting external explanations over and above the contributions of SES.

## 11 General Conclusions

The overall picture to emerge from these studies is one in which PRD is positively associated with external locus of control, independent of subjective and objective socioeconomic status and political orientation, within‐ and between‐persons, and across cultures. Our findings demonstrate that PRD is associated with affirmation of external explanations more than rejection of internal explanations. This research suggests that resentment from people's beliefs that they are worse off than others who are similar to themselves has implications for their understanding of the causal forces that govern our lives. The challenge for future research will be to further elucidate the causal relationships among PRD and loci of control.

## Author Contributions

Conceptualization: Mitchell J. Callan. Design of methodology: Mitchell J. Callan, William J. Skylark, and Robbie M. Sutton. Project administration (Study 2): Phatthanakit Chobthamkit. Data curation: Mitchell J. Callan, Phatthanakit Chobthamkit, and Florence Y.N. Leung. Translation of measures: Hidefumi Hitokoto, Ding‐Yu Jiang, Eunkook M. Suh, and Michael Shengtao Wu. Data collection: Mitchell J. Callan, Phatthanakit Chobthamkit, Ryosuke Asano, Peter Beattie, Allan B.I. Bernardo, Chinun Boonroungrut, Jen‐Ho Chang, Anindita Chaudhuri, Chin‐Lung Chien, Hoon‐Seok Choi, Lixian Cui, Hongfei Du, Alexander Scott English, Kei Fuji, Junko Iida, Keiko Ishii, Ding‐Yu Jiang, Yashpal Jogdand, Hyejoo J. Lee, Florence Leung, Nobuhiro Mifune, Aya Murayama, Jinkyung Na, Kim One, Joonha Park, Kosuke Sato, Punit Shah, Suryodaya Sharma, Eunkook M. Suh, Arun Tipandjan, and Victoria Wai Lan Yeung. Statistical analysis and data visualization: Mitchell J. Callan and William J. Skylark. Writing – original draft: Mitchell J. Callan, William J. Skylark, and Robbie M. Sutton. Writing – review and editing: Mitchell J. Callan, Robbie M. Sutton, William J. Skylark, Victoria Wai Lan Yeung, and Florence Y.N. Leung.

## Conflicts of Interest

The authors declare no conflicts of interest.

## Supporting information


Data S1.

## Data Availability

All data, materials, and analysis syntax are available at https://osf.io/hfd5e/?view_only=cae7c5adf7624a539799e5d8958bf84d.

## References

[jopy12980-bib-0001] Adler, N. E. , E. S. Epel , G. Castellazzo , and J. R. Ickovics . 2000. “Relationship of Subjective and Objective Social Status With Psychological and Physiological Functioning: Preliminary Data in Healthy, White Women.” Health Psychology 19, no. 6: 586–592. 11129362 10.1037//0278-6133.19.6.586

[jopy12980-bib-0002] Aldao, A. , S. Nolen‐Hoeksema , and S. Schweizer . 2010. “Emotion‐Regulation Strategies Across Psychopathology: A Meta‐Analytic Review.” Clinical Psychology Review 30: 217–237. 20015584 10.1016/j.cpr.2009.11.004

[jopy12980-bib-0003] Anderson, C. R. 1977. “Locus of Control, Coping Behaviors, and Performance in a Stress Setting: A Longitudinal Study.” Journal of Applied Psychology 62, no. 4: 446–451. 885833

[jopy12980-bib-0004] Antonoplis, S. 2023. “Studying Socioeconomic Status: Conceptual Problems and an Alternative Path Forward.” Perspectives on Psychological Science 18: 275–292. 35981108 10.1177/17456916221093615 PMC10018062

[jopy12980-bib-0005] Barr, D. J. , R. Levy , C. Scheepers , and H. J. Tily . 2013. “Random Effects Structure for Confirmatory Hypothesis Testing: Keep it Maximal.” Journal of Memory and Language 68, no. 3: 255–278. 10.1016/j.jml.2012.11.001 PMC3881361 24403724

[jopy12980-bib-0006] Bates, D. , M. Maechler , B. Bolker , and S. G. Walker . 2015. “Fitting Linear Mixed‐Effects Models Using lme4.” Journal of Statistical Software 67: 1–48.

[jopy12980-bib-0007] Battle, E. S. , and J. B. Rotter . 1963. “Children's Feelings of Personal Control as Related to Social Class and Ethnic Group.” Journal of Personality 31, no. 4: 482–490. 14086840 10.1111/j.1467-6494.1963.tb01314.x

[jopy12980-bib-0008] Beshai, S. , S. Mishra , S. Mishra , and R. N. Carleton . 2017. “Personal Relative Deprivation Associated With Functional Disorders via Stress: An Examination of Fibromyalgia and Gastrointestinal Symptoms.” PLoS ONE 12, no. 12: e0189666. 29281686 10.1371/journal.pone.0189666 PMC5744949

[jopy12980-bib-0009] Brown‐Iannuzzi, J. L. , K. B. Lundberg , A. C. Kay , and B. K. Payne . 2015. “Subjective Status Shapes Political Preferences.” Psychological Science 26, no. 1: 15–26. 25416138 10.1177/0956797614553947

[jopy12980-bib-0010] Bullock, J. G. , D. P. Green , and S. E. Ha . 2010. “Yes, but what's the Mechanism? (Don't Expect an Easy Answer).” Journal of Personality and Social Psychology 98, no. 4: 550–558. 20307128 10.1037/a0018933

[jopy12980-bib-0011] Callan, M. J. , H. Kim , A. I. Gheorghiu , and W. J. Matthews . 2017. “The Interrelations Between Social Class, Personal Relative Deprivation, and Prosociality.” Social Psychological and Personality Science 8, no. 6: 660–669. 29081900 10.1177/1948550616673877 PMC5641987

[jopy12980-bib-0012] Callan, M. J. , H. Kim , and W. J. Matthews . 2015a. “Predicting Self‐Rated Mental and Physical Health: The Contributions of Subjective Socioeconomic Status and Personal Relative Deprivation.” Frontiers in Psychology 6: 1415. 26441786 10.3389/fpsyg.2015.01415 PMC4585190

[jopy12980-bib-0013] Callan, M. J. , H. Kim , and W. J. Matthews . 2015b. “Age Differences in Social Comparison Tendency and Personal Relative Deprivation.” Personality and Individual Differences 87: 196–199.

[jopy12980-bib-0014] Callan, M. J. , N. W. Shead , and J. M. Olson . 2011. “Personal Relative Deprivation, Delay Discounting, and Gambling.” Journal of Personality and Social Psychology 101: 955–973. 21875231 10.1037/a0024778

[jopy12980-bib-0015] Carton, J. S. , and S. Nowicki Jr. 1996. “Origins of Generalized Control Expectancies: Reported Child Stress and Observed Maternal Control and Warmth.” Journal of Social Psychology 136, no. 6: 753–760. 9043204 10.1080/00224545.1996.9712251

[jopy12980-bib-0017] Cheng, C. , M. W. L. Cheung , and B. C. Lo . 2016. “Relationship of Health Locus of Control With Specific Health Behaviours and Global Health Appraisal: A Meta‐Analysis and Effects of Moderators.” Health Psychology Review 10, no. 4: 460–477. 27556686 10.1080/17437199.2016.1219672 PMC5214986

[jopy12980-bib-0018] Crosby, F. 1976. “A Model of Egoistical Relative Deprivation.” Psychological Review 83: 85–113.

[jopy12980-bib-0019] Culpin, I. , L. Stapinski , Ö. B. Miles , R. Araya , and C. Joinson . 2015. “Exposure to Socioeconomic Adversity in Early Life and Risk of Depression at 18 Years: The Mediating Role of Locus of Control.” Journal of Affective Disorders 183: 269–278. 26047304 10.1016/j.jad.2015.05.030 PMC4504028

[jopy12980-bib-0020] Davidai, S. 2018. “Why Do Americans Believe in Economic Mobility? Economic Inequality, External Attributions of Wealth and Poverty, and the Belief in Economic Mobility.” Journal of Experimental Social Psychology 79: 138–148.

[jopy12980-bib-0021] Festinger, L. 1954. “A Theory of Social Comparison Processes.” Human Relations 7: 117–140.

[jopy12980-bib-0022] Findley, M. J. , and H. M. Cooper . 1983. “Locus of Control and Academic Achievement: A Literature Review.” Journal of Personality and Social Psychology 44, no. 2: 419–427.

[jopy12980-bib-0023] Forgas, J. P. , S. L. Morris , and A. Furnham . 1982. “Lay Explanations of Wealth: Attributions for Economic Success.” Journal of Applied Social Psychology 12, no. 5: 381–397.

[jopy12980-bib-0024] Galvin, B. M. , A. E. Randel , B. J. Collins , and R. E. Johnson . 2018. “Changing the Focus of Locus (of Control): A Targeted Review of the Locus of Control Literature and Agenda for Future Research.” Journal of Organizational Behavior 39, no. 7: 820–833.

[jopy12980-bib-0025] Gatz, M. , and P. R. Good . 1978. “An Analysis of the Effects of the Forced‐Choice Format of rotter's Internal‐External Scale.” Journal of Clinical Psychology 34, no. 2: 381–385.

[jopy12980-bib-0026] Gerber, J. P. , L. Wheeler , and J. Suls . 2018. “A Social Comparison Theory Meta‐Analysis 60+ Years on.” Psychological Bulletin 144, no. 2: 177–197. 29144145 10.1037/bul0000127

[jopy12980-bib-0027] Gheorghiu, A. I. , M. J. Callan , and W. J. Skylark . 2021. “Having Less, Giving Less: The Effects of Unfavorable Social Comparisons of Affluence on people's Willingness to Act for the Benefit of Others.” Journal of Applied Social Psychology 51: 946–961.

[jopy12980-bib-0028] Gibbons, F. X. , and B. P. Buunk . 1999. “Individual Differences in Social Comparison: Development of a Scale of Social Comparison Orientation.” Journal of Personality and Social Psychology 76: 129–142. 9972558 10.1037//0022-3514.76.1.129

[jopy12980-bib-0029] Gore, J. S. , D. P. Griffin , and D. McNierney . 2016. “Does Internal or External Locus of Control Have a Stronger Link to Mental and Physical Health?” Psychological Studies 61, no. 3: 181–196.

[jopy12980-bib-0030] Greitemeyer, T. , and C. Sagioglou . 2019. “The Impact of Personal Relative Deprivation on Aggression Over Time.” Journal of Social Psychology 159, no. 6: 664–675. 30541413 10.1080/00224545.2018.1549013 PMC6816473

[jopy12980-bib-0031] Grotenhuis, M. t. , B. Pelzer , R. Eisinga , R. Nieuwenhuis , A. Schmidt‐Catran , and R. Konig . 2017. “When Size Matters: Advantages of Weighted Effect Coding in Observational Studies.” International Journal of Public Health 62, no. 1: 163–167. 27796415 10.1007/s00038-016-0901-1 PMC5288425

[jopy12980-bib-0032] Guagnano, G. A. 1995. “Locus of Control, Altruism and Agentic Disposition.” Population and Environment 17, no. 1: 63–77.

[jopy12980-bib-0033] Guimond, S. , N. R. Branscombe , S. Brunot , et al. 2007. “Culture, Gender, and the Self: Variations and Impact of Social Comparison Processes.” Journal of Personality and Social Psychology 92, no. 6: 1118–1134. 17547492 10.1037/0022-3514.92.6.1118

[jopy12980-bib-0034] Guo, Y. , and L. X. Xia . 2023. “Relational Model of Relative Deprivation, Revenge, and Cyberbullying: A Three‐Time Longitudinal Study.” Aggressive Behavior 49, no. 4: 333–344. 10.1002/ab.22079. 36842166

[jopy12980-bib-0035] Hamaker, E. L. 2012. “Why Researchers Should Think “Within‐Person”: A Paradigmatic Rationale.” In Handbook of Research Methods for Studying Daily Life, edited by M. R. Mehl and T. S. Conner , 43–61. New York, NY: Guilford Press.

[jopy12980-bib-0036] Hoffmann, M. , and J. Schenk . 1988. “Bipolar Locus‐of‐Control Scales.” Personality and Individual Differences 9, no. 4: 839–841.

[jopy12980-bib-0097] Infurna, F. J. , and A. Mayer . 2015. “The Effects of Constraints and Mastery on Mental and Physical Health: Conceptual and Methodological Considerations.” Psychology and Aging 30, no. 2: 432–448. 10.1037/a0039050. 25938243 PMC4451433

[jopy12980-bib-0037] John, M. , L. L. A. Boileau , and H. Bless . 2024. “Effect of Social Class on Personal Control Beliefs.” Journal of Personality 92, no. 4: 1086–1099. 37602944 10.1111/jopy.12872

[jopy12980-bib-0038] Johnson, R. E. , C. C. Rosen , C.‐H. D. Chang , and S.‐H. J. Lin . 2015. “Getting to the Core of Locus of Control: Is It an Evaluation of the Self or the Environment?” Journal of Applied Psychology 100, no. 5: 1568–1578. 25664470 10.1037/apl0000011

[jopy12980-bib-0039] Kim, H. , M. J. Callan , A. I. Gheorghiu , and W. J. Matthews . 2017. “Social Comparison, Personal Relative Deprivation, and Materialism.” British Journal of Social Psychology 56, no. 2: 373–392. 27878836 10.1111/bjso.12176 PMC5484278

[jopy12980-bib-0040] Kim, H. , M. J. Callan , A. I. Gheorghiu , and W. J. Skylark . 2018. “Social Comparison Processes in the Experience of Personal Relative Deprivation.” Journal of Applied Social Psychology 48, no. 9: 519–532.

[jopy12980-bib-0041] Kim, H. , E. Kim , E. M. Suh , and M. J. Callan . 2018. “Development and Preliminary Validation of a Korean Version of the Personal Relative Deprivation Scale.” PLoS One 13, no. 5: e0197279. 29746534 10.1371/journal.pone.0197279 PMC5945005

[jopy12980-bib-0042] Kraus, M. W. , S. Côté , and D. Keltner . 2010. “Social Class, Contextualism, and Empathic Accuracy.” Psychological Science 21, no. 11: 1716–1723. 20974714 10.1177/0956797610387613

[jopy12980-bib-0043] Kraus, M. W. , P. K. Piff , and D. Keltner . 2009. “Social Class, Sense of Control, and Social Explanation.” Journal of Personality and Social Psychology 97, no. 6: 992–1004. 19968415 10.1037/a0016357

[jopy12980-bib-0044] Kraus, M. W. , P. K. Piff , R. Mendoza‐Denton , M. L. Rheinschmidt , and D. Keltner . 2012. “Social Class, Solipsism, and Contextualism: How the Rich Are Different From the Poor.” Psychological Review 119, no. 3: 546–572. 22775498 10.1037/a0028756

[jopy12980-bib-0045] Lachman, M. E. , and S. L. Weaver . 1998. “The Sense of Control as a Moderator of Social Class Differences in Health and Well‐Being.” Journal of Personality and Social Psychology 74, no. 3: 763–773. 9523418 10.1037//0022-3514.74.3.763

[jopy12980-bib-0046] Lefcourt, H. M. 1982. Locus of Control: Current Trends in Theory & Research. 2nd ed. New York, NY: Psychology Press.

[jopy12980-bib-0047] Lefcourt, H. M. , and K. Davidson‐Katz . 1991. “Locus of Control and Health.” In Handbook of Social and Clinical Psychology: The Health Perspective, edited by C. R. Snyder and D. R. Forsyth , 246–266. Elmsford, NY: Pergamon Press.

[jopy12980-bib-0048] Lüdecke, D. 2021. sjPlot: Data Visualization for Statistics in Social Science R Package Version 2.8.9.

[jopy12980-bib-0049] Maechler, M. , P. Rousseeuw , C. Croux , et al. 2021. “Robustbase: Basic Robust Statistics.” R Package Version 0.93‐6.

[jopy12980-bib-0050] Manstead, A. S. , M. J. Easterbrook , and T. Kuppens . 2020. “The Socioecology of Social Class.” Current Opinion in Psychology 32: 95–99. 31419784 10.1016/j.copsyc.2019.06.037

[jopy12980-bib-0051] Marks, G. , and N. Miller . 1987. “Ten Years of Research on the False‐Consensus Effect: An Empirical and Theoretical Review.” Psychological Bulletin 102, no. 1: 72–90.

[jopy12980-bib-0052] Matthews, W. J. , A. I. Gheorghiu , and M. J. Callan . 2016. “Why Do We Overestimate others' Willingness to Pay?” Judgment and Decision Making 11: 21–39.

[jopy12980-bib-0053] Mezulis, A. H. , L. Y. Abramson , J. S. Hyde , and B. L. Hankin . 2004. “Is There a Universal Positivity Bias in Attributions? A Meta‐Analytic Review of Individual, Developmental, and Cultural Differences in the Self‐Serving Attributional Bias.” Psychological Bulletin 130: 711–747. 15367078 10.1037/0033-2909.130.5.711

[jopy12980-bib-0098] Mirowsky, J. , and C. E. Ross . 1990. “Control or Defense? Depression and the Sense of Control over Good and Bad Outcomes.” Journal of Health and Social Behavior 31: 71–86. 2313078

[jopy12980-bib-0054] Morey, R. D. 2008. “Confidence Intervals From Normalized Data: A Correction to Cousineau (2005).” Tutorials in Quantitative Methods for Psychology 4, no. 2: 61–64.

[jopy12980-bib-0055] Mulder, J. D. 2023. “Power Analysis for the Random Intercept Cross‐Lagged Panel Model Using the powRICLPM R‐Package.” Structural Equation Modeling: A Multidisciplinary Journal 30, no. 4: 645–658. 10.31234/osf.io/wktrb PMC7615284 37937063

[jopy12980-bib-0056] Mulder, J. D. , and E. L. Hamaker . 2021. “Three Extensions of the Random Intercept Cross‐Lagged Panel Model.” Structural Equation Modeling: A Multidisciplinary Journal 28, no. 4: 638–648.

[jopy12980-bib-0057] Mullen, B. , J. L. Atkins , D. S. Champion , et al. 1985. “The False Consensus Effect: A Meta‐Analysis of 115 Hypothesis Tests.” Journal of Experimental Social Psychology 21, no. 3: 262–283.

[jopy12980-bib-0058] Nowicki, S. 1978. “Reported Stressful Events During Developmental Periods and Their Relation to Locus of Control Orientation in College Students.” Journal of Consulting and Clinical Psychology 46, no. 6: 1552–1553.

[jopy12980-bib-0059] Nowicki, S. , and M. P. Duke . 2016. “Foundations of Locus of Control Research.” In Perceived Control: Theory, Research, and Practice in the First 50 Years, edited by F. Infurna and J. W. Reich . New York: Oxford University Press.

[jopy12980-bib-0060] Nowicki, S. , G. Ellis , Y. Iles‐Caven , S. Gregory , and J. Golding . 2018. “Events Associated With Stability and Change in Adult Locus of Control Orientation Over a Six‐Year Period.” Personality and Individual Differences 126: 85–92. 29725146 10.1016/j.paid.2018.01.017 PMC5818169

[jopy12980-bib-0061] Parker, R. N. , and R. Fenwick . 1983. “The Pareto Curve and Its Utility for Open‐Ended Income Distributions in Survey Research.” Social Forces 61, no. 3: 872–885.

[jopy12980-bib-0062] Piff, P. K. , D. M. Stancato , A. G. Martinez , M. W. Kraus , and D. Keltner . 2012. “Class, Chaos, and the Construction of Community.” Journal of Personality and Social Psychology 103, no. 6: 949–962. 22889070 10.1037/a0029673

[jopy12980-bib-0063] Powell, A. , and M. Vega . 1972. “Correlates of Adult Locus of Control.” Psychological Reports 30, no. 2: 455–460. 5024923 10.2466/pr0.1972.30.2.455

[jopy12980-bib-0064] Presson, P. K. , and V. A. Benassi . 1996. “Locus of Control Orientation and Depressive Symptomatology: A Meta‐Analysis.” Journal of Social Behavior and Personality 11, no. 1: 201–212.

[jopy12980-bib-0065] R Core Team . 2023. A Language and Environment for Statistical Computing. Vienna, Austria: R Foundation for Statistical Computing. https://www.R‐project.org.

[jopy12980-bib-0066] Rad, M. S. , A. J. Martingano , and J. Ginges . 2018. “Toward a Psychology of Homo Sapiens: Making Psychological Science More Representative of the Human Population.” Proceedings of the National Academy of Sciences 115, no. 45: 11401–11405. 10.1073/pnas.1721165115 PMC6233089 30397114

[jopy12980-bib-0067] Rohrer, J. M. , P. Hünermund , R. C. Arslan , and M. Elson . 2022. “That's a Lot to Process! Pitfalls of Popular Path Models.” Advances in Methods and Practices in Psychological Science 5, no. 2: 25152459221095827.

[jopy12980-bib-0068] Rohrer, J. M. , and K. Murayama . 2023. “These Are Not the Effects You Are Looking for: Causality and the Within‐/Between‐Persons Distinction in Longitudinal Data Analysis.” Advances in Methods and Practices in Psychological Science 6, no. 1: 25152459221140842.

[jopy12980-bib-0069] Ross, C. E. , and J. Mirowsky . 1989. “Explaining the Social Patterns of Depression: Control and Problem Solving—Or Support and Talking?” Journal of Health and Social Behavior 30: 206–219. 2738367

[jopy12980-bib-0070] Ross, L. , D. Greene , and P. House . 1977. “The “False Consensus Effect”: An Egocentric Bias in Social Perception and Attribution Processes.” Journal of Experimental Social Psychology 13, no. 3: 279–301.

[jopy12980-bib-0071] Rosseel, Y. 2012. “Lavaan: An R Package for Structural Equation Modeling.” Journal of Statistical Software 48, no. 2: 1–36. https://www.jstatsoft.org/v48/i02/.

[jopy12980-bib-0072] Rotter, J. B. 1954. Social Learning and Clinical Psychology. New York: Prentice‐Hall.

[jopy12980-bib-0073] Rotter, J. B. 1966. “Generalized Expectancies for Internal Versus External Control of Reinforcement.” Psychological Monographs: General and Applied 80, no. 1: 1–28. 5340840

[jopy12980-bib-0074] Sasaki, J. Y. , D. M. Ko , and H. S. Kim . 2014. “Culture and Self‐Worth: Implications for Social Comparison Processes and Coping With Threats to Self‐Worth.” In Communal Functions of Social Comparison, edited by Z. Krizan and F. X. Gibbons , 230–252. Cambridge: Cambridge University Press.

[jopy12980-bib-0075] Signorell, A. , K. Aho , A. Alfons , N. Anderegg , T. Aragon , and A. Arppe . 2021. DescTools: Tools for Descriptive Statistics R Package Version 0.99.42.

[jopy12980-bib-0076] Skylark, W. J. , and S. Baron‐Cohen . 2017. “Initial Evidence That Non‐Clinical Autistic Traits are Associated With Lower Income.” Molecular Autism 8: 61. 29158888 10.1186/s13229-017-0179-z PMC5683395

[jopy12980-bib-0077] Skylark, W. J. , and M. J. Callan . 2021. “Personal Relative Deprivation and Pro‐Environmental Intentions.” PLoS ONE 16, no. 11: e0259711. 34793509 10.1371/journal.pone.0259711 PMC8601418

[jopy12980-bib-0078] Skylark, W. J. , K. T. F. Chan , G. D. Farmer , K. W. Gaskin , and A. R. Miller . 2020. “The Delay‐Reward Heuristic: What Do People Expect in Intertemporal Choice Tasks?” Judgment and Decision Making 15, no. 5: 611–629. 33082904 PMC7116214

[jopy12980-bib-0079] Smith, E. R. , and G. R. Semin . 2007. “Situated social cognition.” Current Directions in Psychological Science 16, no. 3: 132–135.

[jopy12980-bib-0080] Smith, H. J. , T. F. Pettigrew , and Y. J. Huo . 2020. “Relative Deprivation Theory: Advances and Applications.” In Social Comparison, Judgment, and Behavior, edited by J. Suls , R. L. Collins , and L. Wheeler , 495–526. Oxford, UK: Oxford University Press.

[jopy12980-bib-0081] Smith, H. J. , T. F. Pettigrew , G. M. Pippin , and S. Bialosiewicz . 2012. “Relative Deprivation: A Theoretical and Meta‐Analytic Review.” Personality and Social Psychology Review 16: 203–232. 22194251 10.1177/1088868311430825

[jopy12980-bib-0082] Smith, H. J. , D. A. Ryan , A. Jaurique , et al. 2018. “Cultural Values Moderate the Impact of Relative Deprivation.” Journal of Cross‐Cultural Psychology 49, no. 8: 1183–1218.

[jopy12980-bib-0083] Stellar, J. E. , V. M. Manzo , M. W. Kraus , and D. Keltner . 2012. “Class and Compassion: Socioeconomic Factors Predict Responses to Suffering.” Emotion 12, no. 3: 449–459. 22148992 10.1037/a0026508

[jopy12980-bib-0084] Stouffer, S. A. , A. A. Lumsdaine , M. H. Lumsdaine , et al. 1949. The American Soldier: Combat and Its Aftermath. Vol. 11. Princeton, NJ: Princeton University Press.

[jopy12980-bib-0085] Teng, F. , X. Wang , Y. A. Li , Y. Zhang , and Q. Lei . 2023. “Personal Relative Deprivation Increases Men's (But Not Women's) Hostile Sexism: The Mediating Role of Sense of Control.” Psychology of Women Quarterly 47, no. 2: 231–249.

[jopy12980-bib-0086] Tingley, D. , T. Yamamoto , K. Hirose , L. Keele , and K. Imai . 2014. “Mediation: R Package for Causal Mediation Analysis.” Journal of Statistical Software 59, no. 5: 1–38.26917999

[jopy12980-bib-0087] Tougas, F. , N. Rinfret , A. M. Beaton , and R. de la Sablonnière . 2005. “Policewomen Acting in Self‐Defense: Can Psychological Disengagement Protect Self‐Esteem From the Negative Outcomes of Relative Deprivation?” Journal of Personality and Social Psychology 88, no. 5: 790–800. 15898875 10.1037/0022-3514.88.5.790

[jopy12980-bib-0088] Tyler, N. , R. Heffernan , and C. A. Fortune . 2020. “Reorienting Locus of Control in Individuals Who Have Offended Through Strengths‐Based Interventions: Personal Agency and the Good Lives Model.” Frontiers in Psychology 11. 10.3389/fpsyg.2020.553240. PMC7522323 33041920

[jopy12980-bib-0089] von der Heiden, J. M. , and B. Egloff . 2021. “Associations of the Big Five and Locus of Control With Problem Gambling in a Large Australian Sample.” PLoS ONE 16, no. 6: e0253046. 34125840 10.1371/journal.pone.0253046 PMC8202919

[jopy12980-bib-0090] Walker, L. , and L. Mann . 1987. “Unemployment, Relative Deprivation, and Social Protest.” Personality and Social Psychology Bulletin 13, no. 2: 275–283.

[jopy12980-bib-0091] Wang, Q. , N. A. Bowling , and K. J. Eschleman . 2010. “A Meta‐Analytic Examination of Work and General Locus of Control.” Journal of Applied Psychology 95, no. 4: 761–768. 20604595 10.1037/a0017707

[jopy12980-bib-0092] Wedell, D. H. , and A. Parducci . 2000. “Social Comparison: Lessons From Basic Research on Judgment.” In Handbook of Social Comparison: Theory and Research, edited by J. Suls and L. Wheeler , 223–252. New York, NY: Kluwer Academic Publishers.

[jopy12980-bib-0093] White, K. , and D. R. Lehman . 2005. “Culture and Social Comparison Seeking: The Role of Self‐Motives.” Personality and Social Psychology Bulletin 31, no. 2: 232–242. 15619595 10.1177/0146167204271326

[jopy12980-bib-0099] Wood, J. V. 1989. “Theory and Research Concerning Social Comparisons of Personal Attributes.” Psychological Bulletin 106: 231–248.

[jopy12980-bib-0094] Wrzus, C. , M. Hänel , J. Wagner , and F. J. Neyer . 2013. “Social Network Changes and Life Events Across the Life Span: A Meta‐Analysis.” Psychological Bulletin 139, no. 1: 53–80. 22642230 10.1037/a0028601

[jopy12980-bib-0095] Zainuddin, R. , and H. Taluja . 1990. “Aggression and Locus of Control Among Undergraduate Students.” Journal of Personality and Clinical Studies 6, no. 2: 211–215.

[jopy12980-bib-0096] Zell, E. , and M. D. Alicke . 2010. “The Local Dominance Effect in Self‐Evaluation: Evidence and Explanations.” Personality and Social Psychology Review 14, no. 4: 368–384. 20435806 10.1177/1088868310366144

