# Supplementary material for: Personal Relative Deprivation and Locus of Control
Source: J Pers. 2024 Oct 22;93(4):845–65. doi: 10.1111/jopy.12980 (PMC12224560; doi:10.1111/jopy.12980)
Supplement: Supplementary file 1 — Data S1. [file JOPY-93-845-s001.docx]

**SUPPLEMENTARY MATERIALS**

**List of Tables**

[**Table S1.** *Descriptive Statistics and Pearson’s Correlations among Measures (Study 1)* 1](#_Toc156559836)

[**Table S2.** *Kendall Correlations for Study 1* 2](#_Toc156559837)

[**Table S3.** *Unstandardised Regression Coefficients for Analyses across Studies 1 to 5* 3](#_Toc156559838)

[**Table S4.** *Additional Regression Models for Study 1 (Unstandardised Estimates)* 4](#_Toc156559839)

[**Table S5.** *Mediation Analyses (Study 1, Unstandardised Estimates)* 5](#_Toc156559840)

[**Table S6.** *Sample Characteristics (Study 2)* 6](#_Toc156559841)

[**Table S7.** *Correlations among Measures, Aggregated across Data Collection Regions (Study 2)* 7](#_Toc156559842)

[**Table S8.** *Fixed Effects Estimates for Study 2 Linear Mixed Effects Models* 10](#_Toc156559843)

[**Table S9.** *Descriptive Statistics and Correlations among Measures (Study 3)* 11](#_Toc156559844)

[**Table S10.** *Kendall Correlations for Study 3* 12](#_Toc156559845)

[**Table S11.** *Additional Regression Models for Study 3, Rotter LoC Scale (Unstandardised Estimates)* 13](#_Toc156559846)

[**Table S12.** *Descriptive Statistics and Correlations among Measures (Study 4)* 14](#_Toc156559847)

[**Table S13.** *Kendall Correlations for Study 4* 15](#_Toc156559848)

[**Table S14.** *Additional Regression Models for Study 4, Internal-to-External Explanations for Being Wealthy (Unstandardised Estimates)* 16](#_Toc156559849)

[**Table S15.** *Additional Regression Models for Study 4, Internal-to-External Explanations for Being Poor (Unstandardised Estimates)* 17](#_Toc156559850)

[**Table S16.** *Standardised Regression Coefficients for Analyses Predicting Perceived Constraints and Personal Mastery (Studies 1 and 7, Time 1)* 18](#_Toc156559851)

[**Table S17.** *Descriptive Statistics and Correlations among Measures (Study 5)* 19](#_Toc156559852)

[**Table S18.** *Kendall Correlations for Study 5* 20](#_Toc156559853)

[**Table S19.** *Additional Regression Models for Study 5, External Explanations (Unstandardised Estimates)* 21](#_Toc156559854)

[**Table S20.** *Additional Regression Models for Study 5, Internal Explanations (Unstandardised Estimates)* 22](#_Toc156559855)

[**Table S21.** *Additional Regression Models for Study 5, External – Internal Explanations (Unstandardised Estimates)* 23](#_Toc156559856)

[**Table S22.** *Descriptive Statistics and Correlations among Measures across Timepoints (Study 7)* 25](#_Toc156559857)

[**Table S23.** *Descriptive Statistics and Reliabilities for Daily Diary Measures across Days (Study 8)* 26](#_Toc156559858)

[**Table S24.** *Unstandardised Estimates for the Residual Covariances by Day for Study 8* 27](#_Toc156559859)

**List of Figures**

[**Figure S1.** *Correlations with 95% CIs between External Explanations and Predictors by Region of Data Collection (Study 2)* 8](#_Toc155094950)

[**Figure S2.** *Scatterplots with Fitted Lines for the Associations between the Predictors and External Explanations by Region and Larger Territory* 9](#_Toc155094951)

[**Figure S3.** *Graphical Representation of the RI-CLPM with Personal Relative Deprivation, Perceived Constraints, and Personal Mastery across the First Three Timepoints in Studies 7 and 8* 24](#_Toc155094952)

[**Figure S4.** *Effect of Comparison Direction as a Function of Type of Explanation (Preliminary Study)* 28](#_Toc155094953)

**List of Text**

[**Text S1: PRELIMINARY STUDY 6** 26](#_Toc148712157)

**Table S1.** *Descriptive Statistics and Pearson’s Correlations among Measures (Study 1)*

| Variable | *M* | *SD* | 1 | 2 | 3 | 4 | 5 | 6 | 7 | 8 |
| --- | --- | --- | --- | --- | --- | --- | --- | --- | --- | --- |
| 1. Explanations | 3.11 | 0.90 |  |  |  |  |  |  |  |  |
|  |  |  |  |  |  |  |  |  |  |  |
| 2. Control | 4.95 | 1.26 | -.53 (<.001) |  |  |  |  |  |  |  |
|  |  |  | [-.60, -.47] |  |  |  |  |  |  |  |
|  |  |  |  |  |  |  |  |  |  |  |
| 3. PRD | 3.24 | 1.16 | .31 (<.001) | -.65 (<.001) |  |  |  |  |  |  |
|  |  |  | [.23, .39] | [-.70, -.60] |  |  |  |  |  |  |
|  |  |  |  |  |  |  |  |  |  |  |
| 4. SSS | 4.57 | 1.73 | -.26 (<.001) | .41 (<.001) | -.54 (<.001) |  |  |  |  |  |
|  |  |  | [-.34, -.18] | [.33, .48] | [-.60, -.47] |  |  |  |  |  |
|  |  |  |  |  |  |  |  |  |  |  |
| 5. Income | 25.02 | 16.91 | -.18 (<.001) | .29 (<.001) | -.28 (<.001) | .49 (<.001) |  |  |  |  |
|  |  |  | [-.27, -.09] | [.21, .37] | [-.36, -.20] | [.41, .55] |  |  |  |  |
|  |  |  |  |  |  |  |  |  |  |  |
| 6. Education | 2.74 | 0.66 | -.05 (.297) | .12 (.010) | -.18 (<.001) | .39 (<.001) | .32 (<.001) |  |  |  |
|  |  |  | [-.14, .04] | [.03, .21] | [-.27, -.09] | [.31, .46] | [.23, .40] |  |  |  |
|  |  |  |  |  |  |  |  |  |  |  |
| 7. Political | 2.93 | 1.45 | -.37 (<.001) | .19 (<.001) | -.15 (.001) | .17 (<.001) | .05 (.271) | .05 (.271) |  |  |
|  |  |  | [-.45, -.29] | [.10, .27] | [-.24, -.06] | [.08, .25] | [-.04, .14] | [-.04, .14] |  |  |
|  |  |  |  |  |  |  |  |  |  |  |
| 8. Age | 35.76 | 11.75 | -.08 (.100) | .09 (.065) | -.12 (.010) | .09 (.060) | .08 (.092) | .08 (.102) | .13 (.004) |  |
|  |  |  | [-.17, .01] | [-.01, .18] | [-.21, -.03] | [-.00, .18] | [-.01, .17] | [-.02, .17] | [.04, .22] |  |
|  |  |  |  |  |  |  |  |  |  |  |
| 9. Gender | -- | -- | -.00 (.936) | -.08 (.102) | -.00 (.974) | .04 (.385) | -.06 (.185) | -.02 (.606) | -.00 (.916) | .19 (<.001) |
|  |  |  | [-.09, .09] | [-.17, .02] | [-.09, .09] | [-.05, .13] | [-.15, .03] | [-.11, .07] | [-.10, .09] | [.10, .28] |

*Note.* PRD = Personal Relative Deprivation. SSS = Subjective Socioeconomic Status. Values in parentheses and brackets indicate *p* values and 95% confidence intervals, respectively. *N* = 464*.*

**Table S2.** *Kendall Correlations for Study 1*

| Variable | Explanations | Control | PRD | SSS | Income | Education | Political | Age |
| --- | --- | --- | --- | --- | --- | --- | --- | --- |
| Control | -.39 (<.001) |  |  |  |  |  |  |  |
|  | [-.44, -.34] |  |  |  |  |  |  |  |
| PRD | .22 (<.001) | -.48 (<.001) |  |  |  |  |  |  |
|  | [.16, .28] | [-.53, -.43] |  |  |  |  |  |  |
| SSS | -.20 (<.001) | .29 (<.001) | -.41 (<.001) |  |  |  |  |  |
|  | [-.27, -.13] | [.23, .35] | [-.47, -.36] |  |  |  |  |  |
| Income | -.14 (<.001) | .22 (<.001) | -.21 (<.001) | .38 (<.001) |  |  |  |  |
|  | [-.20, -.08] | [.16, .28] | [-.27, -.15] | [.32, .43] |  |  |  |  |
| Education | -.04 (.298) | .08 (.023) | -.14 (<.001) | .33 (<.001) | .25 (<.001) |  |  |  |
|  | [-.11, .04] | [.01, .16] | [-.22, -.07] | [.26, .40] | [.18, .32] |  |  |  |
| Political | -.30 (<.001) | .13 (<.001) | -.10 (.003) | .13 (.001) | .04 (.260) | .05 (.181) |  |  |
|  | [-.36, -.23] | [.06, .20] | [-.17, -.03] | [.06, .20] | [-.03, .11] | [-.03, .13] |  |  |
| Age | -.03 (.315) | .04 (.163) | -.04 (.196) | .04 (.285) | .06 (.048) | .06 (.098) | .09 (.008) |  |
|  | [-.09, .03] | [-.01, .10] | [-.11, .02] | [-.03, .10] | [.00, .12] | [-.01, .13] | [.02, .16] |  |
| Gender | .01 (.764) | -.05 (.157) | -.00 (.954) | .04 (.343) | -.06 (.143) | -.03 (.567) | -.01 (.877) | .15 (<.001) |
|  | [-.06, .09] | [-.13, .02] | [-.08, .07] | [-.04, .12] | [-.13, .02] | [-.11, .06] | [-.09, .07] | [.07, .22] |

*Note.* PRD = Personal Relative Deprivation. SSS = Subjective Socioeconomic Status. Values in parentheses and brackets indicate *p* values and 95% confidence intervals, respectively.

**Table S3.** *Unstandardised Regression Coefficients for Analyses across Studies 1 to 5*

| Predictors | Study 1  (USA) | Study 2  (Asia) | Study 3  (USA) | Study 4  (UK) | | Study 5  (USA) | | |
| --- | --- | --- | --- | --- | --- | --- | --- | --- |
|  | *Internal-to-External Explanations* | *Internal-to-External Explanations* | *Rotter’s*  *LoC* | *Poverty External-Internal* | *Wealthy*  *External-Internal* | *External Explanations* | *Internal Explanations* | *External-Internal Explanations* |
| Intercept | **3.24** (<.001)  [2.69, 3.79] | **2.70** (<.001)  [2.20, 3.20] | **15.20** (<.001)  [12.33, 18.07] | **5.64** (<.001)  [4.30, 6.97] | **2.79** (<.001)  [1.44, 4.14] | **3.87** (<.001)  [3.24, 4.49] | **3.08** (<.001)  [2.51, 3.65] | 0.79 (.089)  [-0.12, 1.69] |
| PRD | **0.16** (<.001)  [0.09, 0.24] | **0.19** (<.001)  [0.14, 0.24] | **1.06** (<.001)  [0.64, 1.48] | -0.17 (.084)  [-0.36, 0.02] | **0.27** (.007)  [0.07, 0.46] | **0.19** (<.001)  [0.11, 0.28] | -0.06 (.154)  [-0.13, 0.02] | **0.25** (<.001)  [0.13, 0.37] |
| SSS | -0.04 (.17)  [-0.10, 0.02] | 0.02 (.137)  [-0.00, 0.04] | -0.10 (.491)  [-0.37, 0.18] | **-0.14** (.033)  [-0.27, -0.01] | -0.12 (.076)  [-0.25, 0.01] | 0.04 (.182)  [-0.02, 0.09] | **0.14** (<.001)  [0.09, 0.19] | **-0.11** (.006)  [-0.18, -0.03] |
| Income | -0.00 (.075)  [-0.01, 0.00] | -- | 0.00 (.899)  [-0.02, 0.02] | 0.00 (.521)  [-0.01, 0.02] | - 1. (.389)   [-0.01, 0.02] | **-0.01** (<.001)  [-0.01, -0.00] | -0.00 (.195)  [-0.01, 0.00] | **-0.01** (.048)  [-0.01, -0.00] |
| Education | 0.09 (.164)  [-0.04, 0.21] | -- | -0.17 (.529)  [-0.71, 0.37] | 0.16 (.108)  [-0.04, 0.36] | **0.23** (.023)  [0.03, 0.43] | 0.13 (.061)  [-0.01, 0.28] | -0.00 (.963)  [-0.13, 0.13] | 0.14 (.186)  [-0.07, 0.34] |
| Political | **-0.20** (<.001)  [-0.25, -0.15] | -0.01 (.808)  [-0.07, 0.06] | **-0.50** (<.001)  [-0.74, -0.26] | **-0.90** (<.001)  [-1.04, -0.76] | **-0.86** (<.001)  [-1.01, -0.72] | **-0.18** (<.001)  [-0.24, -0.13] | **0.17** (<.001)  [0.12, 0.21] | **-0.35** (<.001)  [-0.42, -0.28] |
| Age | 0.00 (.953)  [-0.01, 0.01] | 0.00 (0.94)  [-0.01, 0.01] | **-0.08** (<.001)  [-0.11, -0.05] | 0.00 (.757)  [-0.01, 0.01] | 0.00 (.795)  [-0.01, 0.01] | -0.00 (.854)  [-0.01, 0.01] | **0.01** (.043)  [0.00, 0.01] | -0.01 (.159)  [-0.02, 0.00] |
| Gender | -0.01 (0.88)  [-0.16, 0.14] | 0.08 (.154)  [-0.03, 0.18] | **1.29** (.001)  [0.50, 2.07] | -0.02 (.920)  [-0.38, 0.35] | -0.06 (.730)  [-0.43, 0.30] | 0.15 (.070)  [-0.01, 0.32] | **-0.20** (.009)  [-0.35, -0.05] | **0.35** (.004)  [0.11, 0.59] |
| *N* | 464 | 3,851 | 429 | 448 | 448 | 540 | 540 | 540 |
| *R^2^/R^2^_adj_* | .220/.208 | .193^a^ | .229/.216 | .322/.311 | .334/.324 | .183 / .172 | .203/.193 | .261/.251 |

*Note.* Bolded values show those estimates where the corresponding 95% CI does not include zero.

**Table S4.** *Additional Regression Models for Study 1 (Unstandardised Estimates)*

| Predictors | **Log Income** | **Education**  **as Factor** | **Robust** | **Robust**  **Log Income** | **Robust Education**  **as Factor** |
| --- | --- | --- | --- | --- | --- |
| Intercept | 3.47 ^***^ [2.88 – 4.05] | 3.49 ^***^ [2.98 – 4.00] | 3.27 ^***^ [2.77 – 3.76] | 3.52 ^***^ [2.99 – 4.04] | 3.61 ^***^ [3.15 – 4.07] |
| PRD | 0.16 ^***^ [0.09 – 0.24] | 0.16 ^***^ [0.08 – 0.23] | 0.15 ^***^ [0.08 – 0.22] | 0.15 ^***^ [0.08 – 0.22] | 0.15 ^***^ [0.08 – 0.21] |
| SSS | -0.04  [-0.09 – 0.02] | -0.04  [-0.10 – 0.02] | -0.04  [-0.10 – 0.01] | -0.04  [-0.09 – 0.01] | -0.04  [-0.10 – 0.01] |
| Log Income | -0.29 ^*^ [-0.56 – -0.02] |  |  | -0.30 ^*^ [-0.54 – -0.05] |  |
| Education | 0.08  [-0.04 – 0.20] |  | 0.11 ^*^ [0.00 – 0.22] | 0.11 ^*^ [0.00 – 0.22] |  |
| Political | -0.20 ^***^ [-0.25 – -0.15] | -0.20 ^***^ [-0.25 – -0.15] | -0.22 ^***^ [-0.27 – -0.18] | -0.22 ^***^ [-0.27 – -0.17] | -0.22 ^***^ [-0.27 – -0.17] |
| Age | 0.00  [-0.01 – 0.01] | -0.00  [-0.01 – 0.01] | -0.00  [-0.01 – 0.01] | 0.00  [-0.01 – 0.01] | -0.00  [-0.01 – 0.01] |
| Gender | -0.02  [-0.17 – 0.13] | -0.00  [-0.16 – 0.15] | 0.04  [-0.09 – 0.18] | 0.04  [-0.10 – 0.18] | 0.06  [-0.08 – 0.19] |
| Income |  | -0.00  [-0.01 – 0.00] | -0.00 ^*^ [-0.01 – -0.00] |  | -0.01 ^*^ [-0.01 – -0.00] |
| Educ [high school] |  | -0.06  [-0.16 – 0.05] |  |  | -0.07  [-0.16 – 0.03] |
| Educ [college] |  | -0.02  [-0.09 – 0.05] |  |  | -0.01  [-0.08 – 0.05] |
| Educ [postgrad] |  | 0.22 ^*^ [0.00 – 0.43] |  |  | 0.25 ^*^ [0.06 – 0.44] |
| *N* | 464 | 464 | 464 | 464 | 464 |
| *R^2^/R^2^_adj_* | 0.222 / 0.210 | 0.227 / 0.212 | 0.272 / 0.260 | 0.274 / 0.263 | 0.277 / 0.262 |
| * p<0.05   ** p<0.01   *** p<0.001 | | | | | |

**Table S5.** *Mediation Analyses (Study 1, Unstandardised Estimates)*

|  | **Sense of Control (a-path)** | | | | **Internal vs. External**  **Explanations (b-path)** | | | |
| --- | --- | --- | --- | --- | --- | --- | --- | --- |
| **Predictors** | *B (se)* | *95% CI* | *β* | *p* | *B (se)* | *95% CI* | *β* | *p* |
| (Intercept) | 6.66 (0.33) | 6.02 – 7.30 | 0.00 | <.001 | 5.75 (0.35) | 5.07 – 6.43 | 0.00 | <.001 |
| PRD | **-0.64** (0.04) | -0.73 – -0.55 | -0.59 | <.001 | -0.08 (0.04) | -0.16 – 0.00 | -0.10 | .054 |
| SSS | 0.04 (0.03) | -0.03 – 0.10 | 0.05 | .304 | -0.03 (0.03) | -0.08 – 0.02 | -0.05 | .307 |
| Income | **0.01** (0.00) | 0.00 – 0.01 | 0.11 | .009 | -0.00 (0.00) | -0.01 – 0.00 | -0.03 | .491 |
| Education | -0.09 (0.07) | -0.23 – 0.05 | -0.05 | .204 | 0.05 (0.06) | -0.06 – 0.16 | 0.04 | .354 |
| Political | **0.07** (0.03) | 0.01 – 0.13 | 0.09 | .016 | **-0.18** (0.02) | -0.22 – -0.13 | -0.28 | <.001 |
| Age | 0.00 (0.00) | -0.01 – 0.01 | 0.01 | .811 | 0.00 (0.00) | -0.01 – 0.01 | 0.01 | .855 |
| Gender | **-0.19** (0.09) | -0.36 – -0.01 | -0.15 | .036 | -0.08 (0.07) | -0.22 – 0.05 | -0.05 | .236 |
| Sense of  Control | -- | -- | -- | -- | **-0.38** (0.04) | -0.45 – -0.31 | -0.53 | <0.001 |
| *N* | 464 | | | | 464 | | | |
| *R^2^/R^2^_adj_* | 0.456 / 0.447 | | | | 0.371 / 0.360 | | | |

*Note.* PRD = Personal Relative Deprivation. SSS = Subjective Socioeconomic Status. Bolded values show those estimates where the corresponding 95% CI does not include zero.

**Table S6.** *Sample Characteristics (Study 2)*

| Site | *N* | Age | % Female | Explanations | PRD | SSS | Political | Language |
| --- | --- | --- | --- | --- | --- | --- | --- | --- |
| China (Mainland) |  |  |  |  |  |  |  |  |
| Guangzhou | 152 | 20 (1.43) | 80 | 2.90 (0.75) | 3.15 (1.05) | 5.13 (1.33) | 3.05 (0.92) | Simplified Chinese |
| Shanghai | 111 | 26 (9.05) | 70 | 2.99 (0.87) | 2.80 (1.01) | 4.97 (1.5) | 2.97 (0.99) | Simplified Chinese |
| Wuhan | 194 | 20.73 (2.93) | 30 | 3.16 (0.97) | 3.81 (1.38) | 5.29 (1.64) | 3.28 (1.22) | Simplified Chinese |
| Hong Kong (S.A.R.) |  |  |  |  |  |  |  |  |
| Hong Kong | 223 | 19.36 (2.84) | 64 | 3.63 (0.88) | 3.54 (0.89) | 5.11 (1.59) | 3.10 (1.01) | English |
| India |  |  |  |  |  |  |  |  |
| Kolkata | 123 | 24.76 (6.56) | 61 | 3.25 (0.84) | 2.82 (1.05) | 5.72 (1.33) | 2.74 (1.27) | English |
| New Delhi | 162 | 21.66 (3.31) | 61 | 3.38 (0.90) | 3.03 (1.09) | 6.23 (1.63) | 2.69 (1.19) | English |
| Puducherry | 199 | 23.72 (5.90) | 36 | 3.77 (1.09) | 3.41 (0.94) | 5.82 (1.85) | 3.84 (0.98) | English |
| Japan |  |  |  |  |  |  |  |  |
| Kochi | 196 | 19.12 (1.27) | 44 | 2.79 (0.87) | 2.43 (0.93) | 5.24 (1.71) | 3.63 (1.21) | Japanese |
| Kurume | 85 | 19.11 (1.22) | 60 | 3.25 (0.90) | 2.73 (1.19) | 5.27 (1.79) | 3.74 (1.16) | Japanese |
| Nagoya | 494 | 19.72 (1.39) | 38 | 3.26 (0.99) | 2.69 (1.09) | 5.79 (1.89) | 3.75 (1.24) | Japanese |
| Osaka | 269 | 20.01 (0.97) | 66 | 3.03 (0.90) | 2.46 (1.07) | 6.01 (1.65) | 3.61 (1.09) | Japanese |
| Tokyo | 141 | 21 (1.56) | 75 | 3.42 (0.95) | 2.65 (1.08) | 5.80 (1.67) | 3.55 (1.11) | Japanese |
| Macau (S.A.R.) |  |  |  |  |  |  |  |  |
| Macau | 310 | 19.67 (1.40) | 58 | 3.41 (0.85) | 3.31 (0.93) | 5.31 (1.46) | 2.67 (1.01) | Traditional Chinese |
| South Korea |  |  |  |  |  |  |  |  |
| Pohang | 134 | 24.03 (2.47) | 62 | 3.85 (0.81) | 2.57 (1.11) | 5.76 (1.47) | 3.33 (0.91) | Korean |
| Seoul | 354 | 22.54 (2.80) | 51 | 3.80 (0.89) | 2.85 (1.04) | 6.22 (1.65) | 3.25 (0.90) | Korean |
| Taiwan |  |  |  |  |  |  |  |  |
| Chiayi | 224 | 20.12 (1.68) | 78 | 3.41 (0.82) | 3.32 (1.04) | 5.66 (1.33) | 2.62 (0.96) | Traditional Chinese |
| Kaohsiung | 251 | 19.49 (1.94) | 59 | 3.43 (0.67) | 3.21 (0.98) | 6.03 (1.38) | 2.57 (0.92) | Traditional Chinese |
| Taipei | 229 | 24.39 (5.65) | 54 | 3.45 (0.72) | 3.40 (0.99) | 5.90 (1.46) | 2.76 (0.97) | Traditional Chinese |
| Total | 3,851 | 21.11 (3.83) | 56 | 3.36 (0.93) | 3.02 (1.11) | 5.68 (1.65) | 3.19 (1.15) |  |

*Note.* Values show means (*SD*s) by region except for *N* and % Female

**Table S7.** *Correlations among Measures, Aggregated across Data Collection Regions (Study 2)*

| Variable | 1 | 2 | 3 | 4 | 5 |
| --- | --- | --- | --- | --- | --- |
| 1. Explanations |  |  |  |  |  |
|  |  |  |  |  |  |
| 2. PRD | .24 (<.001) |  |  |  |  |
|  | [.21, .27] |  |  |  |  |
|  |  |  |  |  |  |
| 3. SSS | .05 (<.001) | -.11 (<.001) |  |  |  |
|  | [.02, .08] | [-.15, -.08] |  |  |  |
|  |  |  |  |  |  |
| 4. Political | -.02 (.179) | -.03 (.080) | -.01 (.601) |  |  |
|  | [-.05, .01] | [-.06, .00] | [-.04, .02] |  |  |
|  |  |  |  |  |  |
| 5. Age | .04 (.025) | -.01 (.702) | .03 (.059) | -.02 (.183) |  |
|  | [.00, .07] | [-.04, .03] | [-.00, .06] | [-.05, .01] |  |
|  |  |  |  |  |  |
| 6. Gender | .03 (.117) | -.07 (<.001) | .00 (.927) | -.03 (.041) | -.06 (<.001) |
|  | [-.01, .06] | [-.10, -.03] | [-.03, .03] | [-.06, -.00] | [-.09, -.03] |

*Note.* PRD = Personal Relative Deprivation. SSS = Subjective Socioeconomic Status. Values in parentheses and brackets indicate *p* values and 95% confidence intervals, respectively. *N* = 3,851.

**Figure S1.** *Correlations with 95% CIs between External Explanations and Predictors by Region of Data Collection (Study 2)*

**
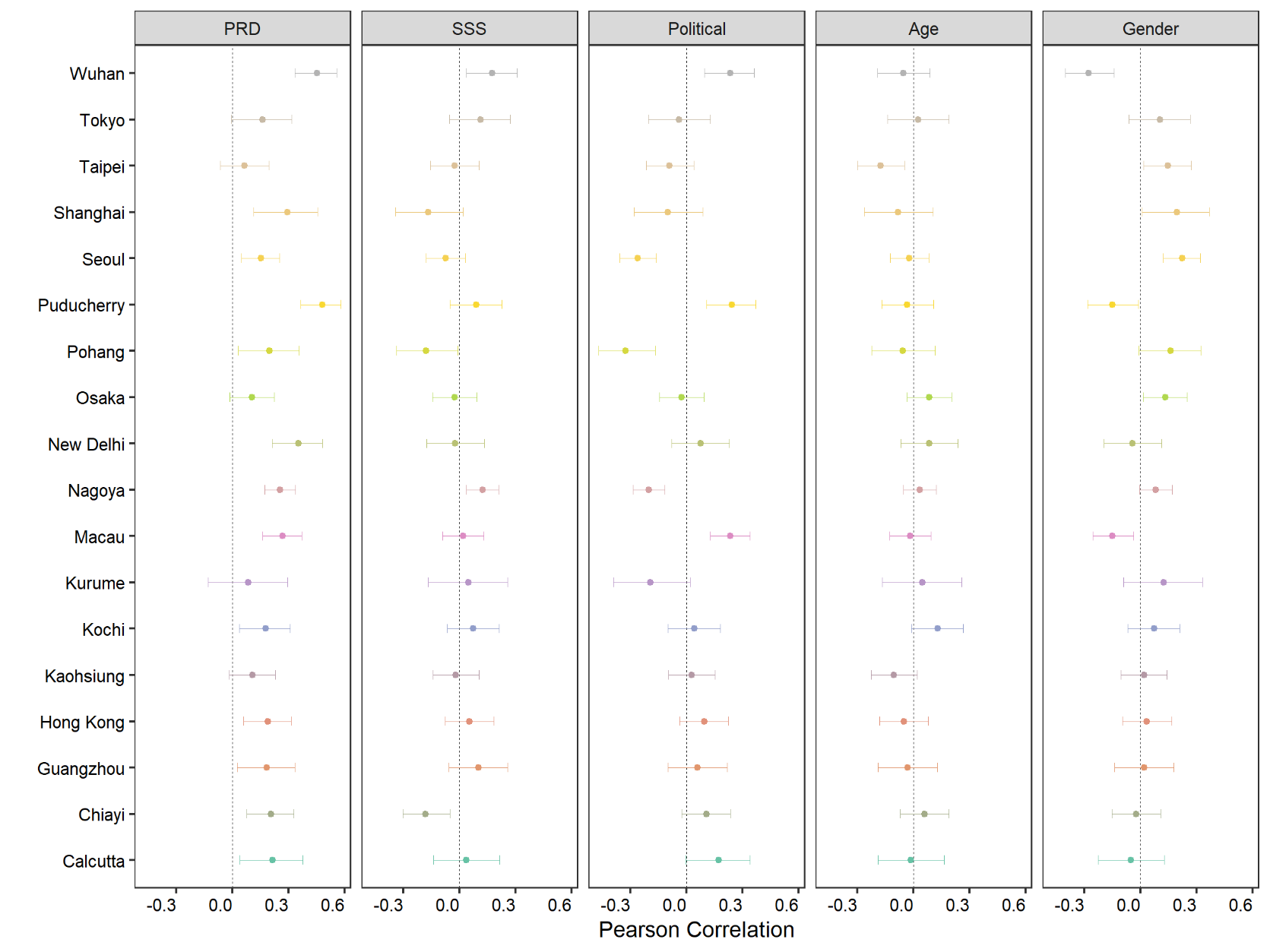
**

**Figure S2.** *Scatterplots with Fitted Lines for the Associations between the Predictors and External Explanations by Region and Territory*

*
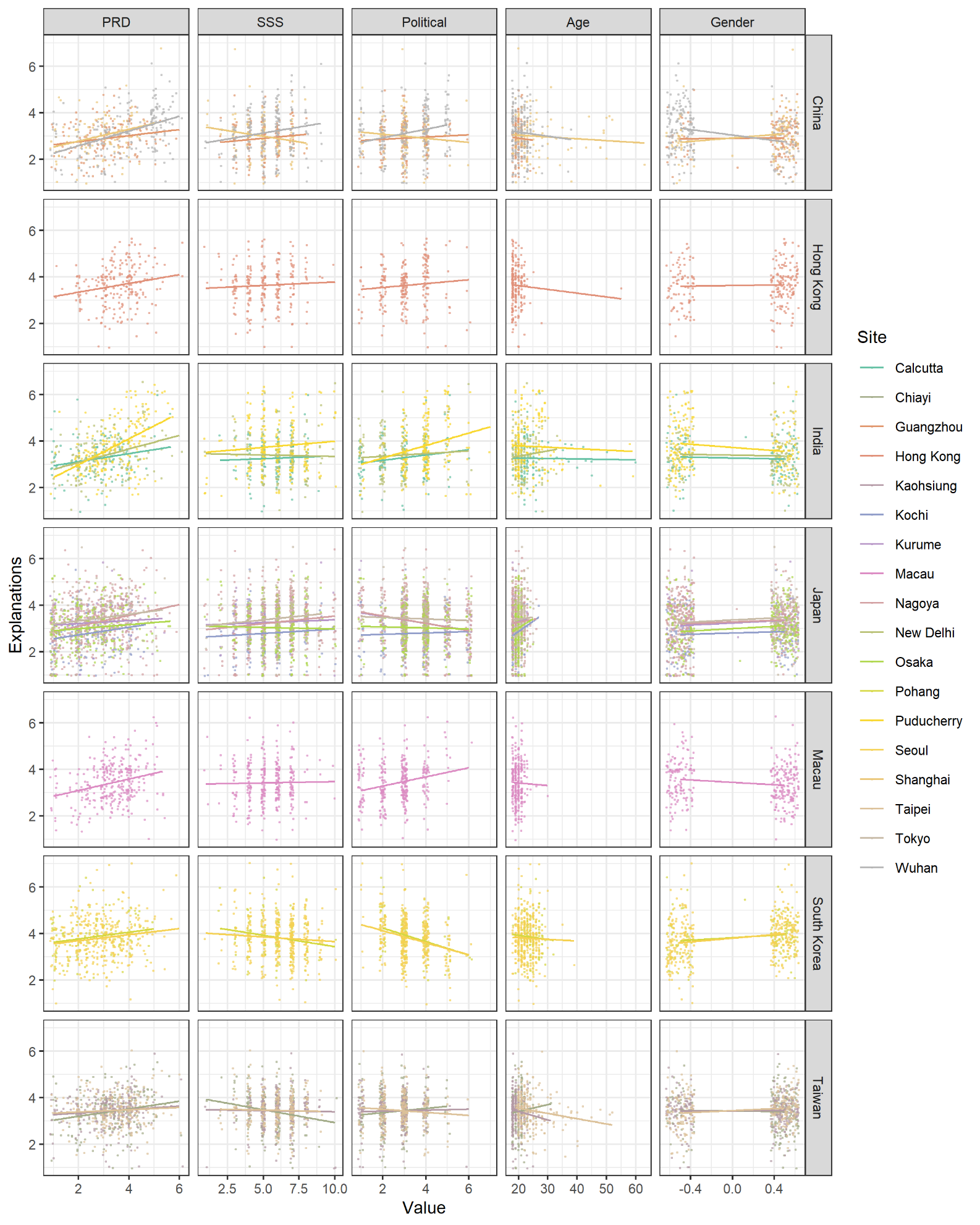
*

**Table S8.** *Fixed Effects Estimates for Study 2 Linear Mixed Effects Models*

|  | **Standardised estimates** | | | |  | **Unstandardised estimates** | | | |
| --- | --- | --- | --- | --- | --- | --- | --- | --- | --- |
| Predictors | *Β (SE)* | *95% CI* | *p* | *df* |  | *B (SE)* | *95% CI* | *p* | *df* |
| **Maximal model, nesting within sites** | | | |  |  |  |  |  |  |
| PRD | 0.23 (0.03) | [0.16, 0.29] | <0.001 | 16.44 |  | 0.19 (0.03) | [0.13, 0.24] | <0.001 | 16.44 |
| SSS | 0.03 (0.02) | [-0.01, 0.07] | 0.136 | 25.71 |  | 0.01 (0.01) | [-0.01, 0.04] | 0.136 | 25.71 |
| Political | -0.01 (0.04) | [-0.09, 0.07] | 0.807 | 16.92 |  | -0.01 (0.03) | [-0.08, 0.06] | 0.807 | 16.92 |
| Age | 0.00 (0.03) | [-0.06, 0.07] | 0.946 | 9.81 |  | 0.00 (0.01) | [-0.01, 0.02] | 0.946 | 9.81 |
| Gender | 0.09 (0.06) | [-0.03, 0.21] | 0.153 | 18.32 |  | 0.08 (0.05) | [-0.03, 0.19] | 0.153 | 18.32 |
| **Uncorrelated random effects, nesting within sites** | | | | |  |  |  |  |  |
| PRD | 0.23 (0.03) | [0.16, 0.29] | <0.001 | 15.91 |  | 0.19 (0.02) | [0.13, 0.24] | <0.001 | 15.72 |
| SSS | 0.03 (0.02) | [-0.01, 0.07] | 0.106 | 16.38 |  | 0.02 (0.01) | [-0.00, 0.04] | 0.054 | 22.54 |
| Political | -0.01 (0.04) | [-0.09, 0.07] | 0.811 | 16.88 |  | -0.01 (0.03) | [-0.07, 0.05] | 0.820 | 17.00 |
| Age | 0.00 (0.03) | [-0.06, 0.06] | 0.999 | 7.20 |  | 0.00 (0.01) | [-0.01, 0.01] | 0.580 | 12.28 |
| Gender | 0.09 (0.06) | [-0.03, 0.20] | 0.146 | 17.19 |  | 0.08 (0.05) | [-0.03, 0.19] | 0.152 | 17.09 |
| **Maximal model, sites nested within larger territories** | | | | | |  |  |  |  |
| PRD | 0.23 (0.04) | [0.14, 0.32] | 0.002 | 5.71 |  | 0.19 (0.03) | [0.12, 0.26] | 0.002 | 5.81 |
| SSS | 0.02 (0.02) | [-0.03, 0.07] | 0.424 | 7.05 |  | 0.01 (0.01) | [-0.02, 0.04] | 0.400 | 6.94 |
| Political | 0.01 (0.06) | [-0.12, 0.13] | 0.932 | 5.14 |  | 0.00 (0.05) | [-0.09, 0.10] | 0.930 | 5.14 |
| Age | 0.00 (0.05) | [-0.10, 0.11] | 0.958 | 4.45 |  | 0.00 (0.01) | [-0.03, 0.03] | 0.948 | 4.62 |
| Gender | 0.06 (0.08) | [-0.12, 0.23] | 0.529 | 5.84 |  | 0.05 (0.08) | [-0.11, 0.21] | 0.522 | 5.87 |
|  |  |  |  |  |  |  |  |  |  |

*Note.* For the standardised estimates, all variables were z-scored except gender.

**Table S9.** *Descriptive Statistics and Correlations among Measures (Study 3)*

| Variable | *M* | *SD* | 1 | 2 | 3 | 4 | 5 | 6 | 7 |
| --- | --- | --- | --- | --- | --- | --- | --- | --- | --- |
| 1. LoC | 13.08 | 4.50 |  |  |  |  |  |  |  |
|  |  |  |  |  |  |  |  |  |  |
| 2. PRD | 3.09 | 1.07 | .32 (<.001) |  |  |  |  |  |  |
|  |  |  | [.24, .41] |  |  |  |  |  |  |
|  |  |  |  |  |  |  |  |  |  |
| 3. SSS | 5.25 | 1.82 | -.21 (<.001) | -.54 (<.001) |  |  |  |  |  |
|  |  |  | [-.30, -.11] | [-.60, -.47] |  |  |  |  |  |
|  |  |  |  |  |  |  |  |  |  |
| 4. Income | 31.67 | 26.16 | -.12 (.013) | -.28 (<.001) | .44 (<.001) |  |  |  |  |
|  |  |  | [-.21, -.03] | [-.36, -.19] | [.36, .52] |  |  |  |  |
|  |  |  |  |  |  |  |  |  |  |
| 5. Education | 2.83 | 0.77 | -.12 (.010) | -.20 (<.001) | .35 (<.001) | .27 (<.001) |  |  |  |
|  |  |  | [-.22, -.03] | [-.29, -.10] | [.26, .43] | [.18, .36] |  |  |  |
|  |  |  |  |  |  |  |  |  |  |
| 6. Political | 3.38 | 1.64 | -.25 (<.001) | -.07 (.148) | .08 (.102) | .03 (.496) | -.05 (.286) |  |  |
|  |  |  | [-.34, -.16] | [-.16, .02] | [-.02, .17] | [-.06, .13] | [-.15, .04] |  |  |
|  |  |  |  |  |  |  |  |  |  |
| 7. Age | 35.54 | 13.53 | -.30 (<.001) | -.15 (.002) | .04 (.407) | .11 (.020) | .16 (<.001) | .16 (.001) |  |
|  |  |  | [-.38, -.21] | [-.24, -.05] | [-.05, .13] | [.02, .20] | [.07, .25] | [.06, .25] |  |
|  |  |  |  |  |  |  |  |  |  |
| 8. Gender | -- | -- | .14 (.005) | -.01 (.805) | -.01 (.915) | .02 (.736) | -.03 (.603) | -.10 (.033) | .09 (.065) |
|  |  |  | [.04, .23] | [-.11, .08] | [-.10, .09] | [-.08, .11] | [-.12, .07] | [-.20, -.01] | [-.01, .18] |

*Note.* LoC = Locus of Control. PRD = Personal Relative Deprivation. SSS = Subjective Socioeconomic Status. Values in parentheses and brackets indicate *p* values and 95% confidence intervals, respectively. *N* = 429.

**Table S10.** *Kendall Correlations for Study 3*

| Variable | LoC | PRD | SSS | Income | Education | Political | Age |
| --- | --- | --- | --- | --- | --- | --- | --- |
| PRD | .22 (<.001) |  |  |  |  |  |  |
|  | [.15, .28] |  |  |  |  |  |  |
| SSS | -.13 (<.001) | -.40 (<.001) |  |  |  |  |  |
|  | [-.20, -.07] | [-.46, -.34] |  |  |  |  |  |
| Income | -.09 (.006) | -.19 (<.001) | .37 (<.001) |  |  |  |  |
|  | [-.16, -.03] | [-.26, -.13] | [.31, .43] |  |  |  |  |
| Education | -.09 (.018) | -.16 (<.001) | .29 (<.001) | .26 (<.001) |  |  |  |
|  | [-.17, -.01] | [-.23, -.08] | [.22, .37] | [.20, .33] |  |  |  |
| Political | -.20 (<.001) | -.05 (.136) | .06 (.109) | .03 (.432) | -.05 (.214) |  |  |
|  | [-.28, -.13] | [-.13, .02] | [-.02, .14] | [-.04, .10] | [-.13, .03] |  |  |
| Age | -.19 (<.001) | -.10 (.003) | .03 (.459) | .09 (.005) | .18 (<.001) | .11 (.002) |  |
|  | [-.25, -.12] | [-.16, -.04] | [-.04, .09] | [.03, .16] | [.10, .25] | [.04, .18] |  |
| Gender | .10 (.010) | -.01 (.748) | .00 (.975) | .01 (.800) | -.02 (.711) | -.10 (.016) | .06 (.120) |
|  | [.02, .18] | [-.09, .06] | [-.08, .08] | [-.07, .09] | [-.10, .07] | [-.18, -.02] | [-.02, .14] |

*Note.* PRD = Personal Relative Deprivation. SSS = Subjective Socioeconomic Status. Values in parentheses and brackets indicate *p* values and 95% confidence intervals, respectively.

**Table S11.** *Additional Regression Models for Study 3, Rotter LoC Scale (Unstandardised Estimates)*

| Predictors | **Log Income** | **Education**  **as Factor** | **Robust** | **Robust**  **Log Income** | **Robust Education**  **as Factor** |
| --- | --- | --- | --- | --- | --- |
| Intercept | 15.21 ^***^ [12.29, 18.13] | 14.58 ^***^ [11.85, 17.31] | 15.41 ^***^ [12.44, 18.39] | 15.49 ^***^ [12.46, 18.51] | 14.72 ^***^ [11.87, 17.57] |
| PRD | 1.06 ^***^ [0.64, 1.48] | 1.09 ^***^ [0.67, 1.51] | 1.10 ^***^ [0.66, 1.54] | 1.10 ^***^ [0.66, 1.54] | 1.10 ^***^ [0.66, 1.54] |
| SSS | -0.09  [-0.37, 0.20] | -0.09  [-0.37, 0.18] | -0.08  [-0.36, 0.20] | -0.07  [-0.36, 0.23] | -0.08  [-0.37, 0.20] |
| Log Income | -0.05  [-1.17, 1.08] |  |  | -0.15  [-1.31, 1.02] |  |
| Education | -0.16  [-0.71, 0.38] |  | -0.22  [-0.78, 0.34] | -0.21  [-0.77, 0.36] |  |
| Political | -0.50 ^***^ [-0.74, -0.26] | -0.48 ^***^ [-0.72, -0.24] | -0.55 ^***^ [-0.80, -0.31] | -0.55 ^***^ [-0.80, -0.31] | -0.53 ^***^ [-0.77, -0.28] |
| Age | -0.08 ^***^ [-0.11, -0.05] | -0.08 ^***^ [-0.11, -0.05] | -0.08 ^***^ [-0.11, -0.05] | -0.08 ^***^ [-0.11, -0.05] | -0.08 ^***^ [-0.11, -0.05] |
| Gender | 1.29 ^**^ [0.50, 2.07] | 1.25 ^**^ [0.46, 2.03] | 1.18 ^**^ [0.36, 1.99] | 1.18 ^**^ [0.36, 1.99] | 1.17 ^**^ [0.35, 1.98] |
| Income |  | 0.00  [-0.02, 0.02] | 0.00  [-0.02, 0.02] |  | 0.00  [-0.02, 0.02] |
| Educ [high school] |  | -0.06  [-0.61, 0.50] |  |  | 0.02  [-0.55, 0.60] |
| Educ [college] |  | -0.12  [-0.56, 0.32] |  |  | -0.12  [-0.57, 0.33] |
| Educ [postgrad] |  | 0.04  [-0.74, 0.81] |  |  | -0.08  [-0.89, 0.72] |
| *N* | 429 | 429 | 429 | 429 | 429 |
| *R^2^/R^2^_adj_* | 0.229 / 0.216 | 0.240 / 0.224 | 0.237 / 0.225 | 0.237 / 0.225 | 0.242 / 0.225 |
| * p<0.05   ** p<0.01   *** p<0.001 | | | | | |

*Note.* PRD = Personal Relative Deprivation. SSS = Subjective Socioeconomic Status. Values in brackets indicate 95% confidence intervals.

**Table S12.** *Descriptive Statistics and Correlations among Measures (Study 4)*

| Variable | *M* | *SD* | 1 | 2 | 3 | 4 | 5 | 6 | 7 | 8 |
| --- | --- | --- | --- | --- | --- | --- | --- | --- | --- | --- |
| 1. Wealthy | 0.71 | 2.20 |  |  |  |  |  |  |  |  |
|  |  |  |  |  |  |  |  |  |  |  |
| 2. Poverty | 1.63 | 2.16 | .72 (<.001) |  |  |  |  |  |  |  |
|  |  |  | [.67, .76] |  |  |  |  |  |  |  |
|  |  |  |  |  |  |  |  |  |  |  |
| 3. PRD | 2.89 | 0.98 | .22 (<.001) | .04 (.389) |  |  |  |  |  |  |
|  |  |  | [.13, .30] | [-.05, .13] |  |  |  |  |  |  |
|  |  |  |  |  |  |  |  |  |  |  |
| 4. SSS | 5.34 | 1.60 | -.19 (<.001) | -.14 (.003) | -.44 (<.001) |  |  |  |  |  |
|  |  |  | [-.28, -.10] | [-.23, -.05] | [-.51, -.36] |  |  |  |  |  |
|  |  |  |  |  |  |  |  |  |  |  |
| 5. Income | 18.44 | 14.70 | -.01 (.756) | -.01 (.798) | -.16 (<.001) | .39 (<.001) |  |  |  |  |
|  |  |  | [-.11, .08] | [-.10, .08] | [-.25, -.07] | [.31, .46] |  |  |  |  |
|  |  |  |  |  |  |  |  |  |  |  |
| 6. Education | 3.65 | 0.96 | .16 (<.001) | .15 (.002) | -.08 (.099) | .32 (<.001) | .35 (<.001) |  |  |  |
|  |  |  | [.07, .25] | [.06, .24] | [-.17, .01] | [.24, .40] | [.26, .42] |  |  |  |
|  |  |  |  |  |  |  |  |  |  |  |
| 7. Political | 3.71 | 1.28 | -.55 (<.001) | -.56 (<.001) | -.15 (.002) | .19 (<.001) | .07 (.151) | -.17 (<.001) |  |  |
|  |  |  | [-.61, -.48] | [-.62, -.49] | [-.24, -.06] | [.10, .28] | [-.02, .16] | [-.26, -.08] |  |  |
|  |  |  |  |  |  |  |  |  |  |  |
| 8. Age | 36.57 | 14.05 | -.10 (.030) | -.10 (.036) | -.16 (<.001) | .04 (.397) | .03 (.482) | -.03 (.500) | .17 (<.001) |  |
|  |  |  | [-.19, -.01] | [-.19, -.01] | [-.25, -.07] | [-.05, .13] | [-.06, .13] | [-.12, .06] | [.08, .26] |  |
|  |  |  |  |  |  |  |  |  |  |  |
| 9. Gender | -- | -- | .01 (.781) | .04 (.345) | -.11 (.021) | .03 (.532) | .01 (.776) | .02 (.649) | -.08 (.099) | .04 (.453) |
|  |  |  | [-.08, .11] | [-.05, .14] | [-.20, -.02] | [-.06, .12] | [-.08, .11] | [-.07, .11] | [-.17, .01] | [-.06, .13] |

*Note.* Wealthy = internal-to-external explanations for being wealthy. Poverty = internal-to-external explanations for being poor. PRD = Personal Relative Deprivation. SSS = Subjective Socioeconomic Status. Values in parentheses and brackets indicate *p* values and 95% confidence intervals, respectively. *N* = 448*.*

**Table S13.** *Kendall Correlations for Study 4*

| Variable | Wealthy | Poverty | PRD | SSS | Income | Education | Political | Age |
| --- | --- | --- | --- | --- | --- | --- | --- | --- |
| Poverty | .54 (<.001) |  |  |  |  |  |  |  |
|  | [.49, .58] |  |  |  |  |  |  |  |
| PRD | .14 (<.001) | .02 (.635) |  |  |  |  |  |  |
|  | [.08, .20] | [-.05, .08] |  |  |  |  |  |  |
| SSS | -.12 (<.001) | -.09 (.007) | -.33 (<.001) |  |  |  |  |  |
|  | [-.19, -.06] | [-.16, -.02] | [-.39, -.27] |  |  |  |  |  |
| Income | -.02 (.572) | .00 (.982) | -.11 (.001) | .31 (<.001) |  |  |  |  |
|  | [-.08, .05] | [-.06, .06] | [-.18, -.05] | [.25, .37] |  |  |  |  |
| Education | .12 (.001) | .12 (.001) | -.05 (.153) | .26 (<.001) | .27 (<.001) |  |  |  |
|  | [.05, .19] | [.05, .19] | [-.12, .02] | [.19, .33] | [.20, .33] |  |  |  |
| Political | -.43 (<.001) | -.44 (<.001) | -.11 (.003) | .14 (<.001) | .05 (.135) | -.17 (<.001) |  |  |
|  | [-.49, -.37] | [-.49, -.38] | [-.18, -.03] | [.06, .21] | [-.02, .12] | [-.24, -.09] |  |  |
| Age | -.07 (.027) | -.07 (.039) | -.10 (.001) | .02 (.497) | .07 (.041) | .02 (.491) | .13 (<.001) |  |
|  | [-.14, -.01] | [-.13, -.00] | [-.17, -.04] | [-.04, .09] | [.00, .13] | [-.04, .09] | [.05, .20] |  |
| Gender | .01 (.763) | .03 (.436) | -.09 (.018) | .03 (.467) | .01 (.833) | .03 (.463) | -.08 (.065) | .02 (.597) |
|  | [-.06, .09] | [-.05, .11] | [-.17, -.02] | [-.05, .11] | [-.07, .08] | [-.05, .12] | [-.16, .00] | [-.05, .10] |

*Note.* Wealthy = internal-to-external explanations for being wealthy. Poverty = internal-to-external explanations for being poor. PRD = Personal Relative Deprivation. SSS = Subjective Socioeconomic Status. Values in parentheses and brackets indicate *p* values and 95% confidence intervals, respectively.

**Table S14.** *Additional Regression Models for Study 4, Internal-to-External Explanations for Being Wealthy (Unstandardised Estimates)*

| Predictors | **Log Income** | **Education**  **as Factor** | **Robust** | **Robust**  **Log Income** | **Robust Education**  **as Factor** |
| --- | --- | --- | --- | --- | --- |
| (Intercept) | 2.63 ^***^ [1.28, 3.98] | 3.63 ^***^ [2.39, 4.88] | 2.83 ^***^ [1.43, 4.23] | 2.71 ^***^ [1.31, 4.10] | 3.74 ^***^ [2.45, 5.03] |
| PRD | 0.26 ^**^ [0.07, 0.46] | 0.28 ^**^ [0.08, 0.48] | 0.27 ^**^ [0.07, 0.47] | 0.27 ^**^ [0.07, 0.47] | 0.28 ^**^ [0.08, 0.49] |
| SSS | -0.13  [-0.26, 0.00] | -0.11  [-0.25, 0.02] | -0.10  [-0.23, 0.04] | -0.11  [-0.25, 0.03] | -0.09  [-0.23, 0.04] |
| Log Income | 0.31  [-0.18, 0.80] |  |  | 0.27  [-0.24, 0.78] |  |
| Education | 0.23 ^*^ [0.03, 0.42] |  | 0.24 ^*^ [0.04, 0.45] | 0.24 ^*^ [0.03, 0.44] |  |
| Political | -0.87 ^***^ [-1.01, -0.72] | -0.87 ^***^ [-1.01, -0.72] | -0.91 ^***^ [-1.05, -0.76] | -0.91 ^***^ [-1.05, -0.76] | -0.91 ^***^ [-1.06, -0.76] |
| Age | 0.00  [-0.01, 0.01] | 0.00  [-0.01, 0.01] | 0.00  [-0.01, 0.01] | -0.00  [-0.01, 0.01] | -0.00  [-0.01, 0.01] |
| Gender | -0.06  [-0.43, 0.31] | -0.07  [-0.44, 0.30] | -0.10  [-0.48, 0.28] | -0.10  [-0.48, 0.28] | -0.10  [-0.49, 0.28] |
| Income |  | 0.00  [-0.01, 0.02] | 0.00  [-0.01, 0.02] |  | 0.00  [-0.01, 0.02] |
| Educ [secondary] |  | -0.21  [-0.77, 0.35] |  |  | -0.19  [-0.77, 0.38] |
| Educ [post-16] |  | -0.29 ^*^ [-0.53, -0.05] |  |  | -0.31 ^*^ [-0.56, -0.06] |
| Educ [bachelor] |  | 0.23 ^*^ [0.01, 0.45] |  |  | 0.24 ^*^ [0.00, 0.47] |
| Educ [master] |  | 0.09  [-0.32, 0.51] |  |  | 0.12  [-0.31, 0.55] |
| Educ [doctoral] |  | 0.96  [-0.11, 2.03] |  |  | 0.97  [-0.14, 2.07] |
| *N* | 448 | 448 | 448 | 448 | 448 |
| *R^2^/R^2^_adj_* | 0.336 / 0.325 | 0.340 / 0.324 | 0.345 / 0.334 | 0.346 / 0.336 | 0.350 / 0.334 |
| * p<0.05   ** p<0.01   *** p<0.001 | | | | | |

**Table S15.** *Additional Regression Models for Study 4, Internal-to-External Explanations for Being Poor (Unstandardised Estimates)*

| Predictor | **Log Income** | **Education**  **as Factor** | **Robust** | **Robust**  **Log Income** | **Robust Education**  **as Factor** |
| --- | --- | --- | --- | --- | --- |
| (Intercept) | 5.48 ^***^ [4.15, 6.81] | 6.25 ^***^ [5.02, 7.47] | 5.98 ^***^ [4.61, 7.34] | 5.82 ^***^ [4.47, 7.17] | 6.67 ^***^ [5.42, 7.92] |
| PRD | -0.17  [-0.36, 0.02] | -0.17  [-0.36, 0.03] | -0.22 ^*^ [-0.41, -0.02] | -0.21 ^*^ [-0.41, -0.02] | -0.21 ^*^ [-0.41, -0.01] |
| SSS | -0.16 ^*^ [-0.29, -0.03] | -0.14 ^*^ [-0.27, -0.01] | -0.15 ^*^ [-0.28, -0.02] | -0.17 ^*^ [-0.30, -0.04] | -0.15 ^*^ [-0.28, -0.02] |
| Log Income | 0.38  [-0.10, 0.87] |  |  | 0.41  [-0.08, 0.90] |  |
| Education | 0.15  [-0.05, 0.34] |  | 0.18  [-0.02, 0.38] | 0.15  [-0.05, 0.35] |  |
| Political | -0.90 ^***^ [-1.04, -0.76] | -0.90 ^***^ [-1.04, -0.76] | -0.94 ^***^ [-1.08, -0.80] | -0.95 ^***^ [-1.09, -0.80] | -0.94 ^***^ [-1.08, -0.80] |
| Age | -0.00  [-0.01, 0.01] | -0.00  [-0.01, 0.01] | -0.00  [-0.02, 0.01] | -0.00  [-0.02, 0.01] | -0.00  [-0.02, 0.01] |
| Gender | -0.02  [-0.38, 0.35] | -0.02  [-0.39, 0.35] | -0.03  [-0.40, 0.34] | -0.03  [-0.40, 0.34] | -0.03  [-0.40, 0.35] |
| Income |  | 0.00  [-0.01, 0.02] | 0.00  [-0.01, 0.02] |  | 0.00  [-0.01, 0.01] |
| Educ [secondary] |  | -0.24  [-0.79, 0.32] |  |  | -0.21  [-0.77, 0.35] |
| Educ [post-16] |  | -0.22  [-0.45, 0.02] |  |  | -0.25 ^*^ [-0.49, -0.01] |
| Educ [bachelor] |  | 0.19  [-0.03, 0.41] |  |  | 0.19  [-0.03, 0.42] |
| Educ [master] |  | 0.06  [-0.35, 0.47] |  |  | 0.11  [-0.31, 0.53] |
| Educ [doctoral] |  | 0.56  [-0.50, 1.62] |  |  | 0.61  [-0.48, 1.69] |
| *N* | 448 | 448 | 448 | 448 | 448 |
| *R^2^/R^2^_adj_* | 0.325 / 0.314 | 0.326 / 0.309 | 0.341 / 0.331 | 0.346 / 0.335 | 0.346 / 0.330 |
| * p<0.05   ** p<0.01   *** p<0.001 | | | | | |

**Table S16.** *Standardised Regression Coefficients for Analyses Predicting Perceived Constraints and Personal Mastery (Studies 1 and 7, Time 1)*

|  | **Study 1 PC** | **Study 1 PM** | **Study 1 PC(+PM)** | **Study 1 PM(+PC)** | **Study 7 PC** | **Study 7 PM** | **Study 7 PC(+PM)** | **Study 7 PM(+PC)** |
| --- | --- | --- | --- | --- | --- | --- | --- | --- |
| PRD | 0.60 ^***^ [0.52 – 0.69] | -0.46 ^***^ [-0.56 – -0.37] | 0.35 ^***^ [0.28 – 0.42] | -0.05 [-0.13 – 0.04] | 0.42 ^***^ [0.34 – 0.51] | -0.30 ^***^ [-0.39 – -0.21] | 0.26 ^***^ [0.19 – 0.34] | -0.05 [-0.13 – 0.03] |
| SSS | -0.03 [-0.13 – 0.06] | 0.07 [-0.03 – 0.18] | 0.01 [-0.06 – 0.08] | 0.05 [-0.03 – 0.13] | -0.20 ^***^ [-0.29 – -0.11] | 0.20 ^***^ [0.10 – 0.30] | -0.10 ^*^ [-0.17 – -0.02] | 0.08 [-0.00 – 0.17] |
| Income | -0.10 ^**^ [-0.18 – -0.03] | 0.09 ^*^ [0.00 – 0.18] | -0.05 [-0.12 – 0.01] | 0.02 [-0.05 – 0.09] | 0.03 [-0.05 – 0.11] | -0.02 [-0.11 – 0.06] | 0.02 [-0.04 – 0.09] | -0.00 [-0.07 – 0.07] |
| Education | 0.03 [-0.04 – 0.11] | -0.08 [-0.16 – 0.00] | -0.01 [-0.07 – 0.05] | -0.06 [-0.12 – 0.01] | -0.02 [-0.10 – 0.06] | 0.06 [-0.02 – 0.15] | 0.01 [-0.06 – 0.08] | 0.05 [-0.02 – 0.12] |
| Political | -0.06 [-0.13 – 0.01] | 0.13 ^**^ [0.05 – 0.21] | 0.01 [-0.04 – 0.07] | 0.09 ^**^ [0.03 – 0.15] | -0.07 [-0.14 – 0.01] | 0.13 ^**^ [0.05 – 0.21] | 0.00 [-0.06 – 0.07] | 0.09 ^**^ [0.02 – 0.16] |
| Age | -0.02 [-0.10 – 0.05] | -0.03 [-0.11 – 0.05] | -0.04 [-0.10 – 0.02] | -0.04 [-0.11 – 0.02] | 0.01 [-0.07 – 0.08] | -0.04 [-0.12 – 0.04] | -0.01 [-0.08 – 0.05] | -0.03 [-0.10 – 0.03] |
| Gender | 0.08 ^*^ [0.01 – 0.15] | -0.06 [-0.13 – 0.02] | 0.05 [-0.01 – 0.10] | -0.00 [-0.06 – 0.06] | -0.07 ^*^ [-0.15 – -0.00] | -0.02 [-0.10 – 0.05] | -0.09 ^**^ [-0.15 – -0.02] | -0.07 ^*^ [-0.13 – -0.00] |
| PM |  |  | -0.55 ^***^ [-0.62 – -0.49] |  |  |  | -0.53 ^***^ [-0.59 – -0.46] |  |
| PC |  |  |  | -0.69 ^***^ [-0.77 – -0.61] |  |  |  | -0.59 ^***^ [-0.67 – -0.52] |
| Observations | 464 | 464 | 464 | 464 | 530 | 530 | 530 | 530 |
| R^2^ / R^2^ adjusted | 0.455 / 0.446 | 0.319 / 0.309 | 0.662 / 0.656 | 0.579 / 0.571 | 0.312 / 0.302 | 0.222 / 0.211 | 0.527 / 0.519 | 0.465 / 0.457 |

*Note.* PC = Perceived constraints. PM = Personal Mastery. 95% CIs are shown in brackets. * p<0.05   ** p<0.01   *** p<0.001

**Table S17.** *Descriptive Statistics and Correlations among Measures (Study 5)*

| Variable | *M* | *SD* | 1 | 2 | 3 | 4 | 5 | 6 | 7 | 8 | 9 |
| --- | --- | --- | --- | --- | --- | --- | --- | --- | --- | --- | --- |
| 1. External | 4.02 | 1.04 |  |  |  |  |  |  |  |  |  |
|  |  |  |  |  |  |  |  |  |  |  |  |
| 2. Internal | 4.44 | 0.96 | -.25 (<.001) |  |  |  |  |  |  |  |  |
|  |  |  | [-.33, -.17] |  |  |  |  |  |  |  |  |
|  |  |  |  |  |  |  |  |  |  |  |  |
| 3. Ext - Int | -0.42 | 1.58 | .81 (<.001) | -.77 (<.001) |  |  |  |  |  |  |  |
|  |  |  | [.78, .84] | [-.80, -.73] |  |  |  |  |  |  |  |
|  |  |  |  |  |  |  |  |  |  |  |  |
| 4. PRD | 3.13 | 1.09 | .27 (<.001) | -.21 (<.001) | .31 (<.001) |  |  |  |  |  |  |
|  |  |  | [.19, .35] | [-.29, -.13] | [.23, .38] |  |  |  |  |  |  |
|  |  |  |  |  |  |  |  |  |  |  |  |
| 5. SSS | 4.99 | 1.83 | -.10 (.027) | .31 (<.001) | -.25 (<.001) | -.39 (<.001) |  |  |  |  |  |
|  |  |  | [-.18, -.01] | [.23, .38] | [-.33, -.17] | [-.46, -.32] |  |  |  |  |  |
|  |  |  |  |  |  |  |  |  |  |  |  |
| 6. Income | 30.35 | 22.19 | -.20 (<.001) | .09 (.048) | -.18 (<.001) | -.31 (<.001) | .41 (<.001) |  |  |  |  |
|  |  |  | [-.28, -.12] | [.00, .17] | [-.27, -.10] | [-.38, -.23] | [.34, .48] |  |  |  |  |
|  |  |  |  |  |  |  |  |  |  |  |  |
| 7. Education | 2.85 | 0.61 | .01 (.889) | .11 (.010) | -.06 (.142) | -.15 (<.001) | .34 (<.001) | .25 (<.001) |  |  |  |
|  |  |  | [-.08, .09] | [.03, .19] | [-.15, .02] | [-.23, -.06] | [.27, .41] | [.17, .33] |  |  |  |
|  |  |  |  |  |  |  |  |  |  |  |  |
| 8. Political | 3.89 | 1.61 | -.32 (<.001) | .33 (<.001) | -.41 (<.001) | -.19 (<.001) | .11 (.013) | .01 (.871) | .08 (.077) |  |  |
|  |  |  | [-.39, -.24] | [.25, .40] | [-.48, -.34] | [-.27, -.10] | [.02, .19] | [-.08, .09] | [-.01, .16] |  |  |
|  |  |  |  |  |  |  |  |  |  |  |  |
| 9. Age | 38.21 | 12.29 | -.07 (.115) | .12 (.004) | -.12 (.005) | -.19 (<.001) | .07 (.131) | .06 (.139) | .10 (.017) | .11 (.010) |  |
|  |  |  | [-.15, .02] | [.04, .21] | [-.20, -.04] | [-.27, -.11] | [-.02, .15] | [-.02, .15] | [.02, .19] | [.03, .19] |  |
|  |  |  |  |  |  |  |  |  |  |  |  |
| 10. Gender | -- | -- | .08 (.052) | -.10 (.023) | .11 (.008) | -.04 (.332) | -.01 (.855) | -.05 (.250) | .04 (.385) | -.03 (0.446) | .13 (.002) |
|  |  |  | [-.00, .17] | [-.18, -.01] | [.03, .20] | [-.13, .04] | [-.09, .08] | [-.13, .03] | [-.05, .12] | [-.12, .05] | [.05, .22] |

*Note*. Ext – Int = difference scores between external and internal explanations. PRD = Personal Relative Deprivation. SSS = Subjective Socioeconomic Status. Values in parentheses and brackets indicate *p* values and 95% confidence intervals, respectively. *N* = 540.

**Table S18.** *Kendall Correlations for Study 5*

| Variable | External | Internal | Ext - Int | PRD | SSS | Income | Education | Political | Age |
| --- | --- | --- | --- | --- | --- | --- | --- | --- | --- |
| Internal | -.25 (<.001) |  |  |  |  |  |  |  |  |
|  | [-.32, -.17] |  |  |  |  |  |  |  |  |
| Ext - Int | .68 (<.001) | -.60 (<.001) |  |  |  |  |  |  |  |
|  | [.64, .71] | [-.65, -.55] |  |  |  |  |  |  |  |
| PRD | .20 (<.001) | -.14 (<.001) | .22 (<.001) |  |  |  |  |  |  |
|  | [.14, .26] | [-.20, -.08] | [.16, .28] |  |  |  |  |  |  |
| SSS | -.09 (.003) | .22 (<.001) | -.16 (<.001) | -.28 (<.001) |  |  |  |  |  |
|  | [-.15, -.03] | [.16, .28] | [-.22, -.10] | [-.34, -.22] |  |  |  |  |  |
| Income | -.15 (<.001) | .06 (.036) | -.14 (<.001) | -.22 (<.001) | .31 (<.001) |  |  |  |  |
|  | [-.21, -.10] | [.00, .12] | [-.20, -.09] | [-.27, -.16] | [.26, .37] |  |  |  |  |
| Education | -.01 (.868) | .10 (.005) | -.06 (.065) | -.10 (.003) | .29 (<.001) | .20 (<.001) |  |  |  |
|  | [-.07, .06] | [.03, .16] | [-.13, .01] | [-.18, -.03] | [.23, .36] | [.14, .26] |  |  |  |
| Political | -.27 (<.001) | .26 (<.001) | -.30 (<.001) | -.13 (<.001) | .08 (.014) | .02 (.472) | .06 (.112) |  |  |
|  | [-.34, -.21] | [.20, .33] | [-.36, -.24] | [-.19, -.06] | [.01, .15] | [-.04, .09] | [-.01, .13] |  |  |
| Age | -.08 (.008) | .09 (.003) | -.09 (.001) | -.14 (<.001) | .05 (.132) | .05 (.073) | .06 (.069) | .10 (.001) |  |
|  | [-.14, -.02] | [.03, .15] | [-.15, -.04] | [-.19, -.08] | [-.01, .11] | [-.01, .11] | [-.01, .13] | [.04, .16] |  |
| Gender | .08 (.030) | -.08 (.019) | .09 (.009) | -.03 (.369) | .00 (.954) | -.04 (.271) | .04 (.390) | -.03 (.425) | .11 (.002) |
|  | [.01, .15] | [-.15, -.02] | [.02, .16] | [-.10, .04] | [-.07, .07] | [-.11, .03] | [-.05, .12] | [-.11, .05] | [.04, .18] |

*Note*. Ext – Int = difference between external and internal explanations. PRD = Personal Relative Deprivation. SSS = Subjective Socioeconomic Status. Values in parentheses and brackets indicate *p* values and 95% confidence intervals, respectively.

**Table S19.** *Additional Regression Models for Study 5, External Explanations (Unstandardised Estimates)*

| Predictors | **Log Income** | **Education**  **as Factor** | **Robust** | **Robust**  **Log Income** | **Robust Education**  **as Factor** |
| --- | --- | --- | --- | --- | --- |
| Intercept | 4.49 ^***^ [3.81, 5.17] | 4.24 ^***^ [3.67, 4.82] | 4.22 ^***^ [3.62, 4.82] | 4.70 ^***^ [4.04, 5.36] | 4.53 ^***^ [3.98, 5.08] |
| PRD | 0.19 ^***^ [0.11, 0.27] | 0.19 ^***^ [0.11, 0.27] | 0.17 ^***^ [0.09, 0.25] | 0.17 ^***^ [0.09, 0.25] | 0.17 ^***^ [0.09, 0.25] |
| SSS | 0.04  [-0.01, 0.09] | 0.03  [-0.02, 0.09] | 0.02  [-0.03, 0.07] | 0.02  [-0.03, 0.07] | 0.01  [-0.04, 0.06] |
| Log Income | -0.67 ^***^ [-0.93, -0.41] |  |  | -0.56 ^***^ [-0.82, -0.31] |  |
| Education | 0.14  [-0.00, 0.28] |  | 0.11  [-0.02, 0.24] | 0.12  [-0.01, 0.25] |  |
| Political | -0.18 ^***^ [-0.23, -0.13] | -0.19 ^***^ [-0.24, -0.13] | -0.22 ^***^ [-0.27, -0.17] | -0.22 ^***^ [-0.26, -0.17] | -0.22 ^***^ [-0.27, -0.17] |
| Age | -0.00  [-0.01, 0.01] | -0.00  [-0.01, 0.01] | -0.00  [-0.01, 0.00] | -0.00  [-0.01, 0.00] | -0.00  [-0.01, 0.01] |
| Gender | 0.14  [-0.02, 0.31] | 0.16  [-0.01, 0.32] | 0.18 ^*^ [0.02, 0.34] | 0.16 ^*^ [0.01, 0.32] | 0.18 ^*^ [0.02, 0.34] |
| Income |  | -0.01 ^***^ [-0.01, -0.00] | -0.01 ^***^ [-0.01, -0.00] |  | -0.01 ^***^ [-0.01, -0.00] |
| Educ [high school] |  | -0.12  [-0.27, 0.02] |  |  | -0.10  [-0.24, 0.04] |
| Educ [college] |  | 0.04  [-0.02, 0.10] |  |  | 0.04  [-0.02, 0.10] |
| Educ [postgrad] |  | 0.09  [-0.13, 0.32] |  |  | 0.05  [-0.17, 0.27] |
| *N* | 540 | 540 | 540 | 540 | 540 |
| *R^2^/R^2^_adj_* | 0.196 / 0.186 | 0.185 / 0.171 | 0.223 / 0.212 | 0.229 / 0.219 | 0.226 / 0.213 |
| * p<0.05   ** p<0.01   *** p<0.001 | | | | | |

**Table S20.** *Additional Regression Models for Study 5, Internal Explanations (Unstandardised Estimates)*

| Predictors | **Log Income** | **Education**  **as Factor** | **Robust** | **Robust**  **Log Income** | **Robust Education**  **as Factor** |
| --- | --- | --- | --- | --- | --- |
| Intercept | 3.17 ^***^ [2.54, 3.80] | 3.06 ^***^ [2.54, 3.59] | 3.04 ^***^ [2.49, 3.58] | 3.14 ^***^ [2.54, 3.74] | 3.00 ^***^ [2.50, 3.50] |
| PRD | -0.05  [-0.13, 0.02] | -0.05  [-0.13, 0.02] | -0.06  [-0.13, 0.02] | -0.06  [-0.13, 0.02] | -0.06  [-0.13, 0.02] |
| SSS | 0.14 ^***^ [0.09, 0.19] | 0.14 ^***^ [0.09, 0.19] | 0.13 ^***^ [0.09, 0.18] | 0.13 ^***^ [0.09, 0.18] | 0.13 ^***^ [0.09, 0.18] |
| Log Income | -0.11  [-0.35, 0.13] |  |  | -0.12  [-0.35, 0.11] |  |
| Education | -0.01  [-0.14, 0.12] |  | -0.01  [-0.13, 0.11] | -0.01  [-0.14, 0.11] |  |
| Political | 0.17 ^***^ [0.12, 0.21] | 0.17 ^***^ [0.12, 0.22] | 0.19 ^***^ [0.14, 0.23] | 0.19 ^***^ [0.14, 0.23] | 0.19 ^***^ [0.14, 0.23] |
| Age | 0.01 ^*^ [0.00, 0.01] | 0.01 ^*^ [0.00, 0.01] | 0.01 ^*^ [0.00, 0.01] | 0.01 ^*^ [0.00, 0.01] | 0.01 ^*^ [0.00, 0.01] |
| Gender | -0.20 ^**^ [-0.35, -0.05] | -0.20 ^**^ [-0.35, -0.05] | -0.22 ^**^ [-0.37, -0.08] | -0.22 ^**^ [-0.37, -0.08] | -0.23 ^**^ [-0.37, -0.08] |
| Income |  | -0.00  [-0.01, 0.00] | -0.00  [-0.01, 0.00] |  | -0.00  [-0.01, 0.00] |
| Educ [high school] |  | -0.04  [-0.17, 0.10] |  |  | -0.02  [-0.15, 0.10] |
| Educ [college] |  | 0.02  [-0.04, 0.08] |  |  | 0.01  [-0.04, 0.07] |
| Educ [postgrad] |  | -0.05  [-0.26, 0.16] |  |  | -0.03  [-0.23, 0.16] |
| *N* | 540 | 540 | 540 | 540 | 540 |
| *R^2^/R^2^_adj_* | 0.202 / 0.192 | 0.205 / 0.192 | 0.236 / 0.226 | 0.235 / 0.225 | 0.238 / 0.225 |
| * p<0.05   ** p<0.01   *** p<0.001 | | | | | |

**Table S21.** *Additional Regression Models for Study 5, External – Internal Explanations (Unstandardised Estimates)*

| Predictors | **Log Income** | **Education**  **as Factor** | **Robust** | **Robust**  **Log Income** | **Robust Education**  **as Factor** |
| --- | --- | --- | --- | --- | --- |
| Intercept | 1.32 ^**^ [0.33, 2.31] | 1.18 ^**^ [0.35, 2.01] | 0.81  [-0.04, 1.67] | 1.29 ^**^ [0.34, 2.23] | 1.07 ^**^ [0.28, 1.86] |
| PRD | 0.24 ^***^ [0.12, 0.36] | 0.25 ^***^ [0.13, 0.37] | 0.24 ^***^ [0.12, 0.36] | 0.24 ^***^ [0.12, 0.35] | 0.24 ^***^ [0.12, 0.35] |
| SSS | -0.10 ^*^ [-0.17, -0.02] | -0.10 ^**^ [-0.18, -0.03] | -0.09 ^**^ [-0.17, -0.02] | -0.09 ^*^ [-0.16, -0.02] | -0.09 ^*^ [-0.16, -0.02] |
| Log Income | -0.56 ^**^ [-0.94, -0.18] |  |  | -0.51 ^**^ [-0.87, -0.15] |  |
| Education | 0.15  [-0.06, 0.35] |  | 0.09  [-0.10, 0.28] | 0.10  [-0.09, 0.29] |  |
| Political | -0.35 ^***^ [-0.42, -0.28] | -0.35 ^***^ [-0.43, -0.28] | -0.33 ^***^ [-0.40, -0.26] | -0.33 ^***^ [-0.40, -0.26] | -0.34 ^***^ [-0.41, -0.27] |
| Age | -0.01  [-0.02, 0.00] | -0.01  [-0.02, 0.00] | -0.01  [-0.01, 0.00] | -0.01  [-0.02, 0.00] | -0.01  [-0.01, 0.00] |
| Gender | 0.34 ^**^ [0.10, 0.58] | 0.36 ^**^ [0.12, 0.60] | 0.34 ^**^ [0.12, 0.57] | 0.33 ^**^ [0.11, 0.56] | 0.35 ^**^ [0.12, 0.57] |
| Income |  | -0.01 ^*^ [-0.01, -0.00] | -0.01 ^*^ [-0.01, -0.00] |  | -0.01 ^*^ [-0.01, -0.00] |
| Educ [high school] |  | -0.09  [-0.30, 0.12] |  |  | -0.03  [-0.23, 0.17] |
| Educ [college] |  | 0.02  [-0.07, 0.11] |  |  | 0.00  [-0.08, 0.09] |
| Educ [postgrad] |  | 0.14  [-0.19, 0.47] |  |  | 0.10  [-0.21, 0.40] |
| *N* | 540 | 540 | 540 | 540 | 540 |
| *R^2^/R^2^_adj_* | 0.267 / 0.257 | 0.263 / 0.250 | 0.268 / 0.258 | 0.271 / 0.262 | 0.271 / 0.258 |
| * p<0.05   ** p<0.01   *** p<0.001 | | | | | |

**Figure S3.** *Graphical Representation of the RI-CLPM with Personal Relative Deprivation, Perceived Constraints, and Personal Mastery across the First Three Timepoints in Studies 7 and 8*

*Note.* RI-CLPM = Random Intercept Cross-Lagged Panel Model; PRD = Personal Relative Deprivation; PC = Perceived Constraints (external locus of control); PM = Personal Mastery (internal locus of control); T = Timepoint. Blue circles represent between-person components; pink circles represent within-person components; white circles represent occasion-specific residuals.

**Table S22.** *Descriptive Statistics and Correlations among Measures across Timepoints (Study 7)*

| Variable | *M* | *SD* | 1 | 2 | 3 | 4 | 5 | 6 | 7 | 8 | 9 | 10 | 11 | 12 |
| --- | --- | --- | --- | --- | --- | --- | --- | --- | --- | --- | --- | --- | --- | --- |
|  |  |  |  |  |  |  |  |  |  |  |  |  |  |  |
| 1. PRD_T1 | 2.92 | 1.00 | (.82/.75*) |  |  |  |  |  |  |  |  |  |  |  |
|  |  |  |  |  |  |  |  |  |  |  |  |  |  |  |
| 2. PRD_T2 | 2.89 | 1.01 | .79 | (.84/.60*) |  |  |  |  |  |  |  |  |  |  |
|  |  |  | [.75, .82] |  |  |  |  |  |  |  |  |  |  |  |
| 3. PRD_T3 | 2.89 | 1.02 | .80 | .84 | (.86/.59*) |  |  |  |  |  |  |  |  |  |
|  |  |  | [.76, .83] | [.81, .87] |  |  |  |  |  |  |  |  |  |  |
| 4. PRD_T4 | 2.83 | 1.00 | .79 | .85 | .85 | (.85/.77*) |  |  |  |  |  |  |  |  |
|  |  |  | [.76, .83] | [.83, .88] | [.83, .88] |  |  |  |  |  |  |  |  |  |
| 5. PC_T1 | 3.57 | 1.27 | .52 | .52 | .56 | .59 | (.89/.81) |  |  |  |  |  |  |  |
|  |  |  | [.46, .58] | [.45, .58] | [.50, .62] | [.52, .65] |  |  |  |  |  |  |  |  |
| 6. PC_T2 | 3.59 | 1.28 | .50 | .57 | .58 | .58 | .82 | (.90/.82*) |  |  |  |  |  |  |
|  |  |  | [.42, .56] | [.50, .63] | [.51, .64] | [.51, .64] | [.79, .85] |  |  |  |  |  |  |  |
| 7. PC_T3 | 3.62 | 1.28 | .54 | .57 | .62 | .62 | .83 | .84 | (.89/.81) |  |  |  |  |  |
|  |  |  | [.48, .61] | [.50, .63] | [.56, .67] | [.55, .67] | [.80, .86] | [.81, .87] |  |  |  |  |  |  |
| 8. PC_T4 | 3.65 | 1.35 | .50 | .56 | .58 | .64 | .82 | .84 | .87 | (.92/.84) |  |  |  |  |
|  |  |  | [.43, .57] | [.49, .62] | [.52, .64] | [.58, .69] | [.78, .85] | [.81, .86] | [.85, .89] |  |  |  |  |  |
| 9. PM_T1 | 4.90 | 1.06 | -.41 | -.42 | -.49 | -.50 | -.66 | -.60 | -.63 | -.62 | (.80/.74) |  |  |  |
|  |  |  | [-.47, -.33] | [-.50, -.35] | [-.55, -.41] | [-.57, -.43] | [-.71, -.61] | [-.66, -.54] | [-.68, -.57] | [-.67, -.56] |  |  |  |  |
| 10. PM_T2 | 4.94 | 1.05 | -.40 | -.48 | -.47 | -.50 | -.59 | -.64 | -.62 | -.59 | .72 | (.81/.72*) |  |  |
|  |  |  | [-.48, -.33] | [-.55, -.41] | [-.54, -.39] | [-.57, -.43] | [-.65, -.53] | [-.69, -.58] | [-.67, -.55] | [-.65, -.52] | [.67, .76] |  |  |  |
| 11. PM_T3 | 4.92 | 1.10 | -.44 | -.47 | -.49 | -.54 | -.62 | -.62 | -.66 | -.66 | .76 | .73 | (.84/.77) |  |
|  |  |  | [-.51, -.37] | [-.54, -.40] | [-.56, -.42] | [-.60, -.47] | [-.67, -.56] | [-.68, -.56] | [-.71, -.61] | [-.71, -.61] | [.72, .80] | [.68, .77] |  |  |
| 12. PM_T4 | 4.92 | 1.11 | -.42 | -.45 | -.48 | -.54 | -.61 | -.60 | -.68 | -.67 | .74 | .69 | .76 | (.84/.76) |
|  |  |  | [-.50, -.34] | [-.53, -.37] | [-.55, -.40] | [-.61, -.47] | [-.67, -.55] | [-.66, -.54] | [-.73, -.62] | [-.72, -.61] | [.69, .78] | [.64, .74] | [.72, .80] |  |
|  |  |  |  |  |  |  |  |  |  |  |  |  |  |  |

*Note.* PRD = Personal Relative Deprivation. PC = Perceived Constraints (external). PM = Personal Mastery (internal). T1 – T4 = first time point to fourth time point. Values in square brackets indicate the 95% confidence interval for each correlation. Alpha/Omega_h_ reliabilities are shown in parentheses along the diagonal (we use an asterisk to indicate problematic estimates of ω_h_ because the software produced warnings during the calculation). All correlations are statistically significant at *p* < .001.

**Table S23.** *Descriptive Statistics and Reliabilities for Daily Diary Measures across Days (Study 8)*

| Measure | N | M | SD | α |  | Measure | M | SD | α |  | Measure | M | SD | α |
| --- | --- | --- | --- | --- | --- | --- | --- | --- | --- | --- | --- | --- | --- | --- |
| T1_PRD | 181 | 2.41 | 1.042 | 0.88 |  | T1_PC | 2.52 | 1.075 | 0.74 |  | T1_PM | 3.58 | 0.919 | 0.77 |
| T2_PRD | 171 | 2.29 | 0.938 | 0.82 |  | T2_PC | 2.44 | 1.061 | 0.75 |  | T2_PM | 3.67 | 0.941 | 0.75 |
| T3_PRD | 175 | 2.36 | 0.962 | 0.82 |  | T3_PC | 2.59 | 1.072 | 0.7 |  | T3_PM | 3.57 | 0.901 | 0.78 |
| T4_PRD | 171 | 2.25 | 0.904 | 0.81 |  | T4_PC | 2.56 | 1.020 | 0.62 |  | T4_PM | 3.61 | 0.868 | 0.73 |
| T5_PRD | 165 | 2.21 | 0.911 | 0.84 |  | T5_PC | 2.51 | 1.067 | 0.71 |  | T5_PM | 3.47 | 1.002 | 0.79 |
| T6_PRD | 161 | 2.14 | 0.835 | 0.79 |  | T6_PC | 2.34 | 1.007 | 0.74 |  | T6_PM | 3.64 | 0.918 | 0.83 |
| T7_PRD | 165 | 2.24 | 0.909 | 0.83 |  | T7_PC | 2.59 | 1.093 | 0.74 |  | T7_PM | 3.58 | 0.986 | 0.83 |
| T8_PRD | 172 | 2.28 | 0.947 | 0.82 |  | T8_PC | 2.76 | 1.041 | 0.73 |  | T8_PM | 3.50 | 0.902 | 0.78 |
| T9_PRD | 169 | 2.24 | 0.976 | 0.86 |  | T9_PC | 2.62 | 1.042 | 0.63 |  | T9_PM | 3.52 | 0.907 | 0.85 |
| T10_PRD | 167 | 2.31 | 0.962 | 0.85 |  | T10_PC | 2.70 | 1.046 | 0.8 |  | T10_PM | 3.46 | 0.947 | 0.8 |
| T11_PRD | 170 | 2.34 | 1.000 | 0.83 |  | T11_PC | 2.68 | 1.017 | 0.72 |  | T11_PM | 3.51 | 0.892 | 0.85 |
| T12_PRD | 162 | 2.26 | 0.974 | 0.86 |  | T12_PC | 2.73 | 1.062 | 0.77 |  | T12_PM | 3.41 | 1.004 | 0.81 |
| T13_PRD | 159 | 2.27 | 0.958 | 0.83 |  | T13_PC | 2.59 | 1.063 | 0.77 |  | T13_PM | 3.62 | 0.915 | 0.78 |
| T14_PRD | 159 | 2.16 | 0.916 | 0.82 |  | T14_PC | 2.52 | 1.055 | 0.78 |  | T14_PM | 3.60 | 0.921 | 0.87 |

*Note.* PRD = Personal Relative Deprivation. PC = Perceived Constraints (external). PM = Personal Mastery (internal). T1 – T14 = first day to fourteenth day. α = Cronbach’s alpha.

**Table S24.** *Unstandardised Estimates for the Residual Covariances by Day for Study 8*

| Residual covariances | *B* | *SE* | *Z* | *p* | 95% CI_LL_ | 95% CI_UL_ |
| --- | --- | --- | --- | --- | --- | --- |
| PRD2~~PC2 | **0.168** | 0.037 | 4.603 | 4.16E-06 | 0.097 | 0.240 |
| PRD3~~PC3 | **0.117** | 0.041 | 2.887 | 3.88E-03 | 0.038 | 0.197 |
| PRD4~~PC4 | **0.186** | 0.037 | 5.063 | 4.13E-07 | 0.114 | 0.258 |
| PRD5~~PC5 | **0.154** | 0.038 | 4.011 | 6.05E-05 | 0.079 | 0.229 |
| PRD6~~PC6 | **0.143** | 0.038 | 3.770 | 1.63E-04 | 0.068 | 0.217 |
| PRD7~~PC7 | **0.173** | 0.040 | 4.315 | 1.60E-05 | 0.094 | 0.251 |
| PRD8~~PC8 | **0.115** | 0.035 | 3.241 | 1.19E-03 | 0.045 | 0.184 |
| PRD9~~PC9 | **0.076** | 0.033 | 2.268 | 2.33E-02 | 0.010 | 0.141 |
| PRD10~~PC10 | **0.102** | 0.036 | 2.825 | 4.73E-03 | 0.031 | 0.172 |
| PRD11~~PC11 | **0.166** | 0.039 | 4.252 | 2.12E-05 | 0.090 | 0.243 |
| PRD12~~PC12 | **0.113** | 0.032 | 3.498 | 4.69E-04 | 0.050 | 0.177 |
| PRD13~~PC13 | **0.199** | 0.042 | 4.754 | 1.99E-06 | 0.117 | 0.282 |
| PRD14~~PC14 | **0.101** | 0.029 | 3.450 | 5.60E-04 | 0.044 | 0.159 |
| PRD2~~PM2 | **-0.093** | 0.035 | -2.648 | 8.10E-03 | -0.162 | -0.024 |
| PRD3~~PM3 | -0.033 | 0.037 | -0.887 | 3.75E-01 | -0.105 | 0.039 |
| PRD4~~PM4 | **-0.144** | 0.034 | -4.205 | 2.61E-05 | -0.211 | -0.077 |
| PRD5~~PM5 | **-0.124** | 0.036 | -3.463 | 5.34E-04 | -0.193 | -0.054 |
| PRD6~~PM6 | **-0.088** | 0.034 | -2.580 | 9.87E-03 | -0.155 | -0.021 |
| PRD7~~PM7 | **-0.095** | 0.034 | -2.762 | 5.74E-03 | -0.163 | -0.028 |
| PRD8~~PM8 | -0.054 | 0.030 | -1.811 | 7.01E-02 | -0.112 | 0.004 |
| PRD9~~PM9 | -0.045 | 0.030 | -1.489 | 1.36E-01 | -0.105 | 0.014 |
| PRD10~~PM10 | **-0.131** | 0.034 | -3.861 | 1.13E-04 | -0.197 | -0.064 |
| PRD11~~PM11 | **-0.086** | 0.034 | -2.510 | 1.21E-02 | -0.153 | -0.019 |
| PRD12~~PM12 | **-0.122** | 0.034 | -3.602 | 3.16E-04 | -0.189 | -0.056 |
| PRD13~~PM13 | -0.046 | 0.034 | -1.367 | 1.72E-01 | -0.112 | 0.020 |
| PRD14~~PM14 | **-0.065** | 0.027 | -2.455 | 1.41E-02 | -0.118 | -0.013 |
| PC2~~PM2 | **-0.199** | 0.057 | -3.483 | 4.96E-04 | -0.311 | -0.087 |
| PC3~~PM3 | **-0.176** | 0.054 | -3.285 | 1.02E-03 | -0.281 | -0.071 |
| PC4~~PM4 | **-0.260** | 0.055 | -4.749 | 2.05E-06 | -0.368 | -0.153 |
| PC5~~PM5 | **-0.296** | 0.062 | -4.801 | 1.58E-06 | -0.417 | -0.175 |
| PC6~~PM6 | **-0.299** | 0.059 | -5.060 | 4.18E-07 | -0.415 | -0.183 |
| PC7~~PM7 | **-0.349** | 0.062 | -5.658 | 1.53E-08 | -0.470 | -0.228 |
| PC8~~PM8 | **-0.249** | 0.052 | -4.790 | 1.67E-06 | -0.351 | -0.147 |
| PC9~~PM9 | **-0.242** | 0.056 | -4.296 | 1.74E-05 | -0.353 | -0.132 |
| PC10~~PM10 | **-0.263** | 0.058 | -4.554 | 5.25E-06 | -0.377 | -0.150 |
| PC11~~PM11 | **-0.252** | 0.053 | -4.789 | 1.68E-06 | -0.356 | -0.149 |
| PC12~~PM12 | **-0.274** | 0.062 | -4.449 | 8.61E-06 | -0.395 | -0.153 |
| PC13~~PM13 | **-0.209** | 0.055 | -3.810 | 1.39E-04 | -0.316 | -0.101 |
| PC14~~PM14 | **-0.315** | 0.058 | -5.412 | 6.24E-08 | -0.429 | -0.201 |

*Note.* Residual covariances shown in bold indicate estimates where the corresponding 95% CI does not contain zero. PRD = Personal Relative Deprivation. PC = Perceived Constraints (external locus of control). PM = Personal Mastery (internal locus of control).

**Text S1: PRELIMINARY STUDY 6**

**Method**

**Participants**

Participants were recruited via MTurk. We requested 80 participants from the United States and with an approval rate greater than or equal to 98% on the platform. We discarded without analysis responses from participants who did not answer all questions, who indicated an age less than 18 or greater than 100, who provided a numeric response for their target’s initials, or whose IP address occurred earlier in the data set. We had N = 76 after applying these criteria (M_age_ = 36.00, SD_age_ = 11.09; 30% female). This sample size gave 80% and 90% power to detect Comparison Direction X Type of Explanation interaction effects of *dz* = .325 and .377, respectively [two-tailed, α = .05).

**Procedure and measures**

The procedure and measures for the preliminary study were the same as those for the main Study 6.

After identifying each comparison target, participants rated the extent to which they agreed that the target’s relative financial situation could be explained by internal factors [5 items: better skills, more ability, more motivation, more intelligence, a stronger work ethic; α_better_ = .91, ω_h.better_ = .81, α_lateral_ = .89, ω_h.lateral_* = .76) and external factors [5 items: had better luck, often been at the right place at the right time, had better opportunities because of their family background or upbringing, had financial assistance from family or friends, been provided with better support or connections; α_better_ = .85, ω_h.better_* = .78, α_lateral_ = .92, ω_h.lateral_* = .87).

**Results**

A 2 (Comparison direction: better off vs. lateral target) x 2 [Type of explanation: internal vs. external) repeated-measures ANOVA revealed significant main effects of comparison direction, *F*(1, 75) = 32.17, *p* < .001, *dz* = 0.65, 95% CI [0.40, 0.90], and type of explanation, *F*(1, 75) = 6.55, *p* = .01, *dz* = 0.29, 95% CI [0.06, 0.52]. There also was a significant interaction, *F*(1, 75) = 4.57, *p* = .036, *dz* = 0.25, 95% CI [0.02, 0.47] (see Figure S4). Follow-up paired-samples *t*-tests revealed that the effect of making a better off vs. lateral social comparison of affluence on explanations was larger for external explanations, *t*(75) = 5.13, *p* < .001, *dz* = 0.59, 97.5% CI [0.31, 0.87], than for internal explanations *t*(75) = 3.09, *p* = .006, *dz* = 0.35, 97.5% CI [0.09, 0.62] [*p* values and CIs were Bonferroni corrected for two comparisons).

**Figure S4.** *Effect of Comparison Direction as a Function of Type of Explanation (Preliminary Study)*


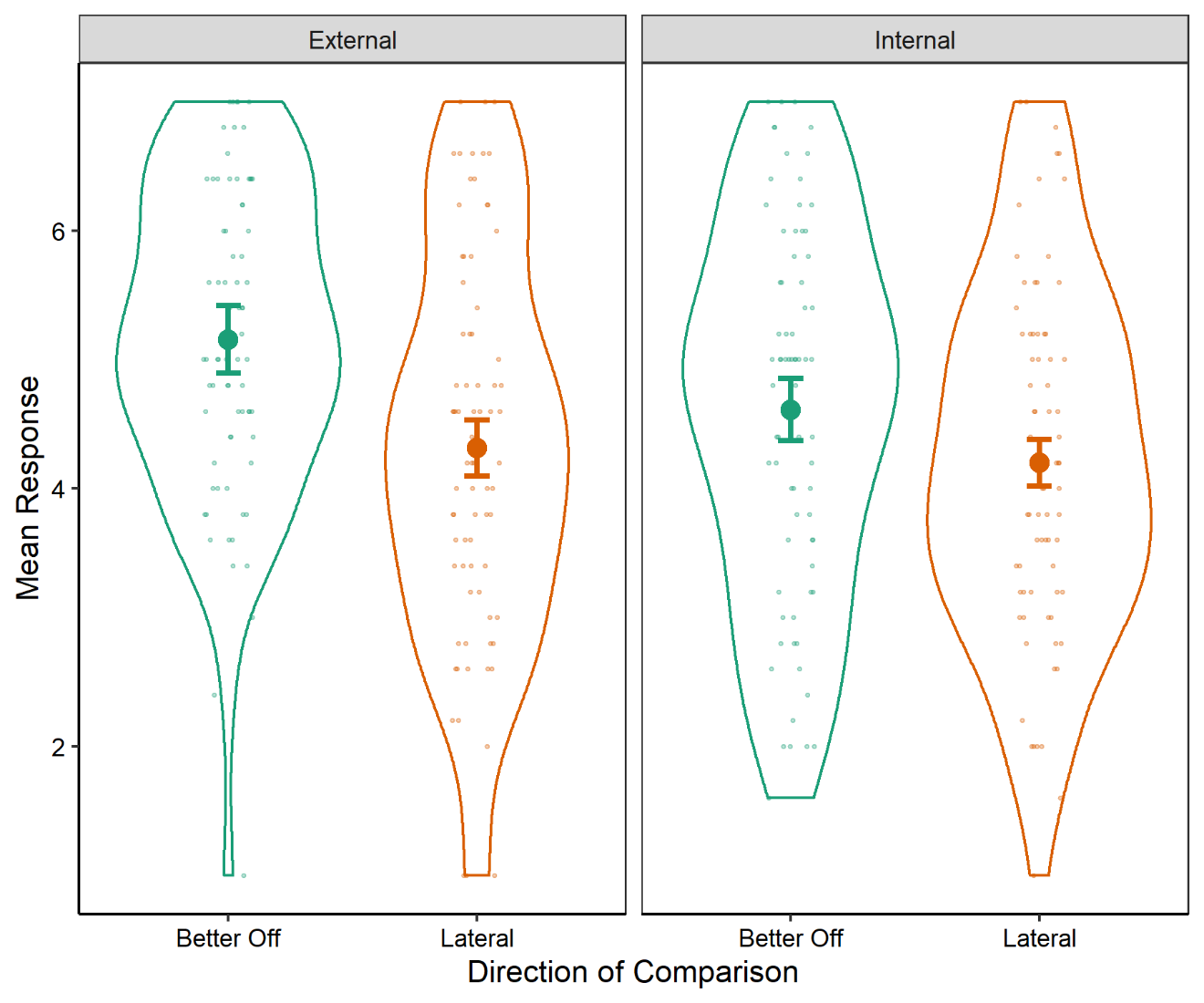


*Note.* Raw data, descriptive and inferential statistics plot of participants’ external and internal explanations as a function of making upward [identified target better off financially) and lateral [identified target just as well off financially) comparisons. The circles show mean explanations within conditions, and the error bars are within-subject 95% CIs [Morey, 2008).
